# Efficient Electrocatalytic Conversion of CO_2_ to Pure Formic Acid Solutions via Strain‐Engineered Bismuth Nanosheets

**DOI:** 10.1002/advs.75152

**Published:** 2026-04-09

**Authors:** Shiqi Li, Yuefeng Zhang, Panzhe Qiao, Cuo Wu, Xinya Tian, Yufeng Xu, Yun Zhang, Peng Wang, Tingting Li, Muhammad Afaq, Xinlin Luo, Tang Wang, Chunxiao Liu, Zi‐You Yu, Chuan Xia, Min‐Rui Gao, Zhen‐Yu Wu

**Affiliations:** ^1^ Department of Chemistry Institute of Innovative Material, Guangdong Provincial Key Laboratory of Sustainable Biomimetic Materials and Green Energy, State Key Laboratory of Quantum Functional Materials Southern University of Science and Technology Shenzhen China; ^2^ Shanghai Synchrotron Radiation Facility, Shanghai Advanced Research Institute Chinese Academy of Sciences Shanghai China; ^3^ MOE International Joint Laboratory of Materials Microstructure Institute For New Energy Materials and Low Carbon Technologies School of Materials Science & Engineering Tianjin University of Technology Tianjin China; ^4^ School of Materials and Energy University of Electronic Science and Technology of China Chengdu China; ^5^ Division of Nanomaterials & Chemistry Hefei National Research Center for Physical Sciences at the Microscale University of Science and Technology of China Hefei China

**Keywords:** bismuth, CO_2_ electroreduction, pure formic acid solution, solid electrolyte, strain engineering

## Abstract

The electrochemical reduction of CO_2_ to formic acid (HCOOH), a valuable chemical feedstock, offers a promising pathway toward carbon neutrality. However, achieving high selectivity, industrial current densities, long‐time stability, and the direct production of pure products simultaneously remains difficult. Here we present tensile‐strained bismuth nanosheets (TS‐BiNs) created through a scalable mechanochemical ball‐milling and carbonation process, effectively addressing all of these challenges. The TS‐BiNs catalyst demonstrates outstanding performance, maintaining over 92% Faradaic efficiency for HCOO^−^ across a wide current density range from −50 to −1000 mA cm^−2^ and an excellent stability at −100 mA cm^−2^ for over 100 h. When used in a solid‐state electrolyte reactor (4 cm^2^), it ensures the continuous and stable production of pure HCOOH solutions with widely adjustable concentrations (40–1500 mm) at 400 mA over 100 h. Combined experimental and theoretical analyses reveal that the tensile strain enhances the adsorption of the ^*^OCHO intermediate and inhibits the competing hydrogen evolution reaction, thereby directing the process toward highly efficient HCOOH formation. This work underscores the significant potential of strain engineering in catalyst design and introduces an integrated catalyst‐reactor strategy for practical electrochemical CO_2_ upgrading.

## Graphical abstract

Tensile‐strained bismuth nanosheets (TS‐BiNs) achieve a formate Faradaic efficiency of 92% at −1000 mA cm^−2^ and enable stable production of pure formic acid (HCOOH) in a solid‐state electrolyte reactor. Theoretical calculations reveal that the introduced strain lowers the energy barrier for ^*^OCHO intermediate formation, fundamentally enhancing the activity, selectivity, and stability for CO_2_‐to‐HCOOH conversion.

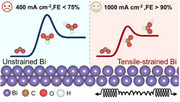

## 1 Introduction

The electrochemical carbon dioxide reduction reaction (CO_2_RR) to value‐added chemicals represents a promising strategy for closing the carbon cycle while storing renewable energy [1, 2, 3, 4, 5]. Among various CO_2_RR products, hydrocarbons (e.g., ethylene) and alcohols (e.g., methanol, ethanol, and propanol) have attracted considerable interest due to their high energy density and substantial market demand [6, 7, 8, 9, 10]. However, their synthesis typically involves multiple complex proton‐coupled electron transfer processes, leading to limited selectivity and efficiency. In contrast, formic acid (HCOOH) electrosynthesis follows a more straightforward two‐electron pathway, enabling a much higher Faradaic efficiency (FE) (over 90%) and more favorable kinetics [11, 12]. Moreover, HCOOH holds particular industrial importance because of its wide applications in hydrogen storage, fuel cells, and as a key feedstock for agriculture and pharmaceuticals [13, 14, 15]. Yet, practical CO_2_‐to‐HCOOH conversion faces critical challenges in high‐performance catalyst design, system efficiency, and product separation, calling for innovative strategies in the fields of catalyst development and reactor engineering.

Recent studies have reported the high selectivity of *p*‐block metals (such as Sn, Pb, In, and Bi) for CO_2_ electroreduction to formate (HCOO^−^) [16, 17]. Among these, bismuth (Bi) has emerged as a superior candidate due to its low cost, environmental friendliness, high HCOO^−^ FE, and intrinsic ability to suppress the competing hydrogen evolution reaction (HER) [18, 19]. While Bi‐based materials exhibit promising selectivity and cost‐effectiveness, insufficient active site density and suboptimal ^*^OCHO intermediate binding energy substantially constrain their activity. During the past few years, strategies ranging from tuning the electronic structure to morphology control, including non‐metallic element doping [20, 21], hybrid material design [22], grain boundaries [23, 24], confinement effects [25], defect engineering [26], and nano‐structuring [27, 28], have been widely demonstrated to enhance the CO_2_RR performance of Bi‐based catalysts. Although substantial progress has improved catalytic performance, they still face activity‐stability trade‐offs. For example, Bi nanoparticles and nanosheets increase surface area but often suffer from structural instability; dopants such as sulfur or phosphorus can improve conductivity but at the expense of active site availability [22, 29, 30, 31]. To date, Bi‐based catalyst systems still suffer from insufficient activity and stability, as evidenced by suboptimal partial current densities (< 500 mA cm^−2^) for HCOOH production and significant performance degradation under prolonged operation at high current densities. These pose significant barriers to the deployment of industrial‐scale CO_2_‐to‐HCOOH conversion processes, which makes the development of newly advanced Bi‐based catalysts with high activity and stability an urgent demand.

In recent years, introducing strain into catalysts has emerged as a powerful strategy to increase their catalytic activity and stability for diverse electrocatalytic processes, including oxygen reduction/evolution [32, 33] and hydrogen evolution [34]. Tuning the electronic structure of catalytically active sites via strain engineering can modulate the adsorption and desorption energies of reaction intermediates, thereby facilitating catalytic reactions while simultaneously enhancing structural stability [35]. As a result, strain engineering has been deemed a straightforward yet powerful approach to optimize both activity and durability. Although promising, this strategy requires further exploration in the engineering of Bi‐based catalysts to enhance CO_2_‐to‐HCOOH conversion. Another fundamental challenge, besides catalyst design, in CO_2_RR lies in the product‐electrolyte mixing within conventional liquid electrolyte systems, which inevitably generates mixed product streams that require costly separation processes [36, 37, 38]. This inherent limitation exists regardless of the electrolyte pH (acidic, neutral, or alkaline) and severely hinders the practical implementation of CO_2_RR technology. To address this, solid‐state electrolyte (SSE) reactors have recently been developed to replace conventional H‐cells and flow cells by transporting electrogenerated cations or anions between the anode and cathode, enabling the production of pure liquid CO_2_RR products [39, 40, 41, 42, 43, 44]. This reactor design resolves the system‐level challenge of product‐electrolyte mixing, facilitating the production of high‐purity liquid fuels. Therefore, integrating strain‐engineered Bi‐based catalysts with SSE reactors represents a novel and highly promising strategy for producing pure HCOOH solutions via CO_2_RR with high selectivity and stability at high current densities. However, this approach remains unexplored, with no systematic studies to date, marking a critical gap in both fundamental understanding and practical application in CO_2_RR technology.

Herein, we report a tensile‐strained Bi‐based nanosheets (TS‐BiNs) catalyst synthesized via a simple mechanical ball‐milling and carbonation restructuring preparation technique for the electrochemical conversion of CO_2_ to a pure HCOOH solution. The resultant TS‐BiNs catalyst exhibits a marked FE of exceeding 92% for HCOO^−^ production at wide current densities ranging from −50 to −1000 mA cm^−2^ in a flow cell and continuously produces HCOO^−^ at −100 mA cm^−2^ over a course of 100 h. When employed at the cathode of the SSE reactor (4 cm^2^), this catalyst enables continuous production of pure HCOOH solutions with tunable concentrations at a current of 400 mA, delivering energy efficiency of 34% at a flow rate of 20 mL h^−1^. Techno‐economic analysis (TEA) confirms that using TS‐BiNs as the cathode catalyst in the SSE reactor enables the production of HCOOH at a total cost of $218.96 per ton, which represents an approximately 77% reduction compared to the market price of $956 per ton [45], indicating that the process is profitable. In situ studies and density functional theory (DFT) calculations reveal that introducing tensile strain into Bi catalysts modulates the kinetics of the ^*^OCHO intermediate, optimizes its adsorption‐desorption equilibrium, and lowers the energy barrier for ^*^OCHO formation. These effects collectively balance the reaction energy barriers of the critical steps in CO_2_RR while simultaneously suppressing the competing HER, thereby contributing to the catalyst's outstanding performance.

## 2 Results and Discussion

### 2.1 Preparation and Structural Characterization of the TS‐BiNs Catalyst

**FIGURE 1:**
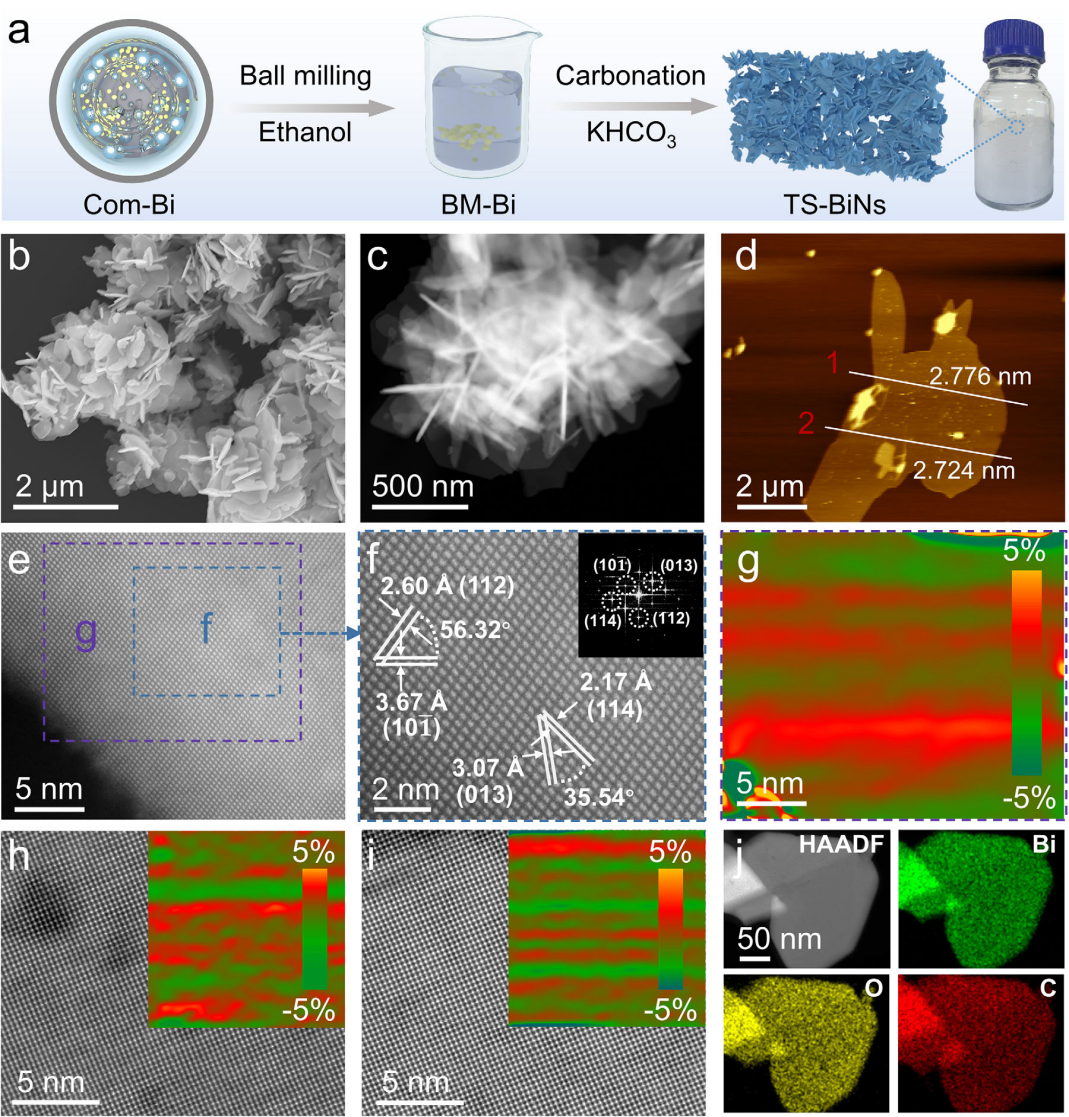
Synthesis and characterization of the TS‐BiNs catalyst. (a) Schematic illustration of the synthesis of TS‐BiNs. (b) SEM image, (c) HAADF‐STEM image, (d) AFM image, and (e) High‐resolution HAADF‐STEM image of TS‐BiNs. (f) The high‐resolution atom image and the inset in (f) correspond to its Fast Fourier Transform pattern (enlarged view of the blue box in (e)). (g) HAADF‐STEM‐GPA analysis of the strain distribution. The red and green areas represent tensile and compressive strains (ε_yy_), respectively (enlarged view of the purple box in (e)). (h, i) HAADF‐STEM images of TS‐BiNs in different regions and the corresponding GPA analysis image. (j) HAADF‐STEM image of TS‐BiNs and the corresponding EDS elemental mapping images.

Commercial Bi_2_O_3_ catalysts (denoted as Com‐Bi) exhibit inherent deficiencies in particle size distribution, active site exposure, and reaction dynamics, collectively compromising their electrocatalytic performance. To address these challenges while leveraging the intrinsic scalability and cost‐effectiveness of the material, we developed a ball‐milling (strain‐engineering achieved in this step) and a reconstructing strategy to transform bulk Bi_2_O_3_ into ultrathin Bi‐based nanosheets with tensile strain. Figure 1a depicts our developed preparation method, through which Com‐Bi precursors were first subjected to mechanochemical ball milling processing in an agate grinding chamber containing ethanol to obtain ball‐milled Bi_2_O_3_ with tensile strain (BM‐Bi) powders. During high‐energy ball milling, repeated high‐frequency collisions and compressive interactions among the grinding balls, Bi_2_O_3_ particles, and the mill chamber not only drive the shear‐induced fragmentation and size refinement of Bi_2_O_3_ particles but also induce significant tensile strain within the crystal lattice. The transient mechanical forces generate localized stress concentrations and abundant surface defects in the lattice, while simultaneously activating the originally inert non‐defective sites [46, 47]. Following ball milling, the BM‐Bi powders were then carbonated to afford tensile‐strained ultrathin Bi‐based nanosheets, i.e., TS‐BiNs. The ball milling time and carbonation time were systematically optimized to determine the optimal preparation conditions (see Section S1for synthesis details).

We investigated the morphological evolution of the Com‐Bi catalyst during strain engineering and reconstruction by scanning electron microscopy (SEM) (Figure S1). SEM images show that carbonation reconstruction in alkaline media converted these nanoparticles into ultrathin nanosheets (Figure 1b; Figure S1c). Energy dispersive X‐ray spectroscopy (EDS) mapping images reveal the uniform incorporation of carbon species into the bismuth matrix during alkaline‐mediated reconstruction, as evidenced by comparative elemental profiling of pristine Com‐Bi and reconstructed TS‐BiNs (Figures S2 and S3), indicating a change in chemical composition [48]. High‐angle angular dark‐field scanning transmission electron microscopy (HAADF‐STEM) images (Figure 1c) and atomic force microscopy (AFM) images (Figure 1d; Figure S4) further confirm that the as‐formed Bi‐based catalyst exhibits an ultrathin nanosheet structure with a thickness of approximately 2.7 nm. In contrast, the nanosheets derived from Com‐Bi without ball milling under carbonation conditions display thicknesses ranging from 3.6 to 10 nm (Figure S5), suggesting that the ball milling process plays a vital role in decreasing the thickness of the as‐obtained nanosheets. We found that uniform nanosheet structures form exclusively at 30 min of ball milling and 30 min of carbonation, with deviations leading to nanosheet morphologies with partial particle aggregation (Figures S6 and S7). Importantly, our scale‐up synthesis experiments revealed that the TS‐BiNs catalyst can be produced at the sub‐kilogram scale (Figure S8). This result demonstrates its great potential for large‐scale industrial use.

We also investigated the atomic‐scale structural evolution induced by mechanochemical and carbonation synthesis strategies using electron microscopy. HAADF‐STEM reveals the ultrathin nanosheet morphology of the TS‐BiNs catalysts, exhibiting an increase in the surface‐to‐volume ratio compared with that of untreated Bi_2_O_3_ (Figure 1e; Figures S9 and S10). The high‐resolution HAADF‐STEM image shows lattice spacings of 2.60 Å (112), 3.67 Å (10$\bar{1}$), 3.07 Å (013), and 2.17 Å (114) (Figure 1f), corresponding to bismuth subcarbonate phases (Bi_2_O_2_CO_3_), suggesting that the prepared TS‐BiNs catalyst consists of Bi_2_O_2_CO_3_ (see our Powder X‐Ray Diffractometer (PXRD) analysis below). Notably, the observed lattice fringes corresponding to the (114) plane suggest a preferred crystal growth orientation along this high‐index direction in Bi_2_O_2_CO_3_ catalysts (Figure 1f; Figures S11 and S12).

To give detailed insight into the unique inherent strain induced by the ball milling process, we performed geometric phase analysis (GPA) on HAADF‐STEM images of the synthesized TS‐BiNs and Com‐Bi after carbonation. As shown in Figure 1g, significant tensile strain is observed across the entire crystal facet of the TS‐BiNs prepared by the ball milling and carbonation reconstruction method. Importantly, GPA analyses of different HAADF‐STEM regions further confirmed the presence of tensile strain (Figure 1h,i; Figure S13). In contrast, the multi‐region HAADF‐STEM and GPA characterizations of Com‐Bi after carbonation reveal that its strain state is near‐zero (Figures S14 and S15). These results confirm that tensile strain can be successfully introduced into Bi‐based catalysts via the ball milling method [49, 50]. STEM‐EDS mapping confirmed the homogeneous distributions of Bi, O, and C across the TS‐BiNs (Figure 1j).

### 2.2 Crystal and Electronic Properties of the Catalysts

**FIGURE 2:**
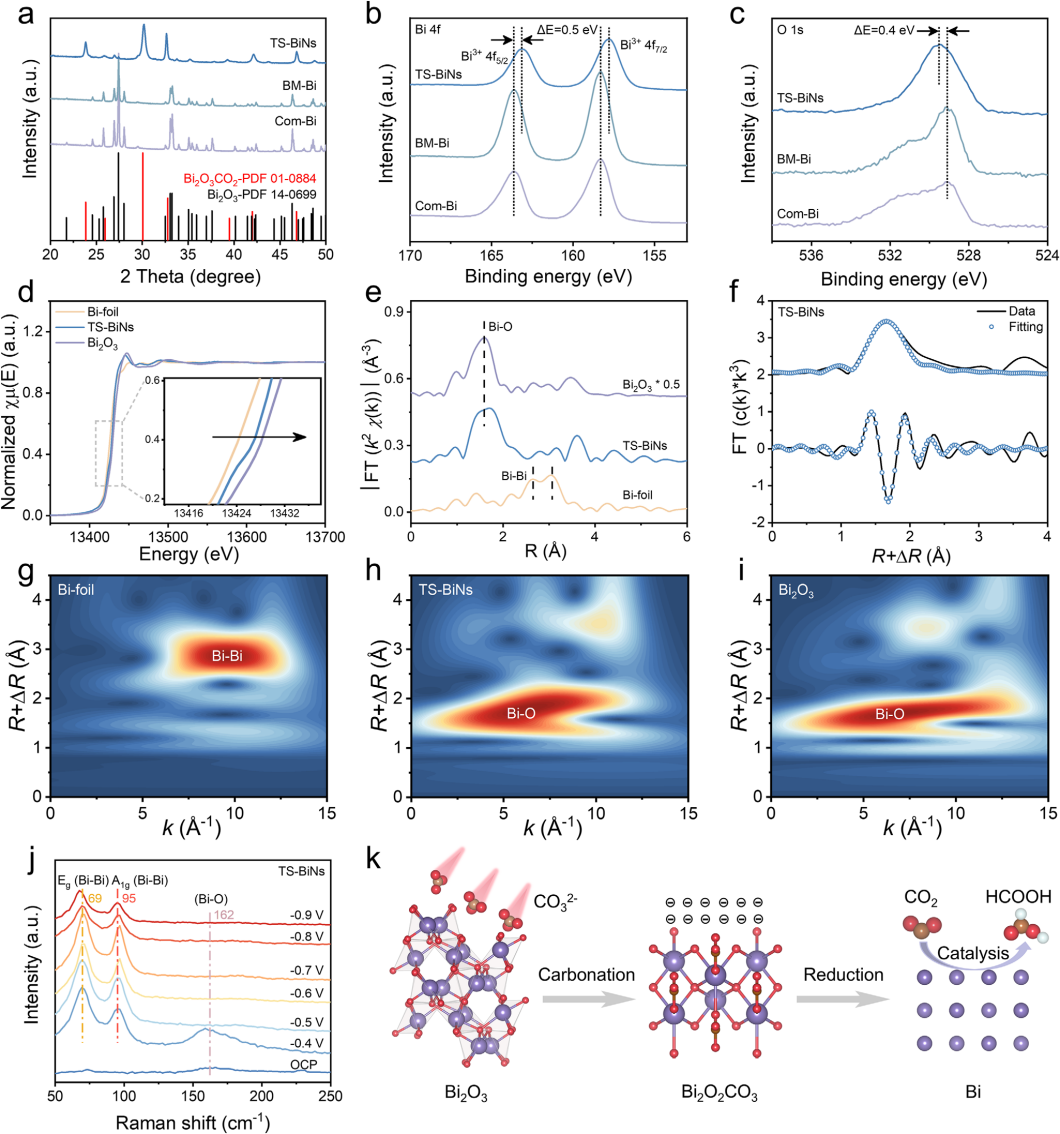
Crystal and electronic structure analysis of the catalysts. (a) PXRD patterns of Com‐Bi, BM‐Bi, and TS‐BiNs. (b, c) XPS high‐resolution spectra of Bi 4f and O 1s of Com‐Bi, BM‐Bi, and TS‐BiNs. (d) XANES spectra at the Bi L_3_‐edge for the TS‐BiNs, Bi‐foil, and Bi_2_O_3_. (e) R‐space EXAFS spectra. (f) Bi L_3_‐edge EXAFS (black lines) and fitting curves (blue points) for TS‐BiNs in R‐space, including the FT magnitude (up) and imaginary component (down). The data are k^3^‐weighted without phase correction. (g–i) Wavelet transforms for the k^3^‐weighted EXAFS signals of Bi‐foil, TS‐BiNs, and Bi_2_O_3_. (j) In situ Raman spectra of the TS‐BiNs catalyst during the CO_2_RR at different applied potentials. (k) Schematic illustration of the evolution of the catalyst for the CO_2_RR in this study.

We used PXRD, X‐ray photoelectron spectroscopy (XPS), and X‐ray absorption fine structure (XAFS) analyses to investigate the crystal and electronic structures of the catalysts. PXRD patterns confirmed that both the Com‐Bi and BM‐Bi catalysts exhibited the crystal structure of Bi_2_O_3_ (PDF 14–0899), without other diffraction peaks (Figure 2a). Notably, the diffraction peaks of BM‐Bi are significantly lower in intensity than those of Com‐Bi, indicating a substantial decrease in particle size after ball milling, while the crystal structure remains unchanged, consistent with SEM observations. After reconstruction in KHCO_3_ solution, BM‐Bi transforms into the Bi_2_O_2_CO_3_ phase, in agreement with high‐resolution HAADF‐STEM results. This finding agrees with the literature reports that in an alkaline environment, Bi_2_O_3_ undergoes hydrolysis to yield Bi species, which then combine with carbonate ions to form basic bismuth carbonate [25, 51]. Notably, the (013) and (110) facet diffraction peaks of TS‐BiNs shift to a lower angle than those of the carbonated Com‐Bi catalyst (Figure S16). This diffraction shift further confirms the presence of tensile strain in TS‐BiNs, consistent with the aforementioned HAADF‐STEM and GPA results.

The XPS survey spectra confirmed the presence of Bi and O in Com‐Bi, and Bi, O, and C in TS‐BiNs (Figure S17). Figure 2b presents the high‐resolution spectra of Bi 4f for Com‐Bi, BM‐Bi, and TS‐BiNs. The peaks at 158.32 and 163.66 eV correspond to Bi 4f_7/2_ and Bi 4f_5/2_, respectively [27, 48]. Compared with those of Com‐Bi and BM‐Bi, the peaks of the TS‐BiNs exhibit a significant negative shift of approximately 0.5 eV. Moreover, the O 1s peak experiences a positive shift of 0.4 eV (Figure 2c). These shifts primarily reflect the successful phase transformation from Bi_2_O_3_ to Bi_2_O_2_CO_3_ and the resulting alteration in the local chemical environment around Bi and O species. Such distinct electronic states in TS‐BiNs are conducive to optimizing the adsorption of intermediates, and promoting the highly selective conversion of CO_2_‐to‐HCOO^−^.

XAFS analysis provides fundamental insights into the Bi valence state and coordination environment of TS‐BiNs [52]. The Bi L_3_‐edge X‐ray absorption near‐edge structure (XANES) spectra reveal that the absorption edge of TS‐BiNs is slightly negatively shifted compared with that of Bi_2_O_3_ (Figure 2d; Figure S18). This observation indicates a decrease in the valence state of Bi in TS‐BiNs compared with Bi_2_O_3_, which is consistent with the above XPS analysis. The Fourier‐transformed extended X‐ray absorption fine structure (FT‐EXAFS) spectra (Figure 2e; Figure S19) show that the prominent peak of the Bi─O scattering path in Bi_2_O_3_ is located at 1.59 Å. For TS‐BiNs, the main peak of the Bi─O scattering path is slightly shifted to 1.72 Å, accompanied by a broadening of the peak shape. These findings indicate that the tensile strain effect and carbonation processes adjust the Bi─O bond length and alter the structure [24]. Similarly, changes are also observed in the Bi─Bi scattering path. The optimal results of EXAFS fitting confirm these alterations (Figure 2f; Figure S20 and Table S1). Specifically, the length of the Bi─O bond has been extended from 2.14 Å for Bi_2_O_3_ to 2.20 Å for TS‐BiNs, suggesting a reduction in the covalency of the Bi─O bond. Then, the first‐shell Bi─O coordination environment in TS‐BiNs is further confirmed by the wavelet transform (WT) analysis of the Bi L_3_‐edge (Figure 2g–i).

We used in situ Raman spectroscopy to identify active sites during CO_2_RR (Figure S21). Under open‐circuit potential (OCP) conditions, a distinct Bi─O (162 cm^−1^) vibrational peak is clearly observed. When the potential was adjusted to −0.4 V versus RHE, Bi─Bi (69 and 95 cm^−1^) and Bi─O signals are observed at the same time (Figure 2j; Figure S22) [53, 54]. With a further decrease in potential, metallic Bi serves as the active site for CO_2_‐to‐HCOO^−^ conversion. Figure 2k illustrates the structural evolution of the Bi‐based catalyst. Specifically, Bi_2_O_3_ undergoes carbonation to form Bi_2_O_2_CO_3_, and subsequently, is reduced to metallic Bi in the CO_2_RR process, acting as the active phase for CO_2_‐to‐HCOOH. Notably, tensile strain persists in the TS‐BiNs pre‐catalyst during the entire CO_2_RR process (see post‐reaction characterizations below).

### 2.3 Electrochemical Reduction of CO_2_‐to‐HCOO^−^

**FIGURE 3:**
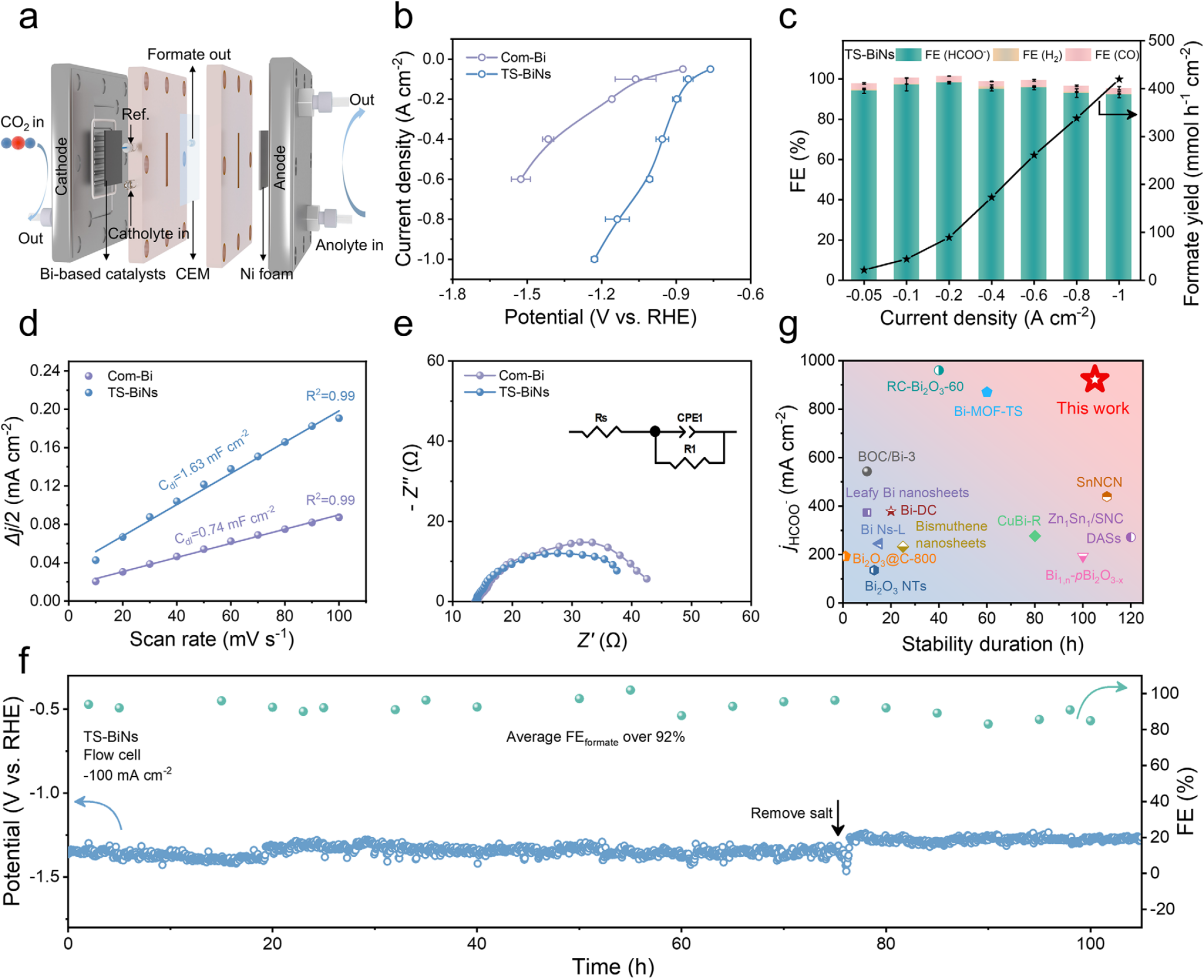
CO_2_ electroreduction performance in a flow cell. (a) Schematic illustration of the flow cell. (b) The I‐V curves of TS‐BiNs and Com‐Bi in a flow cell when using a 1.0 m KHCO_3_ electrolyte (with iR correction). (c) The FE and the formate yields at different current densities over the TS‐BiNs in a flow cell; electrolyte: 1.0 m KHCO_3_, reaction area: 1.0 cm^2^. (d) Plots for calculating the double‐layer capacitance of TS‐BiNs and Com‐Bi. (e) Electrochemical impedance spectra plot at −1.0 V for TS‐BiNs and Com‐Bi. The insert is the equivalent circuit diagram. (f) Stability test at a current density of −100 mA cm^−2^ in a flow cell (without iR correction). (g) Comparison of the performance of TS‐BiNs with that of typical electrocatalysts reported in the literature for the CO_2_RR to HCOO^−^. Note that the maximal partial current density of formate for TS‐BiNs is derived from figure c.

To verify how tensile strain affects CO_2_RR performance, we evaluated the CO_2_RR performance of the catalysts in a gas‐diffusion layer‐based flow cell (Figure 3a; Figure S23). As clearly shown in Figure 3b and Figure S24, the TS‐BiNs catalyst has a higher current density and a lower potential than Com‐Bi, indicating that tensile strain significantly enhances the activity of CO_2_‐to‐HCOO^−^. Notably, the TS‐BiNs reaches a current density of −1000 mA cm^−2^ at −1.23 V, far exceeding the Com‐Bi (−600 mA cm^−2^ at −1.52 V), demonstrating its superior electrocatalytic activity for the CO_2_RR.

Moreover, we studied the CO_2_RR selectivity of TS‐BiNs, Com‐Bi, and BM‐Bi catalysts over a wide range of current densities. As shown in Figure 3c and Figure S25, TS‐BiNs maintain high FE_HCOO_^−^ (> 92%) across the entire current density window from −50 to −1000 mA cm^−2^, achieving a high partial current density (*j*_HCOO−_) of −920 mA cm^−2^ and −1.23 V. In contrast, Com‐Bi reached only 74% FE_HCOO_^−^ at −400 mA cm^−2^ and −1.42 V, and BM‐Bi attained 84.5% FE_HCOO_^−^ at −1000 mA cm^−2^ and −2.13 V (Figures S26–S28). Furthermore, at −1,000 mA cm^−2^, the HCOO^−^ yield of the TS‐BiNs catalyst was as high as 420 mmol h^−1^ cm^−2^, which was much higher than that of Com‐Bi (139 mmol h^−1^ cm^−2^ at −400 mA cm^−2^) and BM‐Bi (394 mmol h^−1^ cm^−2^ at −1000 mA cm^−2^). Further experiments with varying ball‐milling and reconstruction durations confirm that all treated catalysts outperform untreated Com‐Bi, highlighting the crucial role of tensile strain in enhancing CO_2_RR performance (Figure 3c; Figure S29).

The electrochemically active surface areas (ECSA) of the TS‐BiNs and Com‐Bi catalysts were investigated by measuring the double‐layer capacitance (C_dl_) (Figure S30). We found that the C_dl_ of TS‐BiNs (1.63 mF cm^−2^) is 2.2 times greater than that of Com‐Bi (0.74 mF cm^−2^), indicating that TS‐BiNs expose more active sites than Com‐Bi does (Figure 3d). Even after ECSA normalization, TS‐BiNs still exhibit a relatively high intrinsic current density, confirming its superior intrinsic catalytic activity (Figure S31). The electrochemical impedance spectroscopy (EIS) results show that the TS‐BiNs catalyst has a lower charge transfer resistance than Com‐Bi does (Figure 3e), confirming the enhanced efficiency of the charge transfer kinetics in TS‐BiNs, which thereby enables superior electrocatalytic CO_2_RR performance. The catalyst also exhibits exceptional long‐time stability, maintaining an average FE_HCOO_^−^ above 92% over 100 h of continuous operation at −100 mA cm^−2^ without significant potential decay (Figure 3f; Figure S32). Its performance in activity, selectivity, and stability at industrial current densities surpasses most previously reported Bi‐based catalysts, highlighting its practical potential (Figure 3g; Table S2).

To demonstrate the excellent stability of the TS‐BiNs catalyst as well as the important role of the tensile strain of TS‐BiNs in enhancing performance, we also carefully characterized the post‐catalysis materials. The SEM images show that the post‐catalysis TS‐BiNs catalyst still retains the microstructure of the nanosheets after the stability test (Figure S33). Post‐reaction XRD analysis reveals that the catalyst undergoes a phase transformation to metallic Bi after CO_2_RR (Figure S34). Notably, the diffraction peaks of the formed metallic Bi in TS‐BiNs‐CO_2_RR shift toward lower angles compared to those in Com‐Bi‐CO_2_RR. This shift indicates a sustained lattice expansion, suggesting that the metallic Bi derived from TS‐BiNs consistently preserves tensile strain throughout the CO_2_RR process. We further analyzed the atomic structure of the catalysts after the reaction using HAADF‐STEM. The HAADF‐STEM images of the Com‐Bi catalyst after the CO_2_RR reveal lattice spacings of 2.35 and 3.28 Å, corresponding to the (104) and (012) facets of rhombohedral Bi, respectively (Figure S35). These lattice spacings are well consistent with the theoretical values of rhombohedral Bi, indicating that no strain was experienced for the Com‐Bi catalyst during the CO_2_RR process. In contrast, the HAADF‐STEM images of the TS‐BiNs catalyst after the CO_2_RR process show a lattice spacing of 3.41 Å, which can be assigned to the (012) facet of rhombohedral Bi. The value is 0.13 Å larger than the standard value of 3.28 Å, indicating a lattice expansion rate of approximately 4% (Figure S36). The results of XRD and HAADF‐STEM analyses provide strong evidence that tensile strain persists in the TS‐BiNs nanosheets throughout the CO_2_RR process.

### 2.4 Production of Pure HCOOH Solutions

**FIGURE 4:**
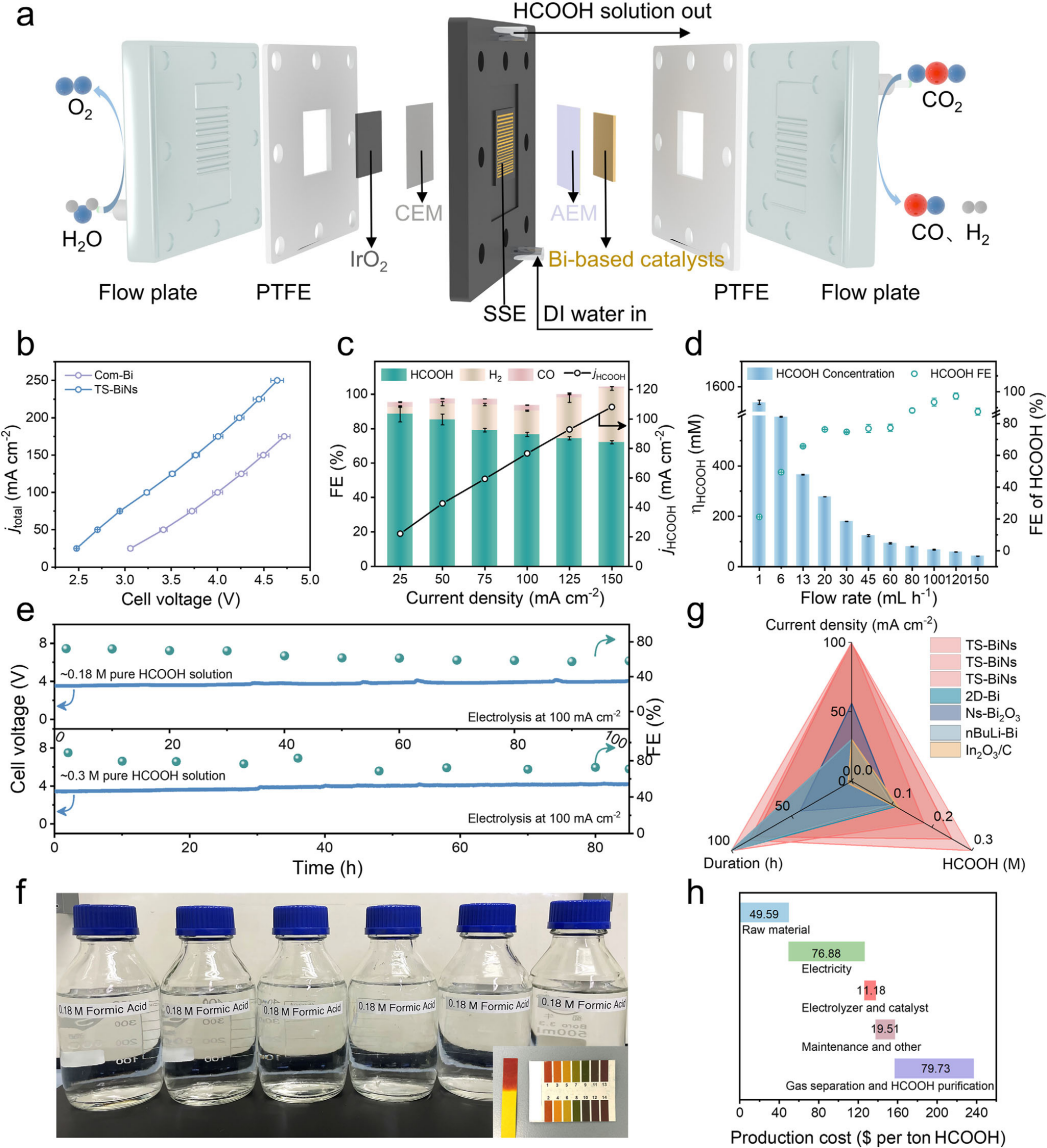
Electrosynthesis of pure HCOOH solutions in a solid‐state electrochemical CO_2_RR reactor (4 cm^2^). (a) Schematic illustration of the CO_2_RR in the SSE reactor for continuous production of a pure HCOOH solution. (b) The *I*–*V* curves of the TS‐BiNs and Com‐Bi catalysts (without iR correction). (c) FE and partial current density of HCOOH at different current densities for the TS‐BiNs catalyst (flow rate at 15 mL h^−1^). (d) Dependence of the concentration of the HCOOH product on the flow rate under a fixed overall current of 400 mA. (e) Long‐time stability test and the corresponding FE of TS‐BiNs for continuous CO_2_RR to HCOOH solution at 400 mA (flow rates of 30 mL h^−1^ (up) and 20 mL h^−1^ (down)) (without iR correction). (f) Photographs of 3 L of 0.18 m formic acid solution (pH 2.2) obtained from long‐time electrolysis. (g) Comparison of the electrochemical CO_2_RR and HCOOH performance of TS‐BiNs with those of recently reported novel catalysts. For more detailed information, please refer to Table S3. (h) The subdivided cost of the entire process for HCOOH electrosynthesis from CO_2_.

Given the inherent issue of product‐electrolyte mixing in the conventional flow cell, we demonstrated that in the SSE reactor configuration, TS‐BiNs catalysts can efficiently convert CO_2_ to pure HCOOH, circumventing complex product separation and purification processes and eliminating concerns about salt accumulation. Figure 4a and Figures S37 and S38 show the schematic reaction mechanism, photographs of the SSE reactor, and the middle layer. Electrochemical investigations revealed that the TS‐BiNs catalyst exhibited much lower cell voltages than did the pristine Com‐Bi catalysts at identical current densities (Figure S39). This result underscores the superior ability of TS‐BiNs to convert CO_2_ to HCOOH, which is consistent with the flow cell results. Notably, at a cell voltage of 4.23 V (without iR correction), the TS‐BiNs catalyst can exceed 200 mA cm^−2^, whereas Com‐Bi reached only 125 mA cm^−2^ even above 4.25 V (Figure 4b). The TS‐BiNs catalyst maintained a high FE for HCOOH solution across a wide range of current densities at a flow rate of 15 mL h^−1^. Specifically, the highest FE reaches 88% at 25 mA cm^−2^. At 150 mA cm^−2^, an FE of 72% for HCOOH is achieved, with a corresponding HCOOH partial current density of 108 mA cm^−2^ (Figure 4c; Figure S40). Nuclear magnetic resonance (NMR) spectra unequivocally confirmed that HCOOH was the sole liquid phase product (Figure S41). The relationship between energy efficiency (EE) and current density reveals that TS‐BiNs achieve a maximum EE of 51% at 25 mA cm^−2^. Furthermore, the system maintains an EE of 34% at a higher current density of 100 mA cm^−2^ (Figure S42).

One prominent advantage of the SSE reactor is the precise control of the concentration of pure HCOOH solution by modulating the flow rate of deionized water (Figure 4d) [37]. By decreasing the deionized water flow rate (1 mL h^−1^), a high HCOOH concentration of 1538 mm (approximately 7.1 wt.%) can be achieved during CO_2_‐to‐HCOOH conversion at a high current of 400 mA. At a flow rate of 120 mL h^−1^, the FE of HCOOH approaches 97%. The decrease in FE at elevated concentrations stems from thermodynamic suppression of CO_2_ conversion and potential HCOOH crossover through the Nafion membrane during anode oxidation, a phenomenon analogous to direct formic acid fuel cells [15, 55, 56]. Remarkably, the developed system also demonstrated exceptional stability during continuous CO_2_RR. At a fixed current of 400 mA and a deionized water flow rate of 30 mL h^−1^, the system remained operational for 100 h, producing approximately 3 L of 0.18 m pure HCOOH solution (> 60% FE) with negligible change in the cell voltage (Figure 4e,f; Figure S43). The measured pH of 2.19 for the pure HCOOH solution was in excellent agreement with the theoretical value of 2.2 using a pH meter (Figure S44). Moreover, the system was validated to stably produce different concentrations of HCOOH solution at various flow rates (Figure 4e; Figure S45). When the deionized water flow rate in the middle layer was reduced to 25 and 20 mL h^−1^, pure HCOOH solution with concentrations of approximately 0.25 m (> 60% FE) and 0.3 m (> 70% FE) was obtained at the same current of 400 mA. Moreover, the system maintained stable operation for over 80 h, during which no significant voltage fluctuations were observed. The excellent stability of the TS‐BiNs catalyst should be attributed to the strain‐induced lattice tension and the formation of nanosheet structures, which help prevent the aggregation and deactivation of active sites during long‐time operations. Importantly, TS‐BiNs exhibit remarkable advantages over recently reported state‐of‐the‐art catalysts in key performance indicators for CO_2_ reduction to pure HCOOH (Figure 4g; Table S3). Not only do they outperform existing catalysts in operable current density and stability duration, but they also demonstrate excellent performance in the production of pure HCOOH at different concentrations. Additionally, we conducted a TEA to assess the economic viability of this process using methodologies established in previous reports [5, 39, 55] (Note S1). According to performance data of the SSE reactor for CO_2_‐to‐HCOOH conversion, the estimated production cost is approximately $236.89 per ton of HCOOH, significantly lower than the current market price of $956 per ton, demonstrating very strong economic competitiveness (Figure 4h; Table S4). The cost breakdown includes raw materials, electricity (32.45%), electrolyzer, catalyst, maintenance, and other expenses. With the development of renewable energy sources, electricity costs are expected to decrease further. Additionally, recycling and using the high‐purity oxygen generated at the anode could reduce the cost by an additional $17.93 per ton. As a result, the actual total cost of this process can be reduced to around $218.96 per ton, representing a 77% reduction compared to the current market price.

### 2.5 In Situ Mechanism Studies

**FIGURE 5:**
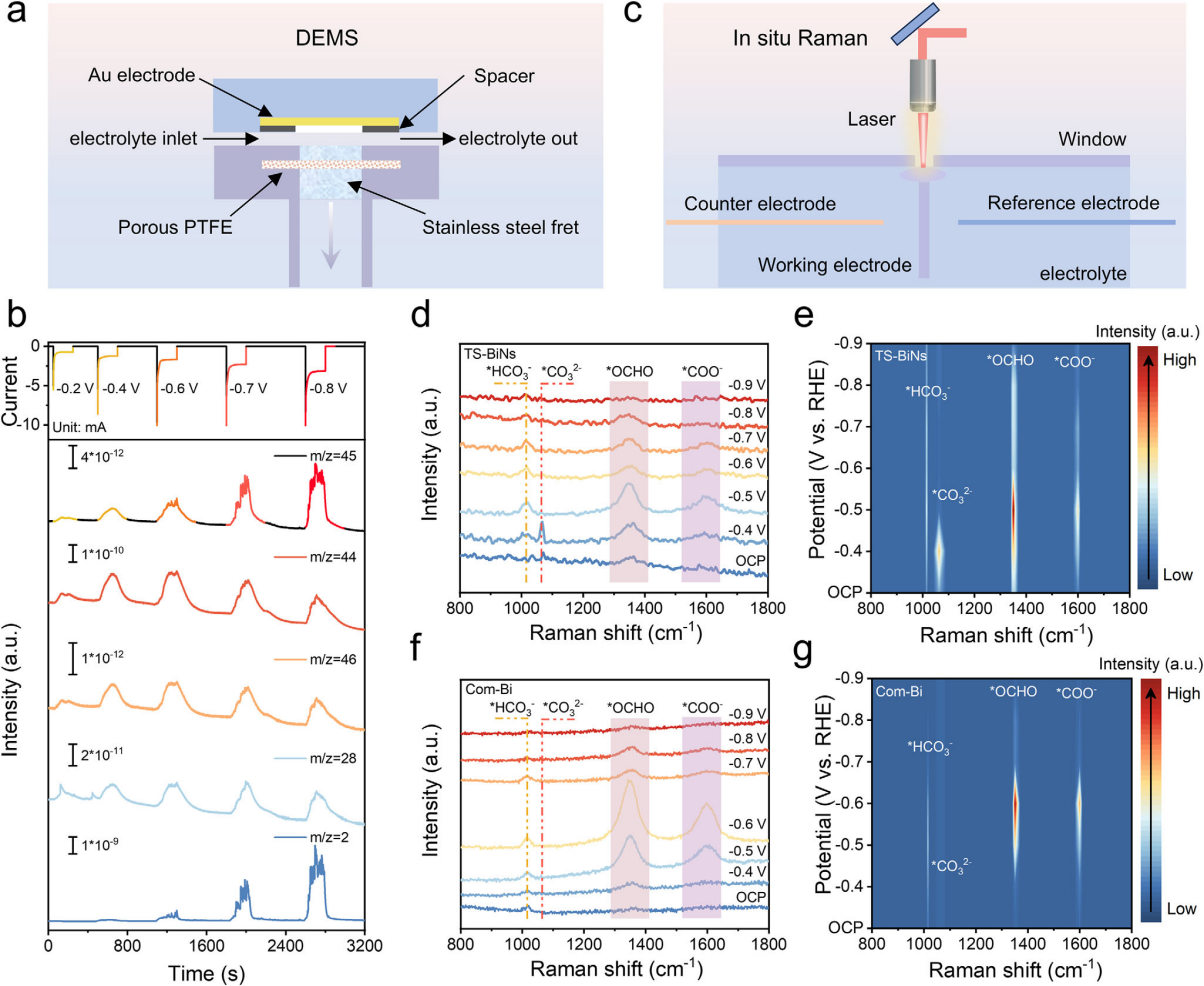
In situ electrocatalytic mechanism studies. (a) Schematic diagram of the electrochemical online DEMS test. (b) Multi‐potential steps curves and DEMS ion current signals over the TS‐BiNs catalyst in a 1.0 m KHCO_3_ electrolyte. (c) Schematic depiction of the in situ Raman spectroscopy test. In situ Raman spectra and the corresponding contour images for (d, e) TS‐BiNs and (f, g) Com‐Bi at different potentials.

We systematically analyzed the electroreduction reaction pathway and the kinetic behavior of intermediates over the TS‐BiNs catalyst in the CO_2_RR process using online differential electrochemical mass spectrometry (DEMS) and in situ electrochemical Raman spectroscopy. To identify the reaction intermediate and onset potential of TS‐BiNs during the CO_2_RR, DEMS was employed to monitor key mass‐to‐charge ratio (m/z) signals in real time (Figure 5a; Figure S46). During the chronoamperometry tests, the synchronous evolution of m/z = 2 (H_2_), 28 (CO), 44 (a decomposition product of CO_2_/HCOOH), 45 (HCOO^−^), and 46 (HCOOH) signals was observed (Figure 5b; Figure S47) [22, 57]. Multi‐potential step analysis revealed a voltage‐dependent increase in the m/z = 45 signal intensity, which unambiguously corresponds to the ^*^OCHO intermediate, thereby providing direct evidence for both the formic acid production pathway and the persistent presence of this key intermediate during the CO_2_RR. As shown in Figure 5b, when the potential reaches −0.2 V, the m/z = 45 signal significantly fluctuates, whereas the m/z = 2 signal is barely detectable, indicating the initiation of HCOO^−^ generation. This confirms a low onset potential and high selectivity for HCOO^−^ production on TS‐BiNs.

In situ Raman spectroscopy uncovered the potential‐dependent evolution of intermediates (Figure 5c; Figure S21). From OCP to −0.5 V, the peak at 1350 cm^−1^ (attributed to the O─C─O vibration of the ^*^OCHO intermediate) increased significantly, whereas the adsorption peak of CO_3_^2−^ at 1064 cm^−1^ weakened from −0.4 to −0.5 V, indicating that the CO_2_ activation step inclined toward the ^*^OCHO intermediate pathway (Figure 5d,e) [51, 58, 59]. TS‐BiNs achieved maximum adsorption at −0.5 V. Notably, when the potential shifted negatively to −0.6 V, the intensity of the ^*^OCHO vibration peak began to decline, suggesting an acceleration of the intermediate desorption process. In contrast, the Com‐Bi catalyst exhibited an ^*^OCHO coverage peak integral area at −0.6 V that was 3.36 times larger than that of TS‐BiNs at −0.5 V, indicating excessive adsorption that hinders subsequent desorption (Figure 5f,g; Figure S48). The tensile strain in TS‐BiNs optimizes intermediate adsorption‐desorption kinetics, enabling high performance without mass transfer limitations or rapid activity loss at high current densities.

### 2.6 DFT Calculations

**FIGURE 6:**
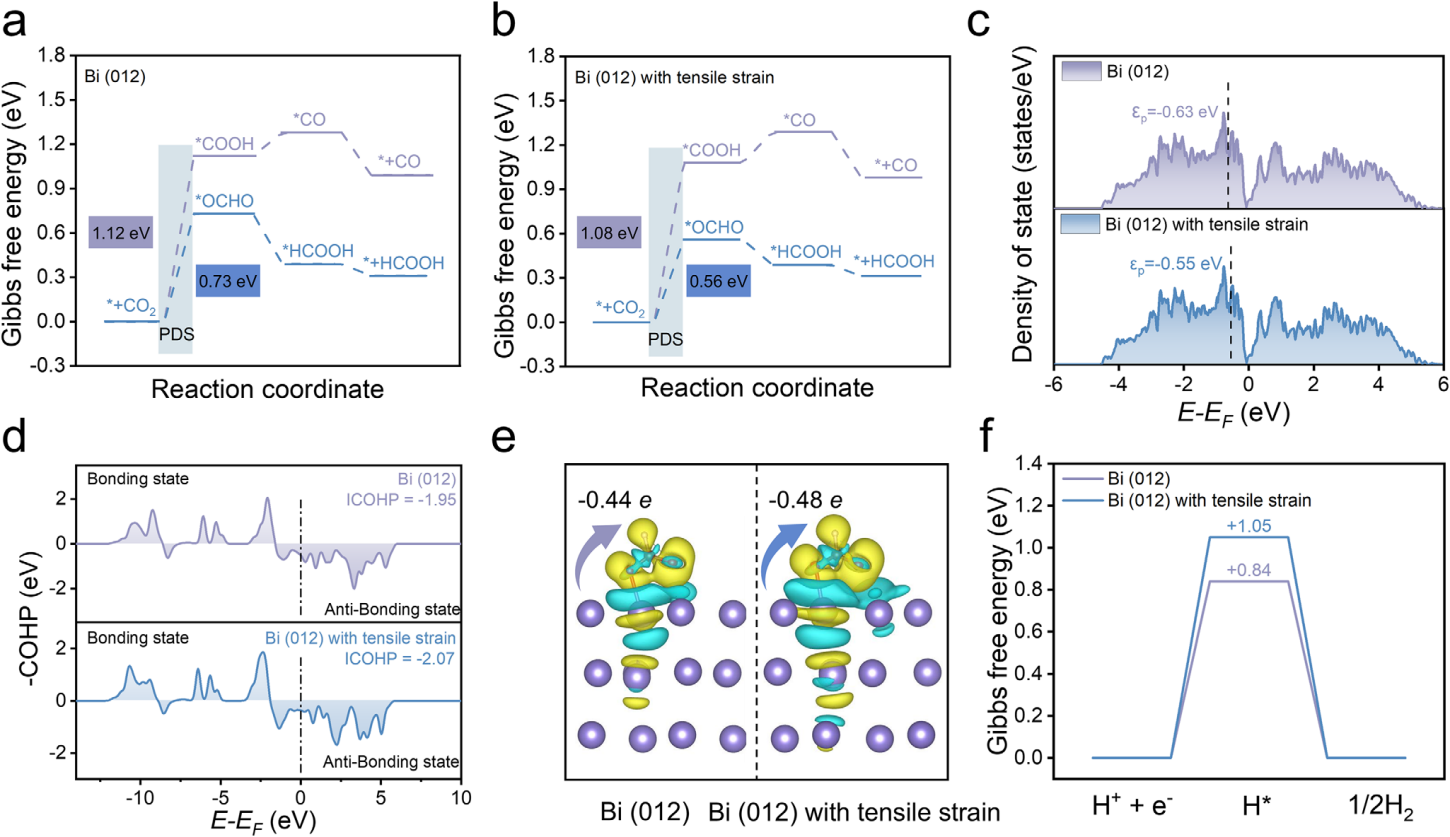
DFT calculations. Gibbs free energy diagrams for CO_2_RR to HCOOH and CO on (a) Bi (012) and (b) Bi (012) with tensile strain models. (c) The p band center of index44 Bi atom for Bi (012) and Bi (012) with tensile strain models, where the index44 Bi atom is the active site for ^*^OCHO. (d) Crystal orbital hamilton population between index44 Bi and O of ^*^OCHO on Bi (012) and Bi (012) with tensile strain models. (e) The charge density difference of OCHO^*^ on Bi (012) and Bi (012) with tensile strain models, the yellow and cyan areas represent electron accumulation and depletion, respectively. (f) Gibbs free energy diagrams for the HER on the Bi (012) and Bi (012) with tensile strain models.

To elucidate the role of tensile strain in enhancing CO_2_‐to‐HCOOH conversion on bismuth catalysts, we performed density functional theory (DFT) calculations using a four‐layer Bi (012) model based on experimental structural data (Figures S33–S36). In the actual CO_2_RR process, the competing intermediates ^*^OCHO and ^*^COOH correspond to the reaction pathways for the conversion of CO_2_‐to‐HCOOH and CO, respectively [60]. Figure 6a,b display the detailed Gibbs free energy diagrams for the CO_2_RR on Bi (012) and Bi (012) with the tensile strain model, and the corresponding structural model is presented in Figures S49–S52. CO_2_ coupled with protons and electrons can form ^*^COOH, where H is bound to the O of the CO_2_ molecule, or ^*^OCHO, where H is bound to the C of the CO_2_ molecule. The formation of ^*^COOH is an endergonic process with Δ*G* of 1.12 eV on Bi (012) and 1.08 eV on Bi (012) with tensile strain, which is significantly greater than ^*^CO formation/desorption, thus serving as the rate‐determining step (RDS) of CO_2_ to CO conversion. The formation of ^*^OCHO on Bi (012) is an uphill process requiring an energy input of 0.73 eV, which is much higher than that of Bi (012) with a tensile strain (0.56 eV). The subsequent desorption process occurs spontaneously, indicating that tensile strain engineering favours the adsorption of ^*^OCHO while lowering the reaction energy barrier, thereby enhancing the selectivity for HCOO^−^. In addition, the Δ*G* of ^*^OCHO formation on both Bi (012) and Bi (012) with tensile strain is substantially lower than that of ^*^COOH formation, indicating that the first protonation of CO_2_ is more energetically favored along the ^*^OCHO path and preferential HCOOH production over CO formation. This result is consistent with previous literature on formic acid selectivity over Bi [19, 28, 50]. In short, Bi (012) is more likely to produce HCOOH compared to CO, and the strain engineering effectively reduces the Δ*G* of ^*^OCHO formation, thereby enhancing the activity of CO_2_‐to‐HCOOH conversion. The above results can be well confirmed by experiments, with only a small amount of CO detected in the experiments.

To probe the tensile strain‐induced enhancement in intermediate adsorption capacity, we calculated the p‐band center (ε_p_). A more positive ε_p_ correlates with stronger adsorption capacity. Figure S53 displays the ε_p_ of Bi atoms on Bi (012) and Bi (012) with tensile strain. The applied strain shifted the ε_p_ from −0.64 to −0.51 eV, which theoretically favors the stabilization of key reaction intermediates. Since ^*^OCHO intermediate bonds with the specific Bi atom at the active site (denoted as index44 in our structural model), we calculated the p‐band center of the index44 Bi atom (Figure 6c). The results demonstrate that the p‐band center of the index44 Bi atom with strain is closer to the Fermi level than the unstrained atom. This essentially explains the strengthened adsorption of CO_2_‐related intermediates.

In addition, we calculated the integrated crystal orbital Hamilton population (ICOHP), a quantitative metric of chemical bond strength. The more negative the ICOHP value, the stronger the bond strength between atoms is. Figure 6d shows that the O─Bi bond of Bi (012) with tensile strain exhibits more negative ICOHP values (−2.07 eV) compared to Bi (012) (−1.95 eV), accounting for its lower Δ*G* value of OCHO formation. Furthermore, we analyzed the charge density difference for ^*^OCHO with the Bi (012) and Bi (012) with tensile strain. The larger electron cloud corresponds to enhanced electron transfer between the catalysts and the ^*^OCHO intermediate. Bi (012) with tensile strain possesses the larger electron cloud, indicating a stronger interaction with the ^*^OCHO intermediate. This is quantitatively verified by Bader charge analysis, which revealed that index44 Bi of Bi (012) with tensile strain loses more electrons (0.48 *e*) to ^*^OCHO compared to index44 of Bi (012) (0.44 *e*) (Figure 6e). Furthermore, HER, as a side reaction, needs to be studied to reflect the real catalytic performance. The Gibbs free energy diagram of the HER shows that the Bi (012) with tensile strain displays weak adsorption of ^*^H with a significantly positive Δ*G* of 1.05 eV (Figure 6f), indicating effective suppression of HER, which is well consistent with much lower H_2_ FE of TS‐BiNs for CO_2_RR tests in a flow cell. The relevant configurations are shown in Figure S54. In summary, introducing tensile strain onto the Bi (012) can strengthen the adsorption of key intermediate (^*^OCHO) and lower the Δ*G* of the PDS, thereby boosting CO_2_RR activity. Concurrently weakening H binding suppresses HER and achieves high selectivity for HCOOH production. These findings align with and clarify experimental observations of the enhanced CO_2_ reduction performance of Bi catalyst with tensile strain, that is, TS‐BiNs.

## 3 Conclusion

We successfully synthesized a scalable TS‐BiNs catalyst through ball milling and carbonization restructuring of commercial Bi_2_O_3_, overcoming the performance bottleneck in CO_2_ electrocatalysis and enabling efficient production of high‐concentration pure HCOOH solutions in the SSE reactor. The combination of the electronic modulation from tensile strain and high‐density edge sites from the nanosheet morphology endows the catalyst with outstanding CO_2_‐to‐HCOOH conversion capability. The TS‐BiNs catalyst exhibits exceptional performance, achieving over 92% FE_HCOO_^−^ at −1000 mA cm^−2^ and enabling continuous production of high‐purity HCOOH solutions (0.18–0.3 m) over 100 h at an industrially relevant current (400 mA) in SSE reactors. TEA analysis confirms that this process can reduce costs to $218.96 per ton HCOOH, representing a 77% reduction compared to market prices. In situ studies and DFT calculations revealed that lattice expansion tensile strain decreases the Gibbs free energy for the formation of the ^*^OCHO intermediate and reduces its activation barrier. This tensile strain simultaneously maintains high energy barriers against competing pathways, leading to CO and H_2_ production, ensuring high HCOOH selectivity. This study effectively bridges the gap between fundamental catalyst/reactor design and industrial CO_2_ utilization, significantly contributing to the development of sustainable electrochemical manufacturing technologies.

## Funding

This work was supported by the National Key R&D Programs of China (2024YFB4105700), the National Natural Science Foundation of China (22579075), the NSFC Excellent Young Scientists Fund (overseas), the startup research funding of SUSTech, Guangdong Provincial Key Laboratory of Sustainable Biomimetic Materials and Green Energy (2024B1212010003), High level of special fund (G03050K002), Shenzhen Basic Research Project (JCYJ20250604144555073), State Key Laboratory of Quantum Functional Materials (K251490701), and the Natural Science Foundation of Sichuan Province (2025NSFJQ0017).

## Conflicts of Interest

The authors declare no conflicts of interest.

## Supporting information




**Supporting File**: advs75152‐sup‐0001‐SuppMat.docx

## Data Availability

The data that support the findings of this study are available from the corresponding author upon reasonable request.

## References

[1] S. Hao , A. Elgazzar , S.‐K. Zhang , et al., “Acid‐Humidified CO_2_ Gas Input For Stable Electrochemical CO_2_ Reduction Reaction,” Science 388 (2025): adr3834, 10.1126/science.adr3834. 40504913

[2] S. Gao , Y. Lin , X. Jiao , et al., “Partially Oxidized Atomic Cobalt Layers For Carbon Dioxide Electroreduction To Liquid Fuel,” Nature 529 (2016): 68–71, 10.1038/nature16455. 26738592

[3] C. Chen , J. F. Khosrowabadi Kotyk , and S. W. Sheehan , “Progress Toward Commercial Application of Electrochemical Carbon Dioxide Reduction,” Chemistry 4 (2018): 2571–2586, 10.1016/j.chempr.2018.08.019.

[4] Y. Y. Birdja , E. Pérez‐Gallent , M. C. Figueiredo , A. J. Göttle , F. Calle‐Vallejo , and M. T. M. Koper , “Advances and Challenges In Understanding The Electrocatalytic Conversion Of Carbon Dioxide To Fuels,” Nature Energy 4 (2019): 732–745, 10.1038/s41560-019-0450-y.

[5] W. Fang , W. Guo , R. Lu , et al., “Durable CO_2_ Conversion In The Proton‐Exchange Membrane System,” Nature 626 (2024): 86–91, 10.1038/s41586-023-06917-5. 38297172

[6] F. Li , A. Thevenon , A. Rosas‐Hernández , et al., “Molecular Tuning of CO_2_‐to‐Ethylene Conversion,” Nature 577 (2020): 509–513, 10.1038/s41586-019-1782-2. 31747679

[7] W. Xia , Y. Xie , S. Jia , et al., “Adjacent Copper Single Atoms Promote C–C Coupling in Electrochemical CO_2_ Reduction for the Efficient Conversion of Ethanol,” Journal of the American Chemical Society 145 (2023): 17253–17264, 10.1021/jacs.3c04612. 37498730

[8] S. Cheon , J. Li , and H. Wang , “In Situ Generated CO Enables High‐Current CO_2_ Reduction to Methanol in a Molecular Catalyst Layer,” Journal of the American Chemical Society 146 (2024): 16348–16354, 10.1021/jacs.4c05961. 38806413

[9] C. Chen , X. Yan , S. Liu , et al., “Highly Efficient Electroreduction of CO_2_ to C^2+^ Alcohols on Heterogeneous Dual Active Sites,” Angewandte Chemie International Edition 59 (2020): 16459–16464, 10.1002/anie.202006847. 32533630

[10] M. Jiang , H. Wang , M. Zhu , et al., “Review on Strategies For Improving The Added Value And Expanding The Scope of CO_2_ Electroreduction Products,” Chemical Society Reviews 53 (2024): 5149–5189, 10.1039/D3CS00857F. 38566609

[11] N. Ye , K. Wang , Y. Tan , et al., “Industrial‐Level CO_2_ To Formate Conversion On Turing‐Structured Electrocatalysts,” Nature Synthesis 4 (2025): 799–807, 10.1038/s44160-025-00769-9.

[12] K. Peramaiah , M. Yi , I. Dutta , et al., “Catalyst Design and Engineering for CO_2_‐to‐Formic Acid Electrosynthesis for a Low‐Carbon Economy,” Advanced Materials 36 (2024): 2404980, 10.1002/adma.202404980. 39394824

[13] P. Zhu and H. Wang , “High‐Purity And High‐Concentration Liquid Fuels Through CO_2_ Electroreduction,” Nature Catalysis 4 (2021): 943–951, 10.1038/s41929-021-00694-y.

[14] Q. Gong , P. Ding , M. Xu , et al., “Structural Defects On Converted Bismuth Oxide Nanotubes Enable Highly Active Electrocatalysis Of Carbon Dioxide Reduction,” Nature Communications 10 (2019): 2807, 10.1038/s41467-019-10819-4. PMC6594929 31243275

[15] C. Zhang , X. Hao , J. Wang , et al., “Concentrated Formic Acid From CO_2_ Electrolysis for Directly Driving Fuel Cell,” Angewandte Chemie International Edition 63 (2024): 202317628, 10.1002/anie.202317628. 38305482

[16] F. Chen , Z.‐C. Yao , Z.‐H. Lyu , J. Fu , X. Zhang , and J.‐S. Hu , “Recent Advances in P‐Block Metal Chalcogenide Electrocatalysts For High‐Efficiency CO_2_ Reduction,” eScience 4 (2024): 100172, 10.1016/j.esci.2023.100172.

[17] P. Li , F. Yang , J. Li , et al., “Nanoscale Engineering of P‐Block Metal‐Based Catalysts Toward Industrial‐Scale Electrochemical Reduction of CO_2_ ,” Advanced Energy Materials 13 (2023): 2301597, 10.1002/aenm.202301597.

[18] Z. Cheng , Z. Yao , S. Kong , C. Qiu , A. B. Wong , and J. Wang , “Advance in Novel Device Design and Microenvironment Modulation for Bismuth‐Based CO_2_ ‐to‐ Formic Acid Electrocatalysis,” Advanced Energy Materials 15 (2025): 02767, 10.1002/aenm.202502767.

[19] Z. Chen , Y. Xiao , X. Qiao , et al., “Monitoring Chalcogenide Ions–Guided In Situ Transform Active Sites Of Tailored Bismuth Electrocatalysts for CO_2_ Reduction To Formate,” Proceedings of the National Academy of Sciences 122 (2025): 2420922122, 10.1073/pnas.2420922122. PMC11912470 40042908

[20] Z. Jiang , S. Ren , X. Cao , et al., “pH‐Universal Electrocatalytic CO_2_ Reduction With Ampere‐Level Current Density on Doping‐Engineered Bismuth Sulfide,” Angewandte Chemie International Edition 63 (2024): 202408412, 10.1002/anie.202408412. 38801019

[21] S. Yang , H. An , S. Arnouts , et al., “Halide‐Guided Active Site Exposure In Bismuth Electrocatalysts For Selective CO_2_ Conversion Into Formic Acid,” Nature Catalysis 6 (2023): 796–806, 10.1038/s41929-023-01008-0.

[22] W. Guo , X. Cao , D. Tan , B. Wulan , J. Ma , and J. Zhang , “Thermal‐Driven Dispersion of Bismuth Nanoparticles Among Carbon Matrix for Efficient Carbon Dioxide Reduction,” Angewandte Chemie International Edition 63 (2024): 202401333, 10.1002/anie.202401333. 38670936

[23] T. Gao , H. Huang , F. Zhang , et al., “Cation Exchange‐Driven Grain Boundary‐Rich Nanorings as Efficient CO_2_ Reduction Electrocatalysts,” Angewandte Chemie International Edition 64 (2025): 202510973, 10.1002/anie.202510973. 40697023

[24] M. Zhang , W. Zhu , Z. Liu , et al., “Selective Sieving Effect of Multi‐Atomic Bismuth Interfaces for Efficient Formate Electrosynthesis and Evolution at Industrial Current Density,” Angewandte Chemie International Edition 64 (2025): 202510206, 10.1002/anie.202510206. 40634240

[25] L.‐P. Chi , Z.‐Z. Niu , Y.‐C. Zhang , et al., “Efficient and Stable Acidic CO_2_ Electrolysis To Formic Acid By A Reservoir Structure Design,” Proceedings of the National Academy of Sciences 120 (2023): 2312876120, 10.1073/pnas.2312876120. PMC10742388 38085783

[26] X. Wang , Y. Zhang , S. Wang , et al., “Steering Geometric Reconstruction of Bismuth With Accelerated Dynamics for CO_2_ Electroreduction,” Angewandte Chemie International Edition 63 (2024): 202407665, 10.1002/anie.202407665. 38837634

[27] X. Cao , Y. Tian , J. Ma , W. Guo , W. Cai , and J. Zhang , “Strong p‐d Orbital Hybridization on Bismuth Nanosheets for High Performing CO_2_ Electroreduction,” Advanced Materials 36 (2024): 2309648, 10.1002/adma.202309648. 38009597

[28] R. Nankya , Y. Xu , A. Elgazzar , et al., “Cobalt‐Doped Bismuth Nanosheet Catalyst for Enhanced Electrochemical CO_2_ Reduction to Electrolyte‐Free Formic Acid,” Angewandte Chemie International Edition 63 (2024): 202403671, 10.1002/anie.202403671. 38887161

[29] W. Li , C. Yu , X. Tan , et al., “Beyond Leverage in Activity and Stability Toward CO_2_ Electroreduction to Formate Over a Bismuth Catalyst,” ACS Catalysis 14 (2024): 8050–8061, 10.1021/acscatal.4c01519.

[30] M. Chen , S. Wan , L. Zhong , et al., “Dynamic Restructuring of Cu‐Doped SnS_2_ Nanoflowers for Highly Selective Electrochemical CO_2_ Reduction to Formate,” Angewandte Chemie International Edition 60 (2021): 26233–26237, 10.1002/anie.202111905. 34586693

[31] W. Zhang , Q. Mao , J. Ding , et al., “S‐doped Ag–Sn Alloy Hollow Microbox for High‐Performance CO_2_ Electroreduction to Formate,” Angewandte Chemie International Edition 64 (2025): 202510743, 10.1002/anie.202510743. 40588432

[32] X. Cao , L. Miao , W. Jia , et al., “Strain Heterogeneity in RuO_2_ for Efficient Acidic Oxygen Evolution Reaction In Proton Exchange Membrane Water Electrolysis,” Nature Communications 16 (2025): 6217, 10.1038/s41467-025-58570-3. PMC12228735 40617809

[33] M. Luo and S. Guo , “Strain‐Controlled Electrocatalysis On Multimetallic Nanomaterials,” Nature Reviews Materials 2 (2017): 17059.

[34] Z. Li , Y. Wang , H. Liu , et al., “Electroreduction‐Driven Distorted Nanotwins Activate Pure Cu for Efficient Hydrogen Evolution,” Nature Materials 24 (2025): 424–432, 10.1038/s41563-024-02098-2. 39900738

[35] S. Zhang , W. Ruan , and J. Guan , “Strain Effects In Carbon Dioxide Electroreduction,” Advanced Energy Materials 14 (2024): 2404057.

[36] T. Zheng , C. Liu , C. Guo , et al., “Copper‐Catalysed Exclusive CO_2_ to Pure Formic Acid Conversion Via Single‐Atom Alloying,” Nature Nanotechnology 16 (2021): 1386–1393, 10.1038/s41565-021-00974-5. 34531557

[37] L. Fan , C. Xia , P. Zhu , Y. Lu , and H. Wang , “Electrochemical CO_2_ Reduction To High‐Concentration Pure Formic Acid Solutions In An All‐Solid‐State Reactor,” Nature Communications 11 (2020): 3633, 10.1038/s41467-020-17403-1. PMC7371694 32686669

[38] K. Xie , A. Ozden , R. K. Miao , Y. Li , D. Sinton , and E. H. Sargent , “Eliminating the Need For Anodic Gas Separation in CO_2_ Electroreduction Systems Via Liquid‐To‐Liquid Anodic Upgrading,” Nature Communications 13 (2022): 3070, 10.1038/s41467-022-30677-x. PMC9163163 35654799

[39] P. Zhu , Z.‐Y. Wu , A. Elgazzar , et al., “Continuous Carbon Capture In An Electrochemical Solid‐Electrolyte Reactor,” Nature 618 (2023): 959–966, 10.1038/s41586-023-06060-1. 37380692

[40] W. Li , Y. Zhai , Q. Xia , and X. Zhang , “An Emerging Solid‐State Electrolyte Reactor to Drive the Future of Electrochemical Synthesis,” Advanced Energy Materials 14 (2024): 202403841.

[41] C. Xia , Y. Xia , P. Zhu , L. Fan , and H. Wang , “Direct Electrosynthesis Of Pure Aqueous H_2_O_2_ Solutions up to 20% by Weight Using A Solid Electrolyte,” Science 366 (2019): 226–231, 10.1126/science.aay1844. 31601767

[42] Y. Wen , T. Zhang , J. Wang , et al., “Electrochemical Reactors for Continuous Decentralized H_2_O_2_ Production,” Angewandte Chemie International Edition 61 (2022): 202205972, 10.1002/ange.202205972. 35698896

[43] T. Chen , Z. Zhao , S. Zhang , et al., “Recent Progress in Solid‐State Electrolyte for Electrocatalytic CO_2_ Reduction,” Advanced Energy Materials 15 (2025): 2502092, 10.1002/aenm.202502092.

[44] G. Zhang , B. Tan , D. H. Mok , et al., “Electrifying HCOOH Synthesis From CO_2_ Building Blocks Over Cu–Bi Nanorod Arrays,” Proceedings of the National Academy of Sciences 121 (2024): 2400898121, 10.1073/pnas.2400898121. PMC11260142 38980900

[45] Chemanalyst , Formic Acid Price Trend and Forecast, 2025, can be found under https://www.chemanalyst.com/Pricing‐data/formic‐acid‐1242.

[46] S. Li , W. Liu , Y. Shi , et al., “Ligand‐Rich Oxygen Evolution Electrocatalysts Reconstructed From Metal‐Organic Frameworks For Anion‐Exchange Membrane Water Electrolysis,” Science Bulletin 70 (2025): 1976–1985, 10.1016/j.scib.2025.04.037. 40328607

[47] I. R. Speight , K. J. Ardila‐Fierro , J. G. Hernández , et al., “Ball Milling For Mechanochemical Reactions,” Nature Reviews Methods Primers 5 (2025): 29, 10.1038/s43586-025-00401-2.

[48] X. Ren , F. Liu , H. Wu , et al., “Reconstructed Bismuth Oxide through in situ Carbonation by Carbonate‐Containing Electrolyte for Highly Active Electrocatalytic CO_2_ Reduction to Formate,” Angewandte Chemie International Edition 62 (2023): 202316640. 10.1002/anie.202316640 38146810

[49] Y.‐C. Li , X.‐L. Zhang , X.‐L. Tai , et al., “Highly Tension‐Strained Copper Concentrates Diluted Cations for Selective Proton‐Exchange Membrane CO_2_ Electrolysis,” Angewandte Chemie International Edition 64 (2025): 202422054, 10.1002/anie.202422054. 39807802

[50] X. Chen , R. Lu , C. Li , et al., “Activating Inert Non‐Defect Sites in Bi Catalysts Using Tensile Strain Engineering For Highly Active CO_2_ Electroreduction,” Nature Communications 16 (2025): 1927, 10.1038/s41467-025-56975-8. PMC11850590 39994189

[51] Q. Huang , Z. Qian , N. Ye , et al., “In Situ Reconstructed Hydroxyl‐Rich Atomic‐Thin Bi_2_O_2_CO_3_ Enables Ampere‐Scale Synthesis of Formate From CO_2_ With Activated Water Dissociation,” Advanced Materials 37 (2025): 2415639, 10.1002/adma.202415639. 39711239

[52] L. Lin , X. He , X. Zhang , et al., “A Nanocomposite of Bismuth Clusters and Bi_2_O_2_CO_3_ Sheets for Highly Efficient Electrocatalytic Reduction of CO_2_ to Formate,” Angewandte Chemie International Edition 62 (2023): 202214959. 10.1002/anie.202214959 36307930

[53] S.‐F. Tang , X.‐L. Lu , C. Zhang , Z.‐W. Wei , R. Si , and T.‐B. Lu , “Decorating Graphdiyne On Ultrathin Bismuth Subcarbonate Nanosheets To Promote CO_2_ Electroreduction To Formate,” Science Bulletin 66 (2021): 1533–1541, 10.1016/j.scib.2021.03.020. 36654282

[54] H. Liu , T. Yan , S. Tan , et al., “Observation on Microenvironment Changes of Dynamic Catalysts in Acidic CO_2_ Reduction,” Journal of the American Chemical Society 146 (2024): 5333–5342, 10.1021/jacs.3c12321. 38369932

[55] C. Xia , P. Zhu , Q. Jiang , et al., “Continuous Production Of Pure Liquid Fuel Solutions Via Electrocatalytic CO_2_ Reduction Using Solid‐Electrolyte Devices,” Nature Energy 4 (2019): 776–785, 10.1038/s41560-019-0451-x.

[56] K.‐J. Jeong , C. M. Miesse , J.‐H. Choi , et al., “Fuel Crossover In Direct Formic Acid Fuel Cells,” Journal of Power Sources 168 (2007): 119–125, 10.1016/j.jpowsour.2007.02.062.

[57] W.‐Y. Yu , G. M. Mullen , D. W. Flaherty , and C. B. Mullins , “Selective Hydrogen Production From Formic Acid Decomposition on Pd–Au Bimetallic Surfaces,” Journal of the American Chemical Society 136 (2014): 11070–11078, 10.1021/ja505192v. 25019609

[58] X. Chen , J. Chen , H. Chen , et al., “Promoting Water Dissociation For Efficient Solar Driven CO_2_ Electroreduction Via Improving Hydroxyl Adsorption,” Nature Communications 14 (2023): 751, 10.1038/s41467-023-36263-z. PMC9918482 36765049

[59] H. Shen , Y. Zhao , L. Zhang , et al., “In‐Situ Constructuring of Copper‐Doped Bismuth Catalyst for Highly Efficient CO_2_ Electrolysis to Formate in Ampere‐Level,” Advanced Energy Materials 13 (2023): 2202818, 10.1002/aenm.202202818.

[60] G. Jia , Y. Wang , M. Sun , et al., “Size Effects of Highly Dispersed Bismuth Nanoparticles on Electrocatalytic Reduction of Carbon Dioxide to Formic Acid,” Journal of the American Chemical Society 145 (2023): 14133–14142, 10.1021/jacs.3c04727. 37317545 PMC10311520

