# Supplementary material for: Efficient Electrocatalytic Conversion of CO2 to Pure Formic Acid Solutions via Strain‐Engineered Bismuth Nanosheets
Source: Adv Sci (Weinh). 2026 Apr 9;13(36):e75152. doi: 10.1002/advs.75152 (PMC13317578; doi:10.1002/advs.75152)
Supplement: Supplementary file 1 — Supporting File: advs75152‐sup‐0001‐SuppMat.docx [file ADVS-13-e75152-s001.docx]

Supporting Information

**Efficient Electrocatalytic Conversion of CO_2_ to Pure Formic Acid Solutions via Strain-Engineered Bismuth Nanosheets**

Shiqi Li, Yuefeng Zhang, Panzhe Qiao, Cuo Wu, Xinya Tian, Yufeng Xu, Yun Zhang, Peng Wang, Tingting Li, Muhammad Afaq, Xinlin Luo, Tang Wang, Chunxiao Liu, Zi-You Yu, Chuan Xia^*^, Min-Rui Gao*, Zhen-Yu Wu*

S. Li, Y. Zhang, C. Wu, X. Tian, Y. Xu, Y. Zhang, P. Wang, T. Li, M. Afaq, X. Luo, T. Wang, Z-Y. Wu

Department of Chemistry

Institute of Major Scientific Facilities for New Materials Guangdong Provincial Key Laboratory of Sustainable Biomimetic Materials and Green Energy

Southern University of Science and Technology

Shenzhen 518055, China

E-mail: wuzy@sustech.edu.cn

P. Z. Qiao

Shanghai Synchrotron Radiation Facility

Shanghai Advanced Research Institute

Chinese Academy of Sciences

Shanghai 201210, China

C. Wu, Z-Y. Yu

MOE International Joint Laboratory of Materials Microstructure

Institute for New Energy Materials and Low Carbon Technologies

School of Materials Science & Engineering

Tianjin University of Technology

Tianjin 300384, China

C. Liu, C. Xia

School of Materials and Energy

University of Electronic Science and Technology of China

Chengdu, Sichuan 611731, China

E-mail: chuan.xia@uestc.edu.cn

M-R Gao

Division of Nanomaterials & Chemistry

Hefei National Research Center for Physical Sciences at the Microscale

University of Science and Technology of China

Hefei 230026, China

E-mail: mgao@ustc.edu.cn

**Methods**

**Chemicals**

Pristine bismuth trioxide (Bi_2_O_3_), ethanol (C_2_H_6_O), and isopropanol (C_3_H_8_O) were purchased from Aladdin (Shanghai) Chemistry Co., Ltd. Potassium bicarbonate (KHCO_3_) and dimethyl sulfoxide (DMSO) were purchased from Macklin (Shanghai) Biochemical Technology Co., Ltd. Deuterium oxide (D_2_O) was supplied by Energy Chemical. Sulfuric acid (H_2_SO_4_, 98.0%) was purchased from Yonghua Chemical Co., Ltd. Nafion binder (5 wt%, D520) and cation exchange membrane (N117) were purchased from Du Pont China Holding Co., Ltd. Porous solid Dowex (50WX8) resin (200-400 mesh, Sigma) was purchased from Sigma-Aldrich. The gas diffusion layer (GDL, YLS-30T) and anion-exchange membrane (Dioxide Materials, Grade 60) were purchased from Suzhou Sinero Technology Co., Ltd. The deionized water with a resistivity of 18.2 MΩ used in all experiments was prepared by an ultrapure water system.

**Preparation of TS-BiNs catalyst**

Typically, 500 mg of pristine Bi_2_O_3_ was dispersed into 10 mL of ethanol and then added to an agate milling jar (100 mL). The mixture was subjected to mechanochemical processing using a planetary ball mill (agate balls, 5 mm diameter) at 700 r min^–1^ for 10, 30, or 60 min (the optimal time was 30 min). The resultant product underwent sequential washing with deionized water (2 times) and ethanol (2 times) to remove residual impurities, followed by vacuum drying at 60 °C for 6 h to obtain BM-Bi. For subsequent treatment, 100 mg of the BM-Bi material was dispersed in 30 mL of 1 M KHCO_3_ aqueous solution under vigorous magnetic stirring (800 rpm) at ambient temperature for 10 or 30 min (the optimal time was 30 min). The as-obtained TS-BiNs material was collected via centrifugation (8000 r min^–1^, 10 min), washed three times with deionized water and ethanol alternately, and dried under vacuum at 60 °C for 6 h.

**Characterization of the catalysts**

The powder X-ray diffractometer (Rigaku SmartLab 9 KW) with a Cu Kα radiation source (λ = 1.5406 Å) was used to collect the samples’ X-ray diffraction (XRD) patterns. X-ray photoelectron spectroscopy (XPS) measurements were performed using a PHI 5000 VersaProbe III spectrometer (ULVAC-PHI, Japan) with monochromatic Al Kα radiation (hν = 1486.6 eV), and the binding energies were calibrated against the adventitious carbon C 1s peak at 284.4 eV. The Aberration-corrected high-angle annular darkfield scanning transmission electron microscope (HAADF-STEM) and high-resolution TEM (HRTEM) were acquired using a Titan Themis G2 Atomic Resolution Analytical Microscope operating at an accelerating voltage of 200 kV. The scanning electron microscopy (SEM) measurements were performed on a Regulus 8100 instrument at 15 kV. Atomic force microscopy (AFM) was performed on a Bruker Dimension ICON atomic force microscope. The N_2_ adsorption-desorption isotherm measurements were performed at 77 K using an ASAP 2460 Version 3.01 analyzer (Micromeritics, USA). The liquid products were quantified using ^1^H and ^13^C nuclear magnetic resonance (NMR) spectroscopy, obtained on a Bruker AVANCE NEO 400 MHz spectrometer. The X-ray absorption near-edge structure (XANES) and extended X-ray absorption fine structure (EXAFS) spectra were conducted on the BL14W1 beamline of the Shanghai Synchrotron Radiation Facility (SSRF) and analyzed with IFEFFIT Athena and Artemis software.

**GPA strain mapping**

Strain mapping of various catalysts was carried out using the open-source Strain++ program by J.J.P. Peters, based on the geometric phase analysis (GPA) algorithm from Martin J. Hÿtch’s paper^1^. GPA measures and maps strain fields in an AC-TEM image by comparing the phase of crystalline facets in the image with that of perfect facets defined by a g-vector. Specifically, the AC-TEM image undergoes a Fourier transform, and two non-collinear strong reflection spots are selected. After the inverse Fourier transform of these spots, the 2D displacement field is deduced from the phase difference between the resulting complex images and the perfect lattice. The strain field is then derived from the displacement field’s derivatives. The strain range was set from –5% to 5%.

**Strain calculation**

According to XRD and HADDF-STEM analyses, the main exposed crystal face for TS-BiNs catalyst after CO_2_RR is Bi (012). The d-spacing of Bi (012) can be obtained by measuring the lattice highlighted in AC-TEM images. Since the d-spacing of standard Bi (012) facets is 3.28 Å, the strain values of TS-BiNs catalysts after CO_2_RR can thus be calculated by the following formula (note that positive values represent the tensile strain):

$$\begin{aligned} Strain=\frac{d_{Bi(012)}-3.28}{3.28}\times100\%\#\left( 1 \right) \end{aligned}$$

**Electrode preparation and electrochemical CO_2_ reduction tests**

40 mg of catalyst powder was dispersed in 2 mL of isopropanol-water mixed solution (2:1 volume ratio). Then, 160 μL Nafion binder was added to the above mixture and sonicated for 2 hours to form a homogeneous ink. Then, the ink was spray-coated onto a 5*5 cm^2^ YLS-30T GDL to obtain the working electrode (around 1 mg cm^–2^). Electrochemical tests were performed with a CHI-1140D electrochemistry workstation (Shanghai ChenHua Instrument Co. Ltd., China). Electrochemical impedance spectra (EIS) were recorded with a frequency range from 10^5^ to 1 Hz at a direct current potential of –1.0 V versus RHE (unless otherwise stated, all potentials reported are versus RHE). In this study, 1.0 M KHCO_3_ aqueous solution (pH = 8.4) was used as the electrolyte. All the potentials were converted to a RHE according to the following equation:

$$\begin{aligned} E \left( V \mathrm{versus}\mathrm{RHE} \right)=E \left( V \mathrm{versus} Ag/AgCl \right)+0.197 V+0.0591\times pH\#\left( 2 \right) \end{aligned}$$

The electrochemical double layer capacitance (C_dl_) curves of catalysts were measured by cyclic voltammetry in a non-Faradic region (0.276–0.306 V) at varied scan rates of 10, 20, 30, 40, 50, 60, 70, 80, 90, and 100 mV s^−1^. The slope of the fitting line was equal to twice the capacitance of the geometric double layer. The ECSA of the catalyst was estimated according to the equation:

$$\begin{aligned} ECSA=\frac{C_{\mathrm{dl}}\times A_{\mathrm{geo}}}{C_{s}^{*}}\#\left( 3 \right) \end{aligned}$$

where C_s_^*^ represent the specific capacitance of the catalyst (0.02 mF cm^–2^), A_geo_ is the geometric surface area of the electrode (in this case, 1 cm^2^). To calculate j_ECSA_ of catalysts, we normalized the measured current by the ECSA from the equation:

$$\begin{aligned} j_{\mathrm{ECSA}}=\frac{i}{\mathrm{ECSA}}\#\left( 4 \right) \end{aligned}$$

where i is the measured current, and ECSA is the ECSA value.

For the flow cell tests, catalyst-loaded YLS-30T GDL electrodes were used as the CO_2_RR working electrode. A nickel foam electrode (1 cm^2^) and Ag/AgCl (saturated KCl) were used as the counter electrode for water oxidation and the reference electrode, respectively. The working and counter electrodes were placed on opposite sides of two 0.5 cm-thick polytetrafluoroethylene sheets, each containing a 0.5 cm-wide by 2 cm-long channel so that the catalyst layer interfaced with the flowing liquid electrolyte. The two polytetrafluoroethylene sheets sandwiched Nafion 117 to separate the chambers. The geometric surface area of the catalyst is 1 cm^2^. CO_2_ was purged to the working electrode with a flow rate of 20 sccm using a mass flow controller (Alicat). The flow rate of 1.0 M KHCO_3_ electrolyte was 5 mL min^−1^ in both chambers controlled by a peristaltic pump. During long-term stability tests, potassium formate salt accumulation occasionally blocked the cathode GDL. When observed, tests were paused to disassemble the cell, rinse the GDL with water to remove salts, dry it under an infrared lamp, and reassemble for testing.

For the two-electrode SSE reactor tests, an anion-exchange membrane (Sustainion X37-50, Grade 60) and a cation-exchange membrane (Nafion 117) were used for HCOO^−^ and H^+^ migration, respectively. Catalyst-loaded YLS-30T GDL electrode (4 cm^2^ electrode area) and IrO_2_ electrode (Fuel Cell Store) were used as a cathode and an anode, respectively. The cathode side was supplied with 20 sccm of humidified CO_2_ gas. The anode side was provided with 0.5 M H_2_SO_4_ aqueous solution at 2.5 mL min^−1^. The porous styrene-divinylbenzene sulfonated copolymer (200-400 mesh) was used as a solid ion conductor. Deionized water with a 1-150 mL h^−1^ flow rate was used to release the HCOOH produced within the solid electrolyte layer. All the measured potentials using a two-electrode setup were not iR-corrected.

**CO_2_ reduction product analysis**

The gaseous products obtained in the CO_2_RR were analyzed by an online gas chromatograph (Shimadzu GC-2014) equipped with packed columns as well as a thermal conductivity detector (TCD) and a flame ionization detector (FID). The Faradaic efficiency of a gaseous product (FE gas) was calculated using the following equation:

$$\begin{aligned} \mathrm{FE}_{\mathrm{gas}} \left( \% \right)=\frac{J_{\mathrm{gas}}}{J_{\mathrm{total}}}\times100\%=\frac{Z\times F\times P\times V_{\mathrm{gas}}\times\varphi_{\mathrm{gas}}}{R\times T\times J_{\mathrm{total}}}\times100\%\#\left( 5 \right) \end{aligned}$$

J_gas_ and J_total_ are the partial current density for the product and the total current density, respectively. Z, F, P, V_gas_, $\varphi$_gas_, R, and T represent the number of electrons transferred, the Faraday constant (96485 C mol^−1^), the atmospheric pressure (101325 Pa), the gas flow rate at the cathode side outlet of the flow cell, the volume fraction of the gaseous product, the molar gas constant (8.314 J mol^−1^ K^−1^), and the temperature (298.15 K), respectively.

The ^1^H NMR spectra were recorded on a Bruker 400 MHz NMR spectrometer to quantify the liquid products. Typically, 500 μL of the electrolyte after electrolysis was mixed with 100 μL D_2_O and 100 μL DMSO as the internal standard for measurement. ^13^C NMR spectra were acquired using a Bruker 400 MHz NMR spectrometer to check the purity of the as-prepared HCOOH solution. Typically, 500 µL of electrolyte after electrolysis was mixed with 100 µL of Dimethyl sulfoxide-d6. The calculations of the products’ Faradaic efficiency are as follows,

$$\begin{aligned} FE=\frac{n\times c\times F\times V_{\mathrm{electrolyte}}}{Q}\times100\%\#\left( 6 \right) \end{aligned}$$

where n is the number of transferred electrons, F is the Faraday constant (96485 C mol^−1^), V_electrolyte_ is the volume of electrolyte, c is the molar concentration, and Q is the total charges consumed during CO_2_RR.

The energy efficiency (EE) for CO_2_RR to HCOOH was calculated as follows:

$$\begin{aligned} \mathrm{EE}\left( \% \right)=\frac{(1.23-E_{\mathrm{HCOOH}})\times\mathrm{FE}_{\mathrm{HCOOH}}}{E}\times100\%\#\left( 7 \right) \end{aligned}$$

The kinetic potential E_HCOOH_ for the CO_2_RR to HCOOOH is −0.2 V, and E represents the applied voltage in the SSE reactor.

**In situ Raman measurements**

In situ Raman measurements were conducted on a Labram HR Evolution confocal Raman microscope with a 532 nm excitation laser. The cell (Linglu Instrument) was used to collect in situ Raman data under the electrochemical CO_2_ reduction process. During the tests, the glassy carbon electrode coated with catalysts, the Ag/AgCl (saturated KCl) electrode, and the Pt wire were used as the work electrode, reference electrode, and counter electrode, respectively. 1.0 M KHCO_3_ was used as the electrolyte and added to the cell before the test, and pure CO_2_ was continuously injected during the test. The potential-dependent Raman spectra were collected at various potentials ranging from OCP to −0.9 V versus RHE in 1.0 M KHCO_3_ with CO_2_.

**In situ DEMS measurements**

The product generation of HCOOH and the possible intermediate product experiments were monitored by an online electrochemical mass spectrometer (Linglu Instrument). An Au film was used as the working electrode, while a platinum wire and a KCl-saturated Ag/AgCl electrode served as the counter and reference electrodes, respectively. First, TS-BiNs were loaded on the Au film and pre-reduced in the CO_2_-saturated 1 M KHCO_3_ electrolyte with three cycles (0.2 to –1.0 V vs RHE). Then, the chronoamperometry method characterized the catalysts in CO_2_-saturated 1.0 M KHCO_3_ solution. The product signals with m/z = 2, 28, 44, 45, and 46 were probed by online mass spectroscopy at different potentials.

**Density function theory calculation**

Spin-polarized density functional theory (DFT) calculations were carried out using the Vienna ab initio simulation package (VASP)^2^. Projected-augmented wave (PAW) pseudopotentials^3^ were employed to describe the ion-electron interactions. The exchange-correlation energy was treated using the Perdew−Burke−Ernzerhof (PBE) functional within the generalized gradient approximation method^4^. A kinetic energy cutoff of 450 eV was employed for the facet-wave basis set. Van der Waals corrections were included using the zero-damping D3 dispersion model developed by Grimme et al^5^. Based on the HAADF-STEM and XRD images (**Figure S33-S36**), a Bi (012) slab model consisting of four layers was constructed. The bottom two layers were fixed at the bulk position, while the other layers were fully relaxed until reaching the convergence criteria. The Brillouin zone was sampled using the Gamma-centered scheme, with a 3 × 2 × 1 k-point grid for all models. Convergence criteria for the total energy and atomic force were set at 10−5 eV and 0.05 eV/Å, respectively. A vacuum layer of 16 Å thickness was set to eliminate artificial interactions between periodic images along the z-direction. The charge transfer was analyzed using the Bader charge method^6^. The projected crystal orbital Hamilton population (pCOHP)^7^ was employed to analyze the bonding/anti-bonding population between the catalysts and OCHO intermediates. VASPKIT was used to post-process the electronic and thermodynamic properties of structures^8^.

The reaction Gibbs free energies of each elementary step were calculated based on the computational hydrogen electrode (CHE) model proposed by Nørskov et al.^9^:

$$\Delta G=\Delta E+\Delta E_{ZPE}-T\Delta S+\int C_{p}dT+\Delta G_{solvation} (8)$$

where $\Delta E$, $\Delta E_{ZPE}$, $\Delta S$, and $C_{p}$ were the changes in the electronic total energy, zero-point energy, entropy, and heat capacity, respectively. T was the temperature of 298.15 K. $\Delta G_{solvation}$ is the solvation corrections due to the strong hydrogen bonding effects with the values referred from Ref.[^10^]. The entropies and vibrational frequencies of molecules in the gas phase were taken from the NIST database (Computational Chemistry Comparison and Benchmark Database.).


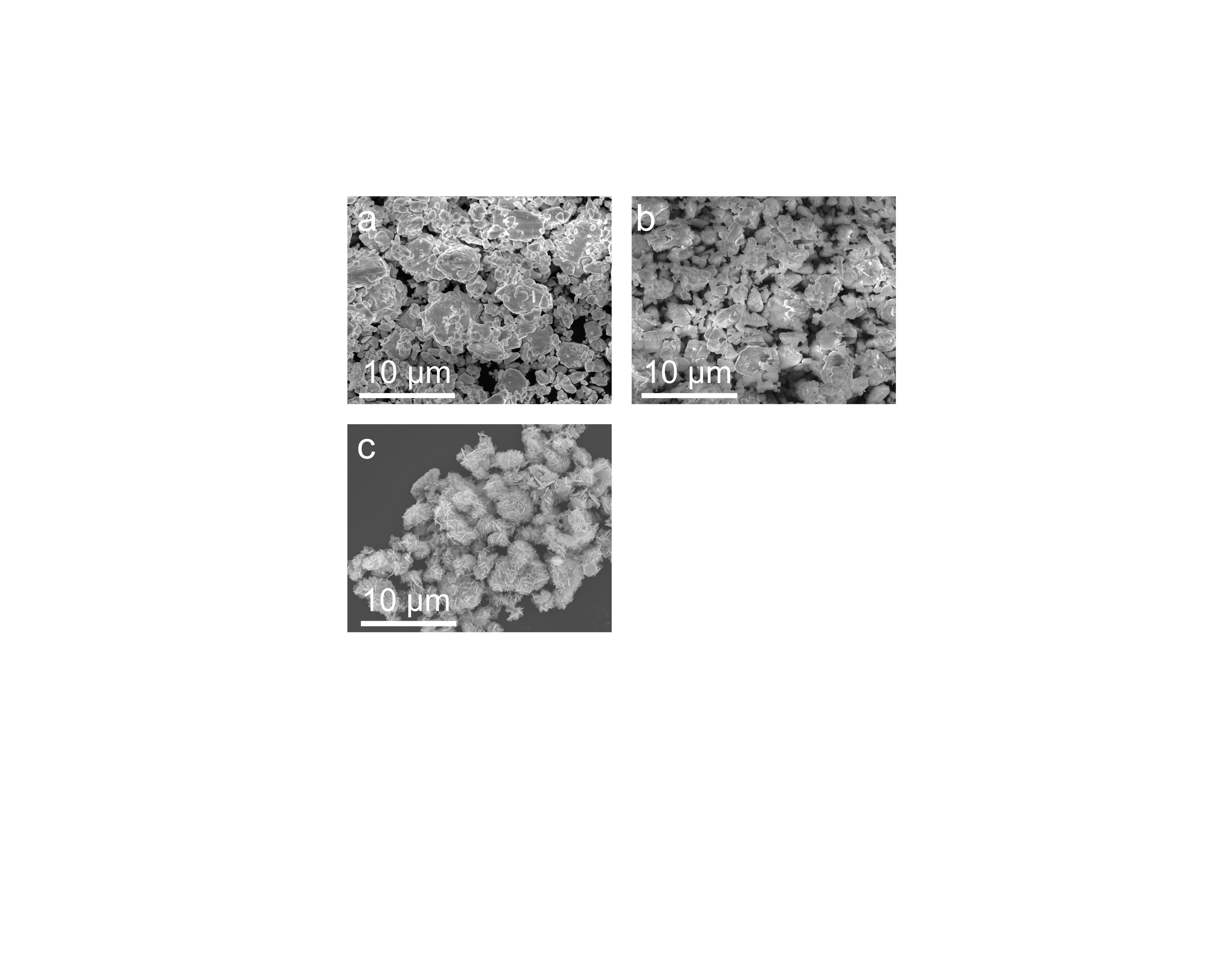


**Figure S1**. SEM images of (a) Com-Bi, (b) BM-Bi, and (c) TS-BiNs.

Com-Bi exhibits irregular aggregates with broadly distributed particle sizes ranging from ~500 nm to over 10 μm (**Figure S1a**). It is found that Com-Bi underwent a profound structural change induced by mechanochemical ball milling, resulting in a reduction in the particle size to *ca.* 2 μm and a more uniform distribution (**Figure S1b**).


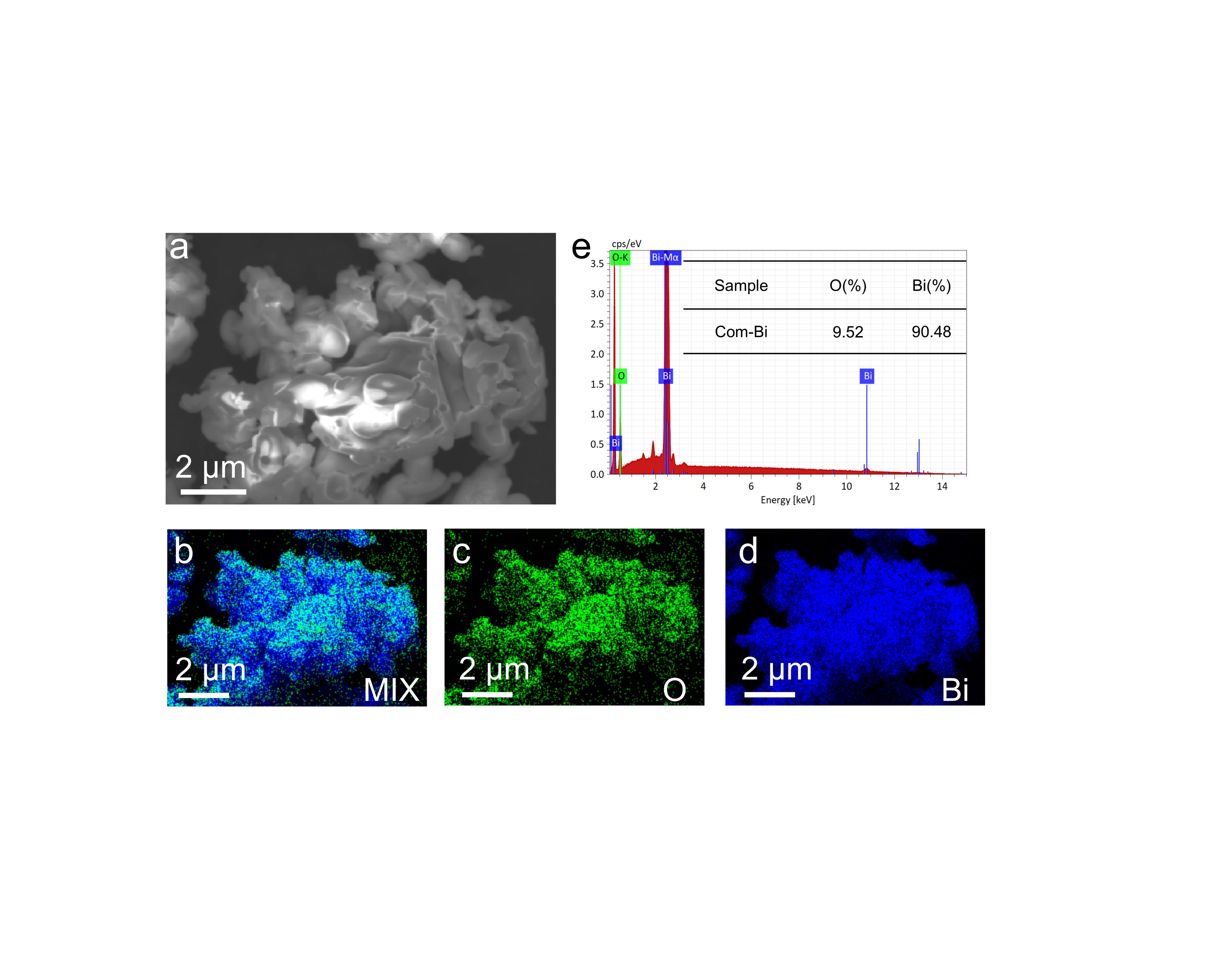


**Figure S2**. (a-e) EDS elemental mapping analysis of Com-Bi.


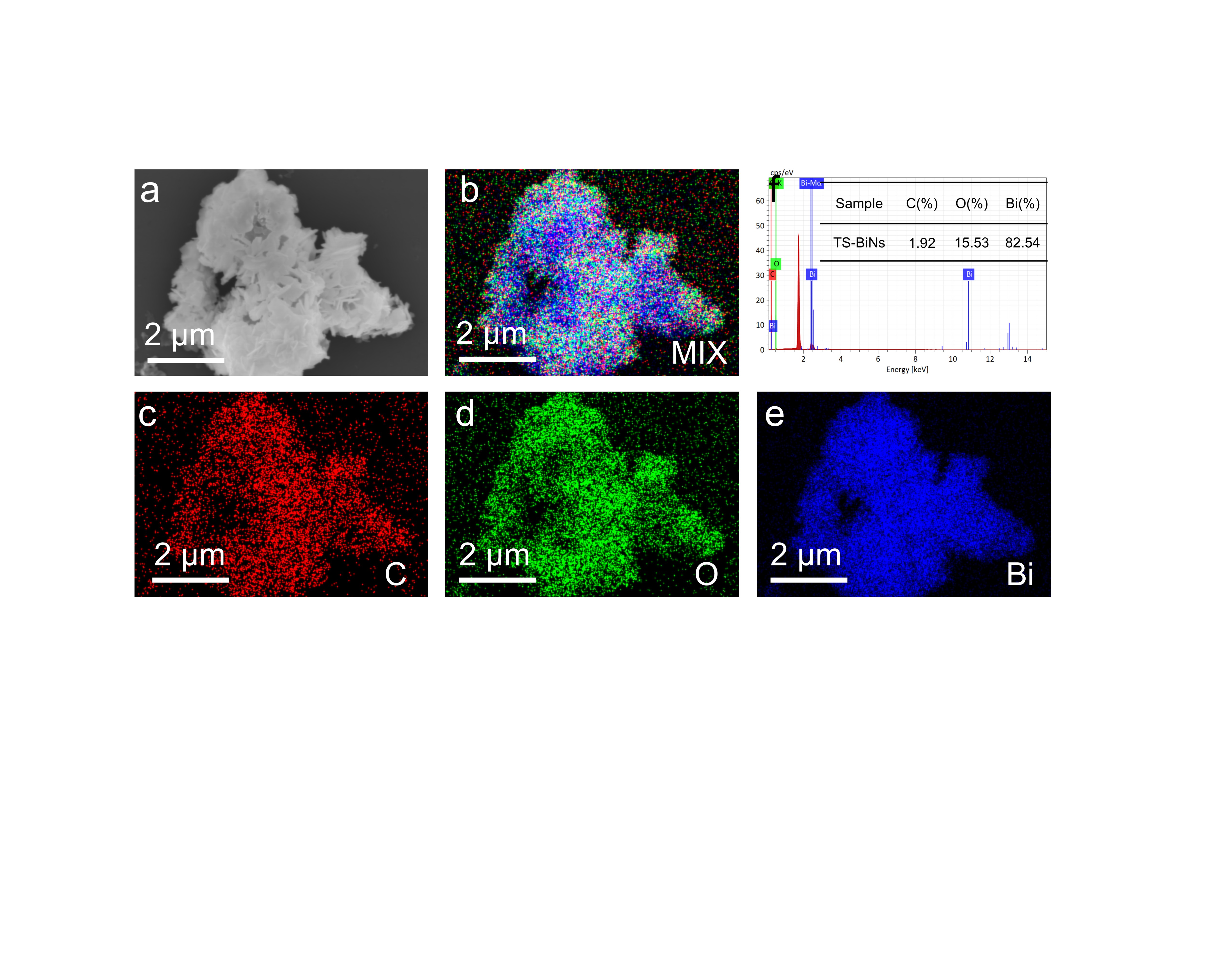


**Figure S3**. (a-f) EDS elemental mapping analysis of TS-BiNs.


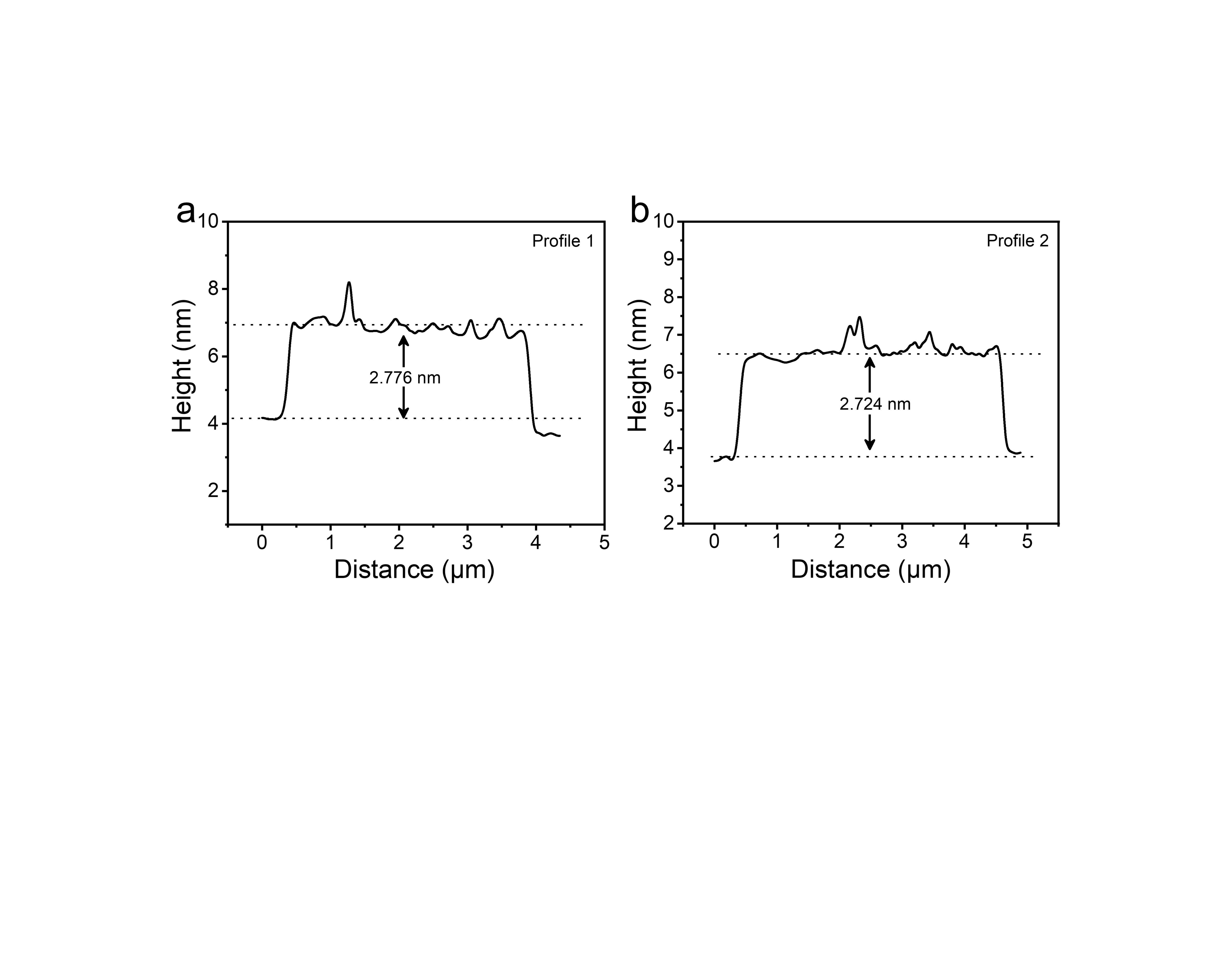


**Figure S4**. (a, b) The thickness profiles of TS-BiNs, corresponding to Figure S 1d.


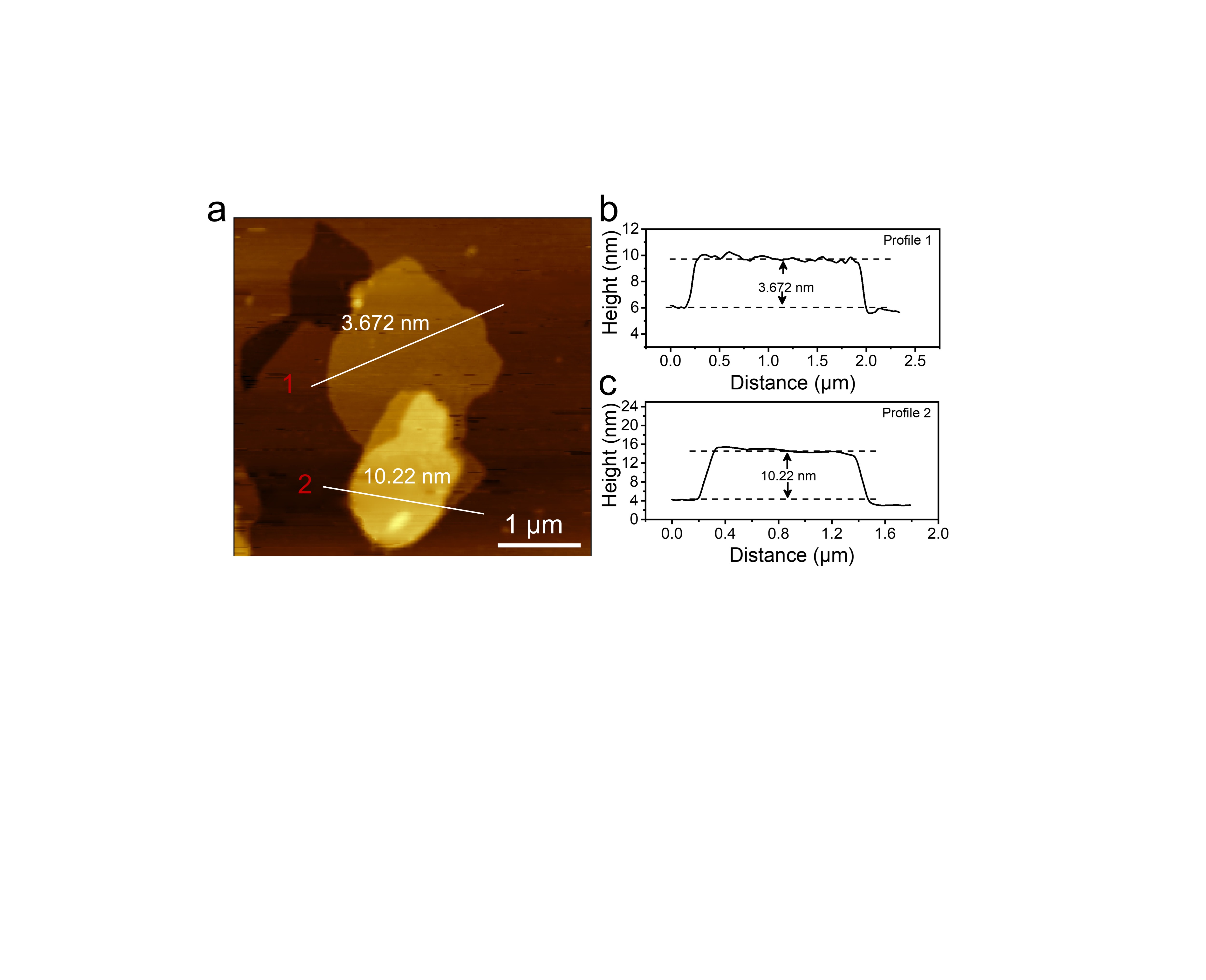


**Figure S5**. (a) Representative AFM image of Com-Bi without ball-milling after carbonation, and (b, c) the corresponding thickness profiles.


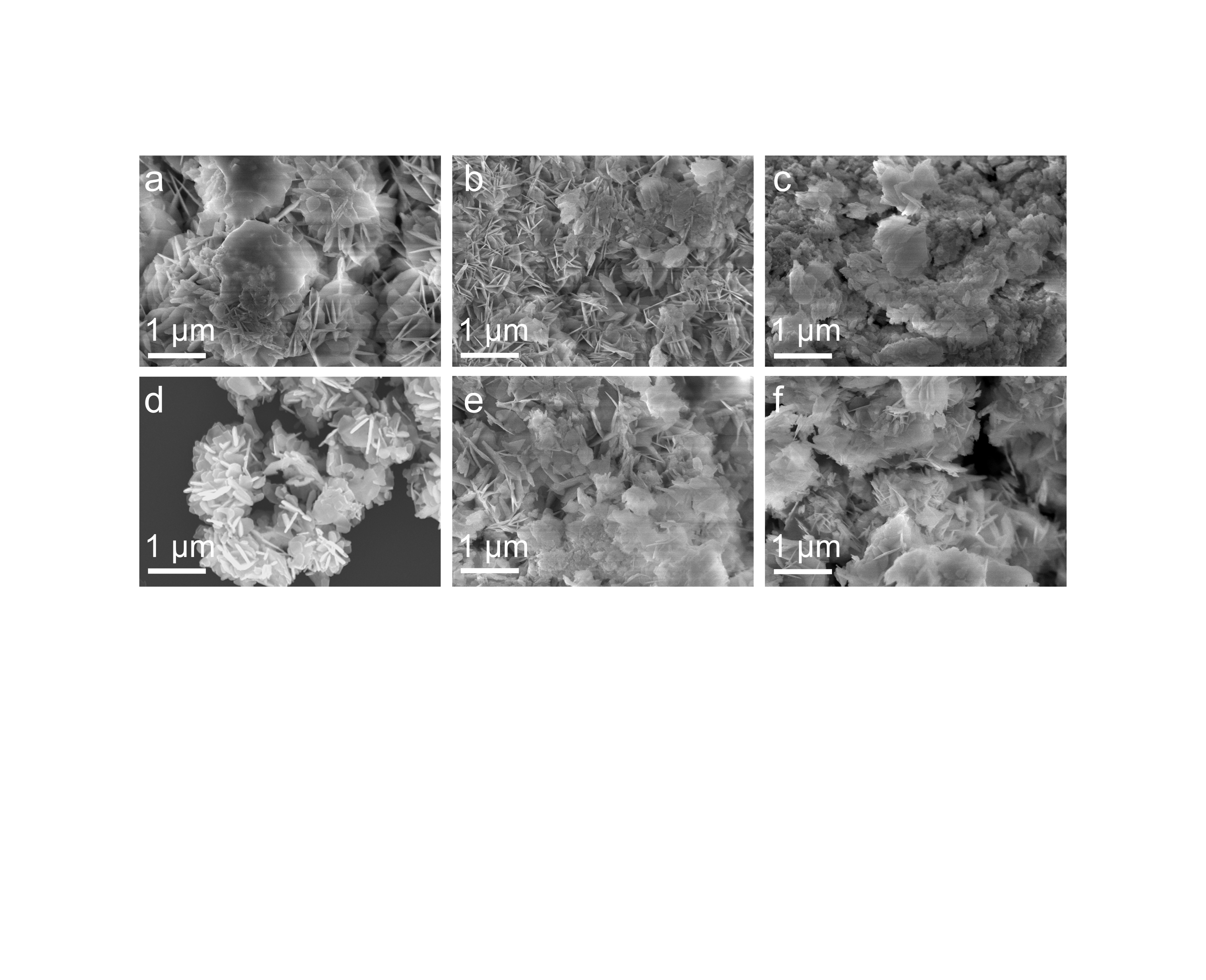


**Figure S6**. SEM images of (a) BM-10 min-C-10 min, (b) BM-10 min-C-30 min, (c) BM-30 min-C-10 min, (d) BM-30 min-C-30 min (TS-BiNs), (e) BM-60 min-C-10 min, and (f) BM-60 min-C-30 min.


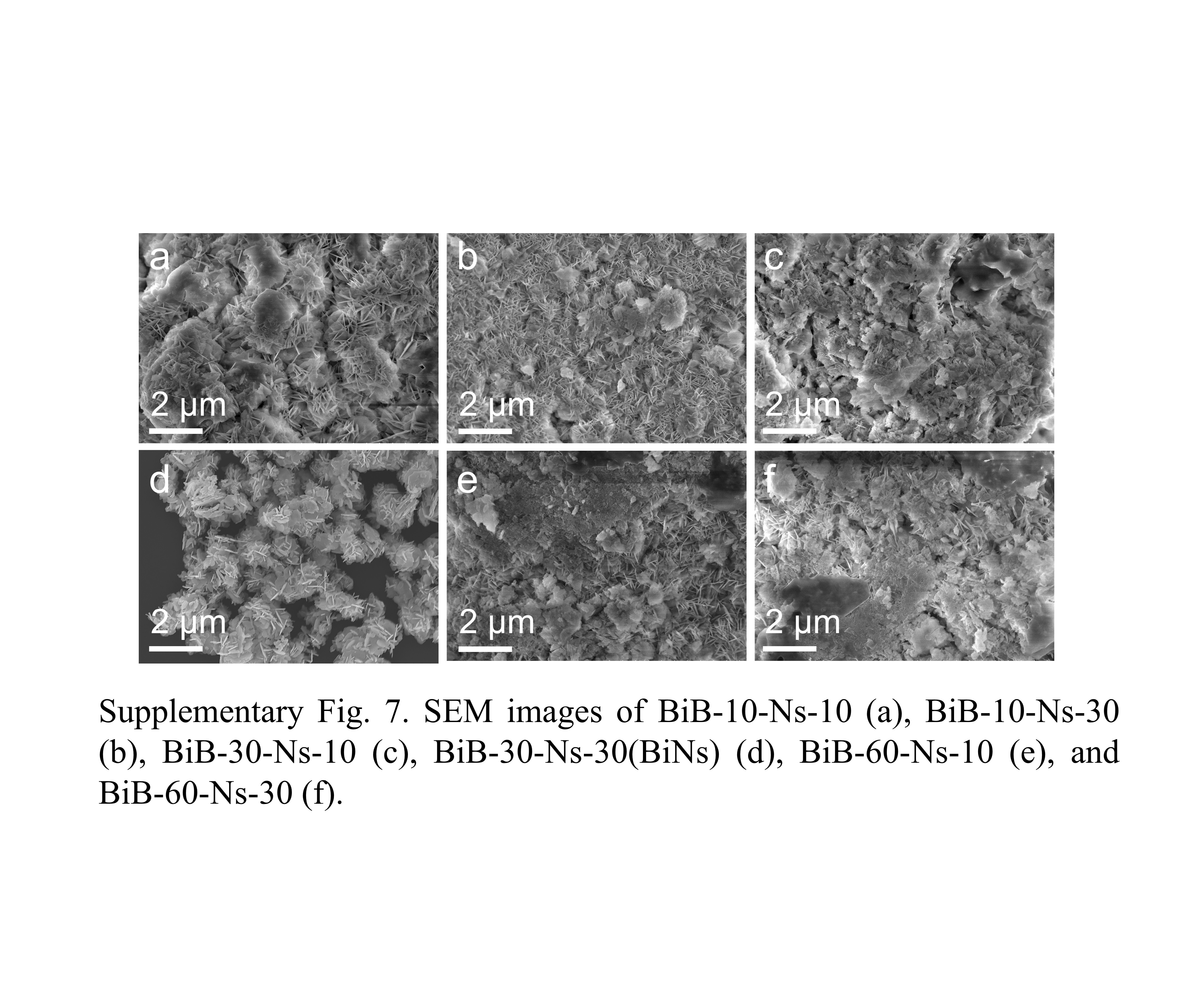


**Figure S7**. SEM images of (a) BM-10 min-C-10 min, (b) BM-10 min-C-30 min, (c) BM-30 min-C-10 min, (d) BM-30 min-C-30 min (TS-BiNs), (e) BM-60 min-C-10 min, and (f) BM-60 min-C-30 min.


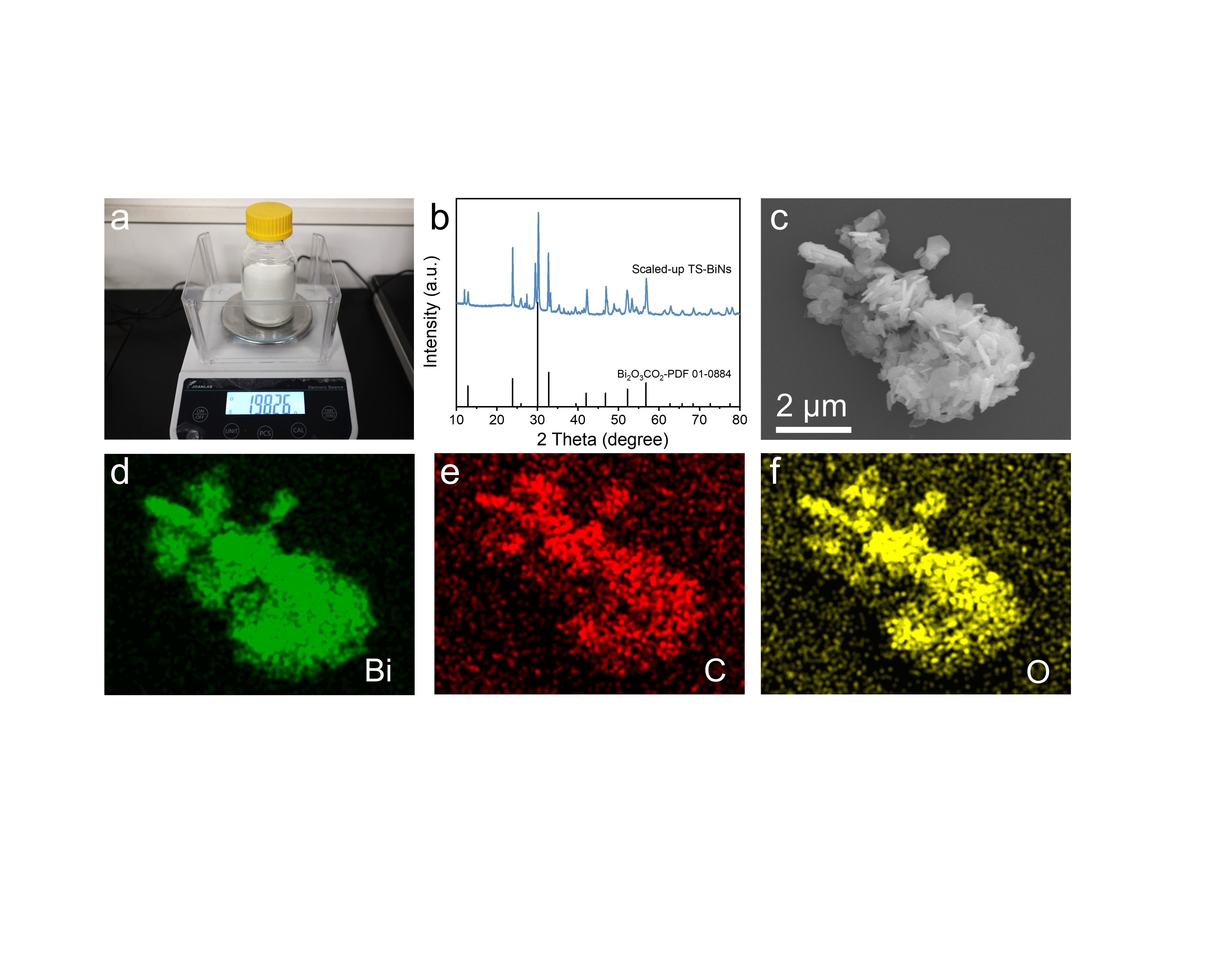


**Figure S8**. Characterizations of the scale-up preparation of TS-BiNs. (a) Digital image of the synthesized ~200 g TS-BiNs catalyst, demonstrating that our synthesis strategy can be easily scaled up. (b) PXRD pattern, (c) SEM image, and (d-f) EDS mapping images of TS-BiNs.

The scaled-up catalyst, characterized by SEM, EDS, and PXRD, exhibits the same morphology and composition as the small-scale sample, demonstrating that the ball milling-carbonation process has substantial potential for translation from laboratory-scale preparation to industrial production.


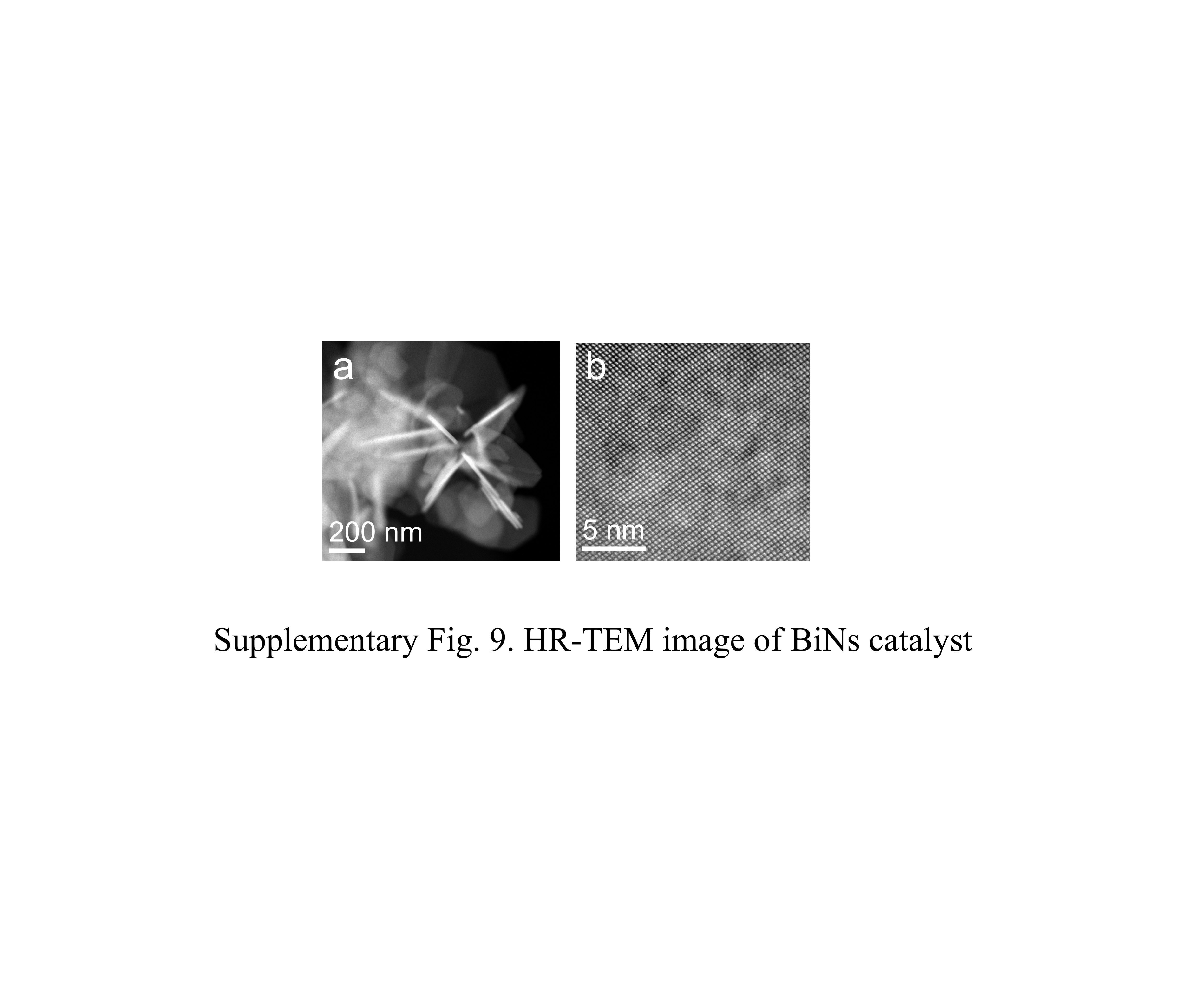


**Figure S9**. (a, b) HAADF-STEM image of TS-BiNs catalyst.


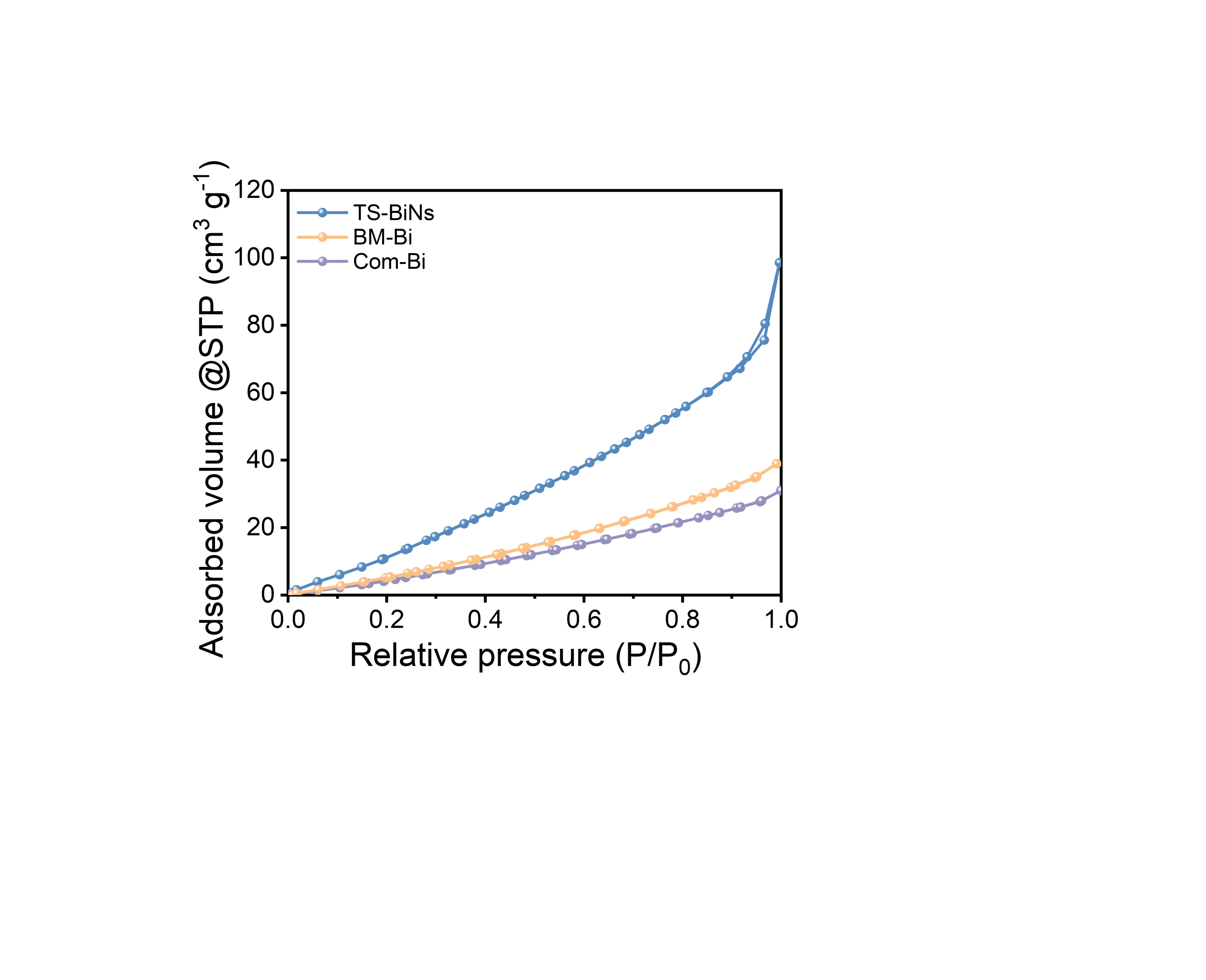


**Figure S10**. N_2_ adsorption/desorption isotherms of TS-BiNs, BM-Bi, and Com-Bi.


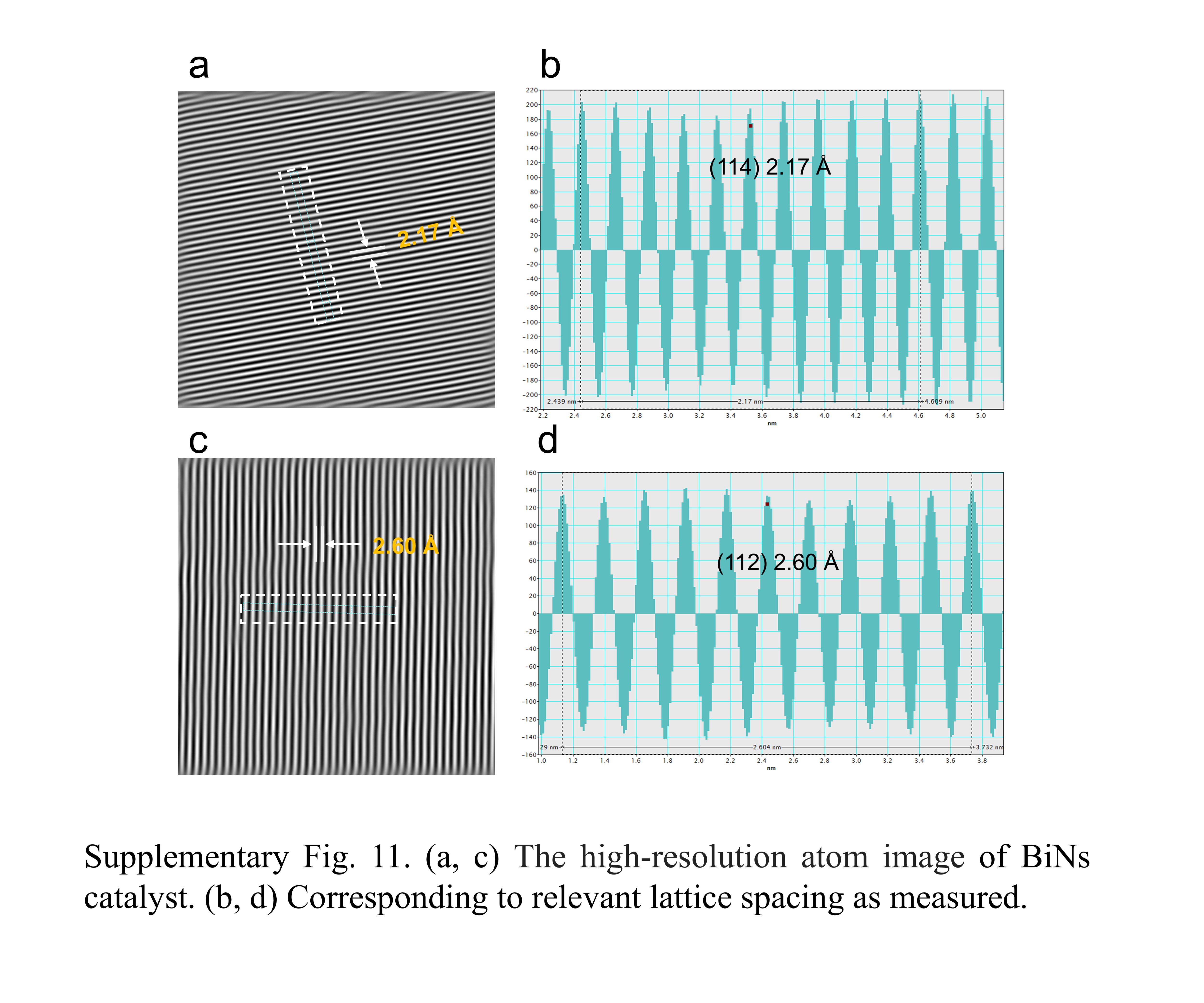


**Figure S11**. (a, c) The high-resolution atom images of TS-BiNs catalyst. (b, d) Corresponding lattice spacings measured experimentally.


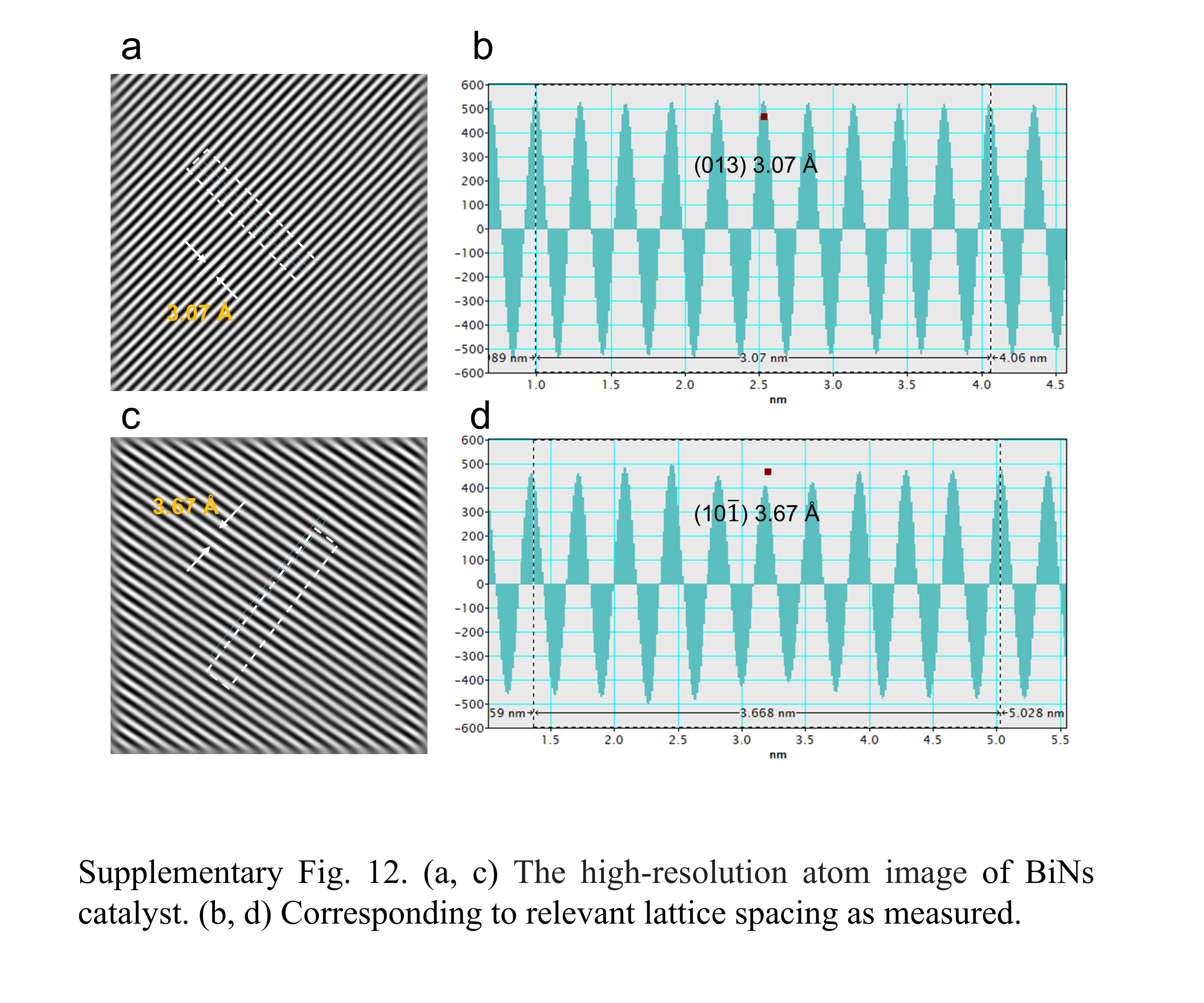


**Figure S12**. (a, c) The high-resolution atom image of TS-BiNs catalyst. (b, d) Corresponding lattice spacings measured experimentally.


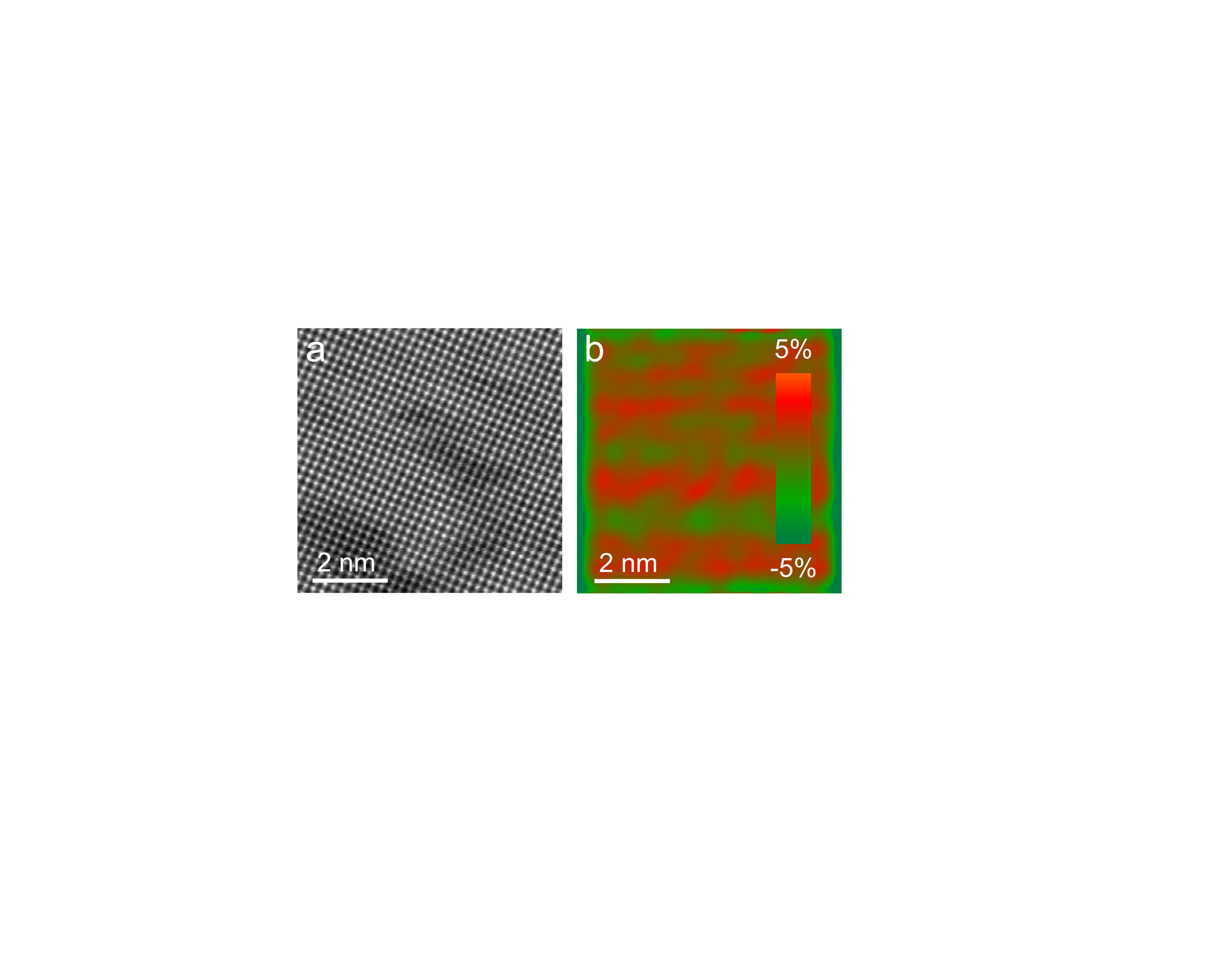


**Figure S13**. (a) HAADF-STEM image of TS-BiNs. (b) The corresponding HAADF-STEM-GPA strain analysis-ε_yy_.


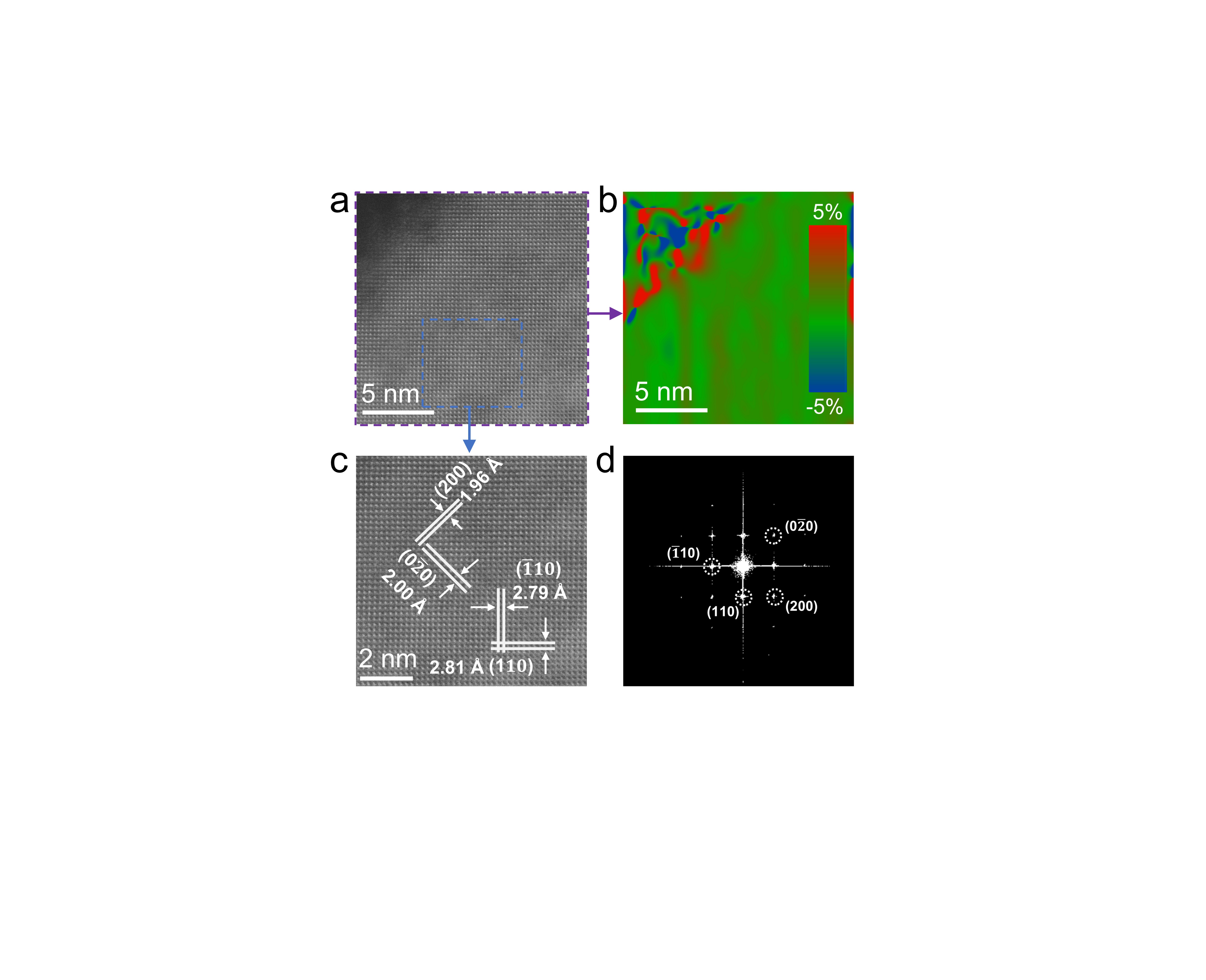


**Figure S14**. (a) HAADF-STEM image of Com-Bi after carbonation. (b) HAADF-STEM-GPA strain analysis-ε_yy_. (c) The high-resolution atom image (enlarged view of the blue box in (a). (d) The corresponding Fast Fourier Transform (FFT) pattern for the carbonated Com-Bi sample without ball milling.


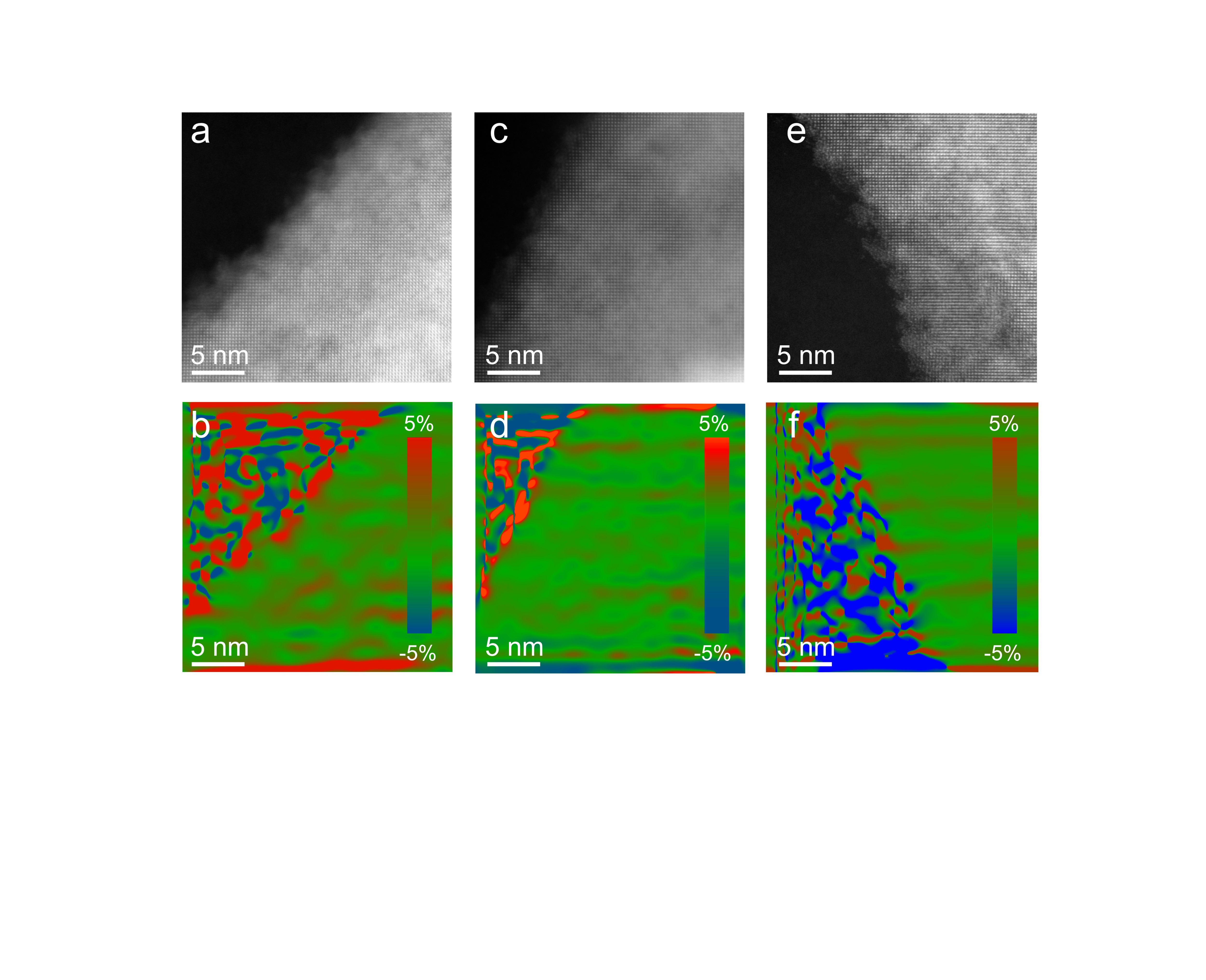


**Figure S15**. (a, c, and e) HAADF-STEM images of Com-Bi without ball milling after carbonation. (b, d, and f) The corresponding HAADF-STEM-GPA strain analysis-ε_yy_.


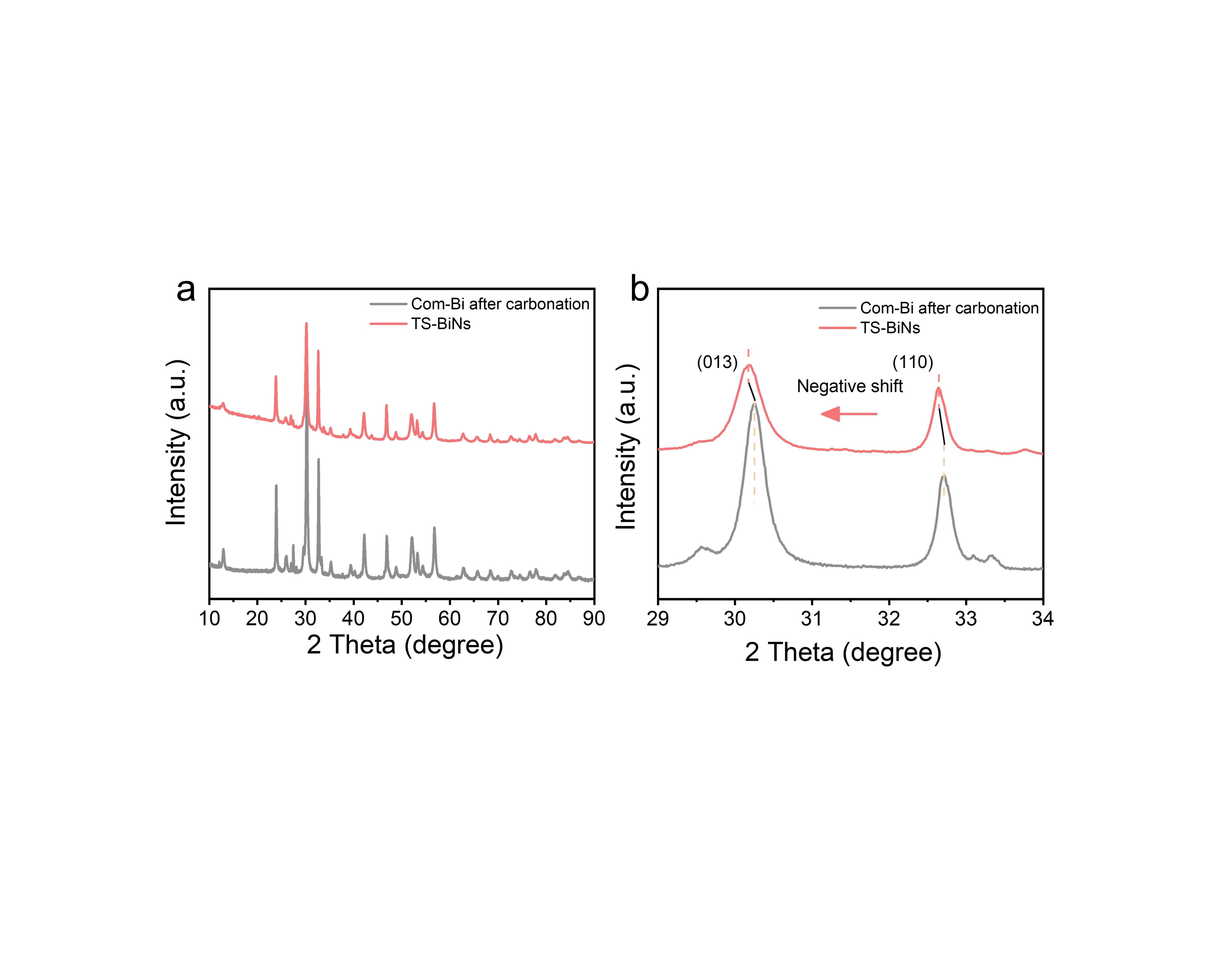


**Figure S16**. (a, b) XRD spectra of pristine Com-Bi after carbonation and TS-BiNs.


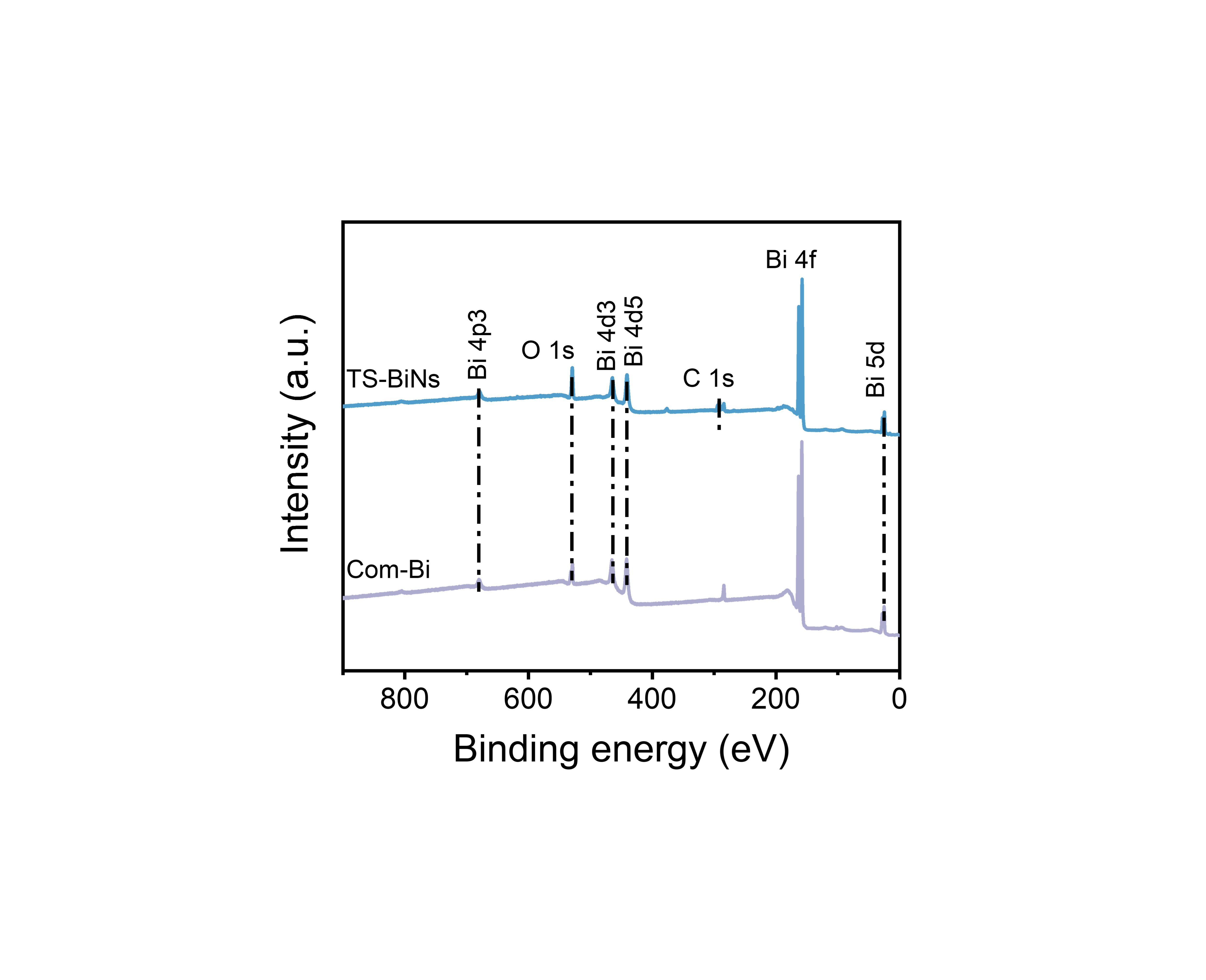


**Figure S17**. XPS survey spectra for pristine TS-BiNs and Com-Bi. Of note, the C 1s peak observed in Com-Bi is attributed to carbon contamination from the surrounding environment.


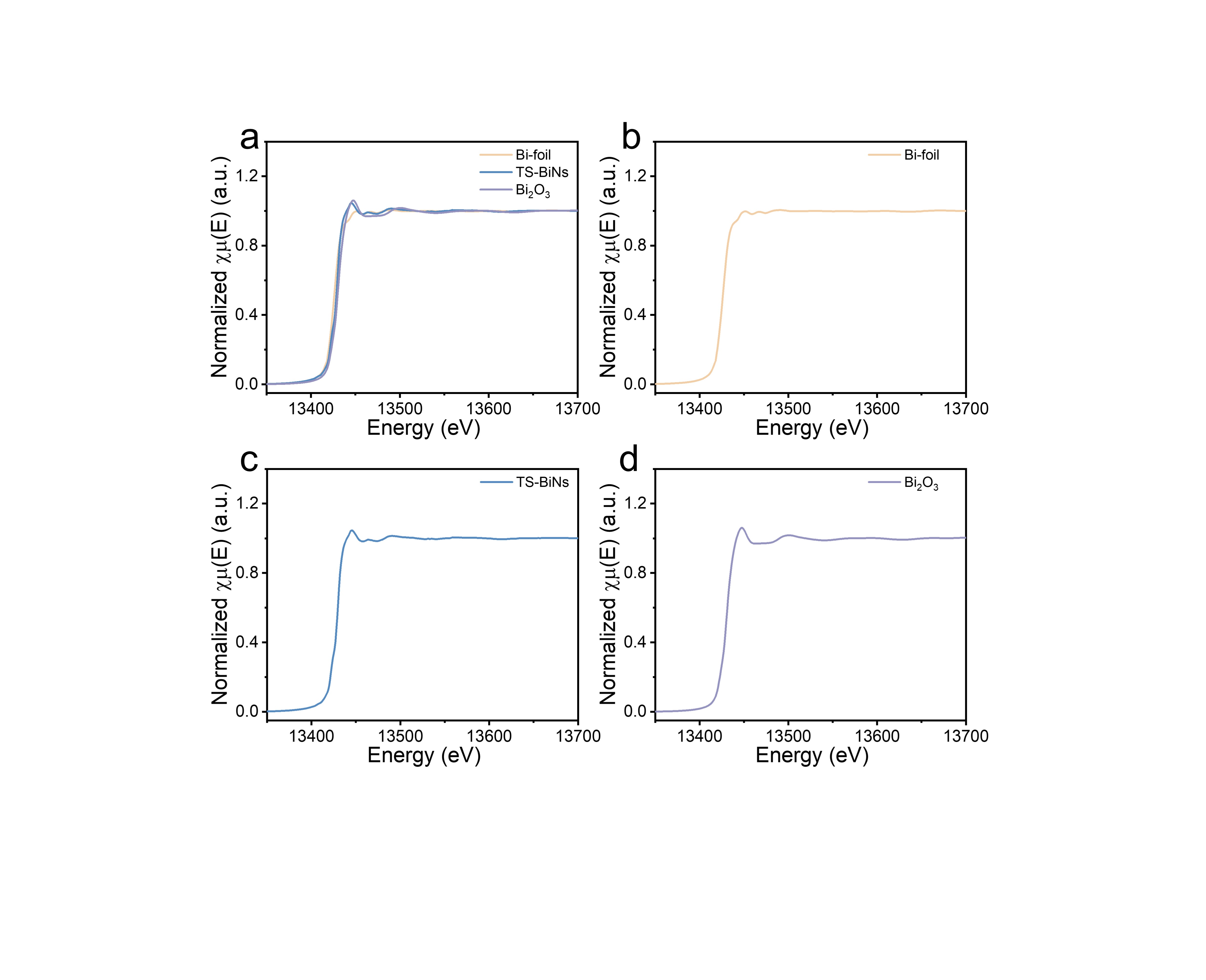


**Figure S18**. (a) Normalized Bi L_3_-edge XAS spectra of all samples, (b) Bi-foil, (c) TS-BiNs, and (d) Bi_2_O_3_.


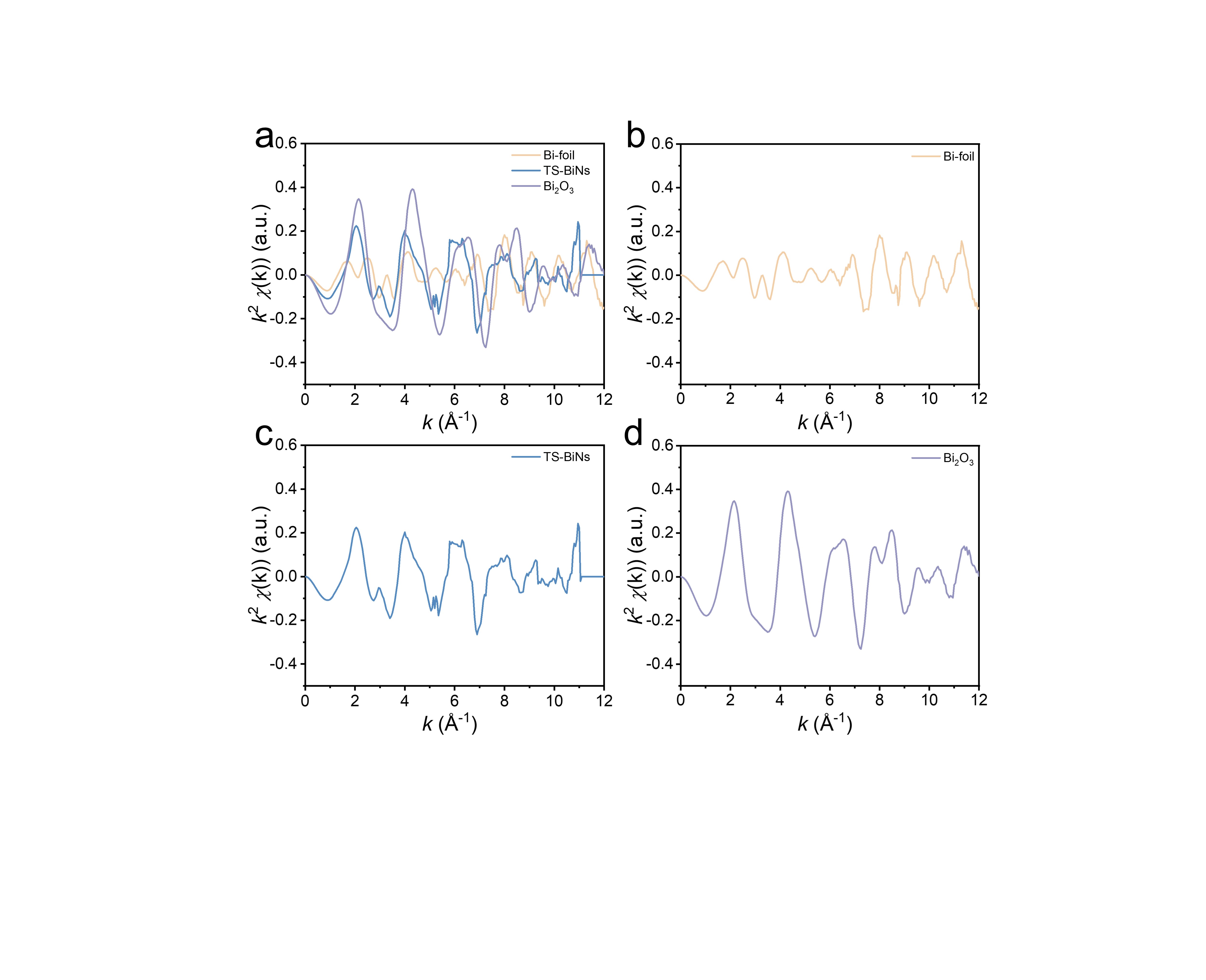


**Figure S19**. (a) Bi L_3_-edge EXAFS of oscillation curves of all samples, (b) Bi-foil, (c) TS-BiNs, and (d) Bi_2_O_3_.


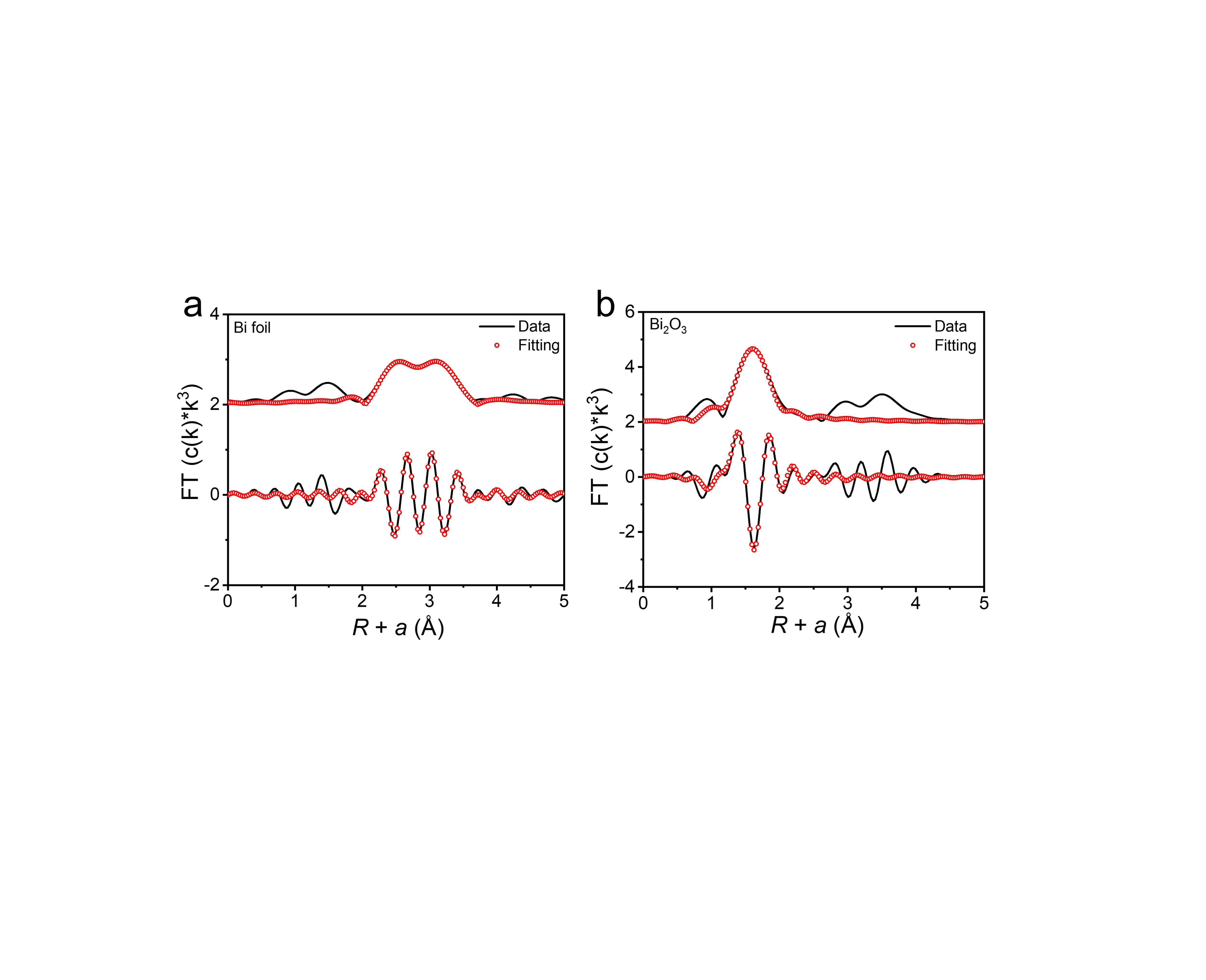


**Figure S20**. Bi L_3_-edge EXAFS (black lines) and fitting curves (red points) for Bi foil (a) and Bi_2_O_3_ (b) in R-space, including FT magnitude (up) and imaginary component (down). The data are k^3^-weighted without phase correction.


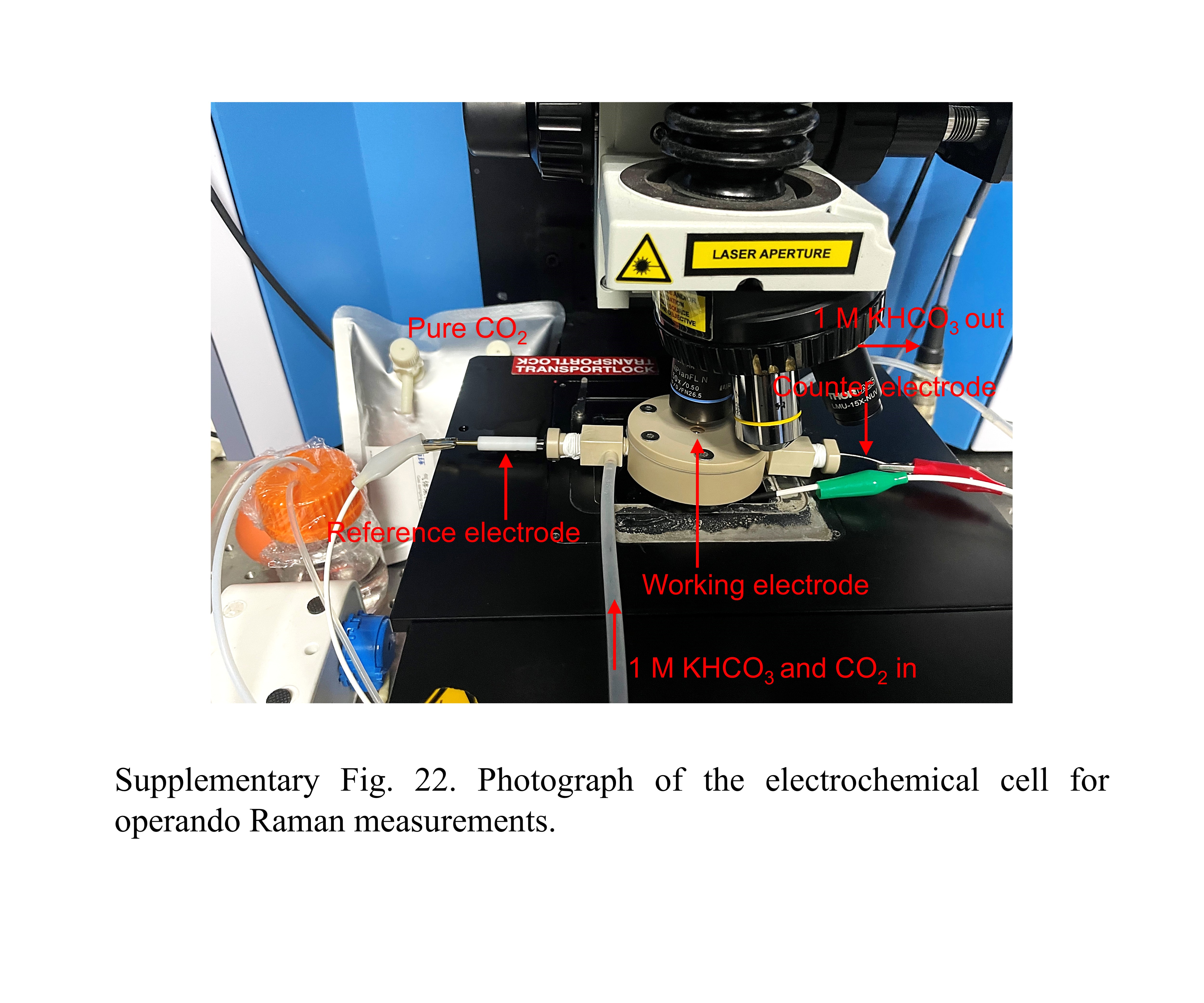


**Figure S21**. Photograph of the electrochemical cell for in situ Raman measurements (Pure CO_2_ was continuously injected during the test).


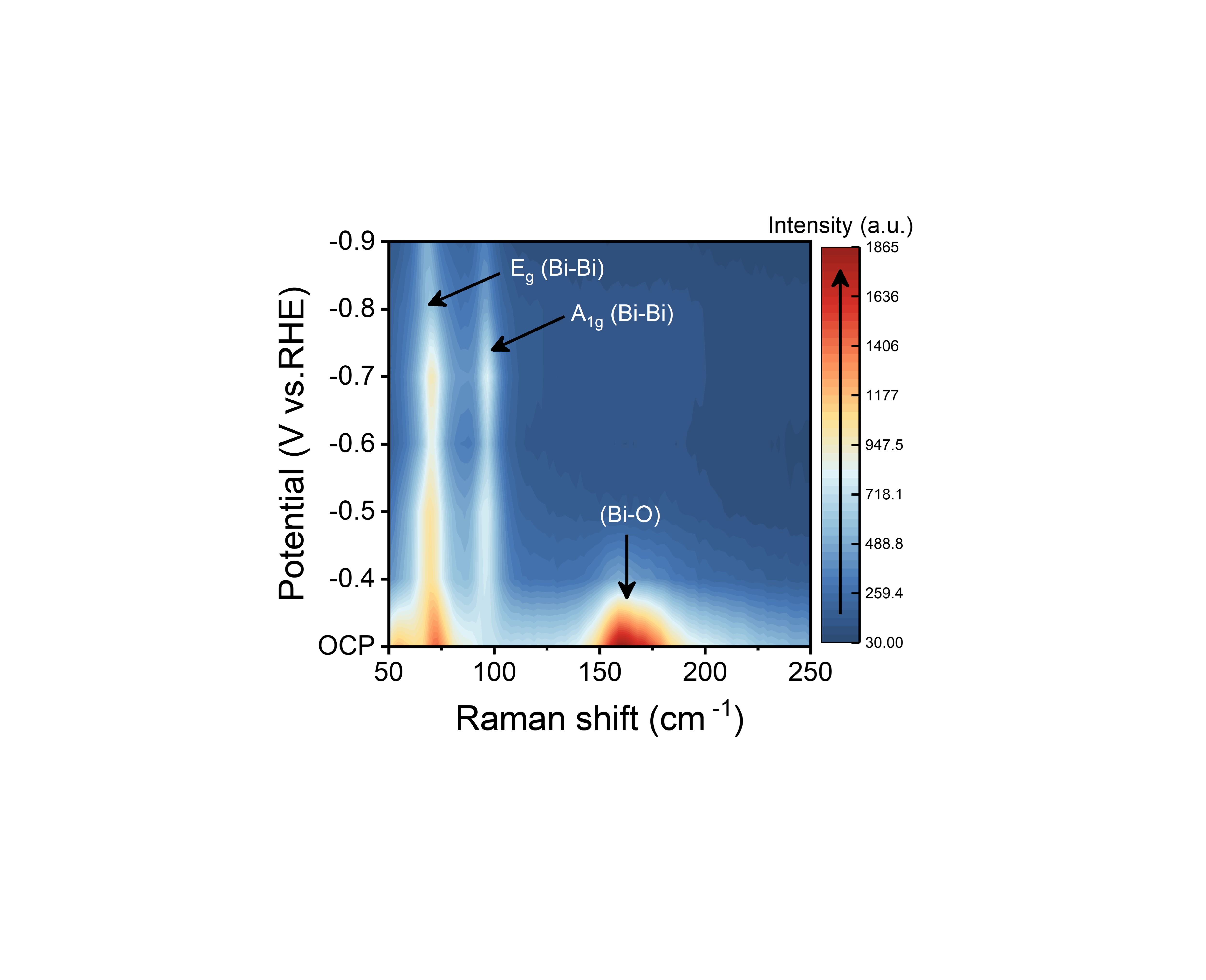


**Figure S22**. The contour map of TS-BiNs at different potentials (vs. RHE).


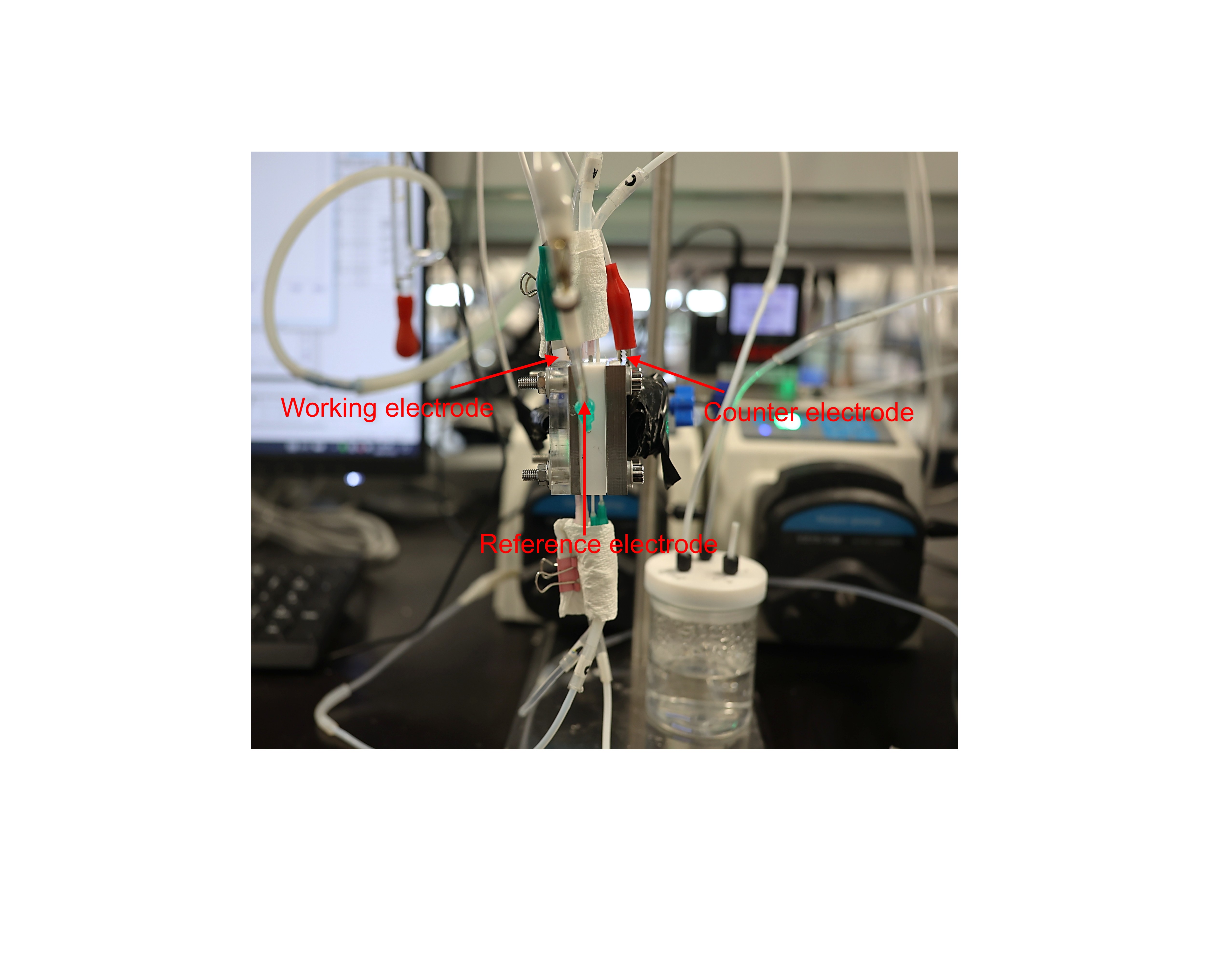


**Figure S23**. Photograph of the flow cell used for electrocatalytic CO_2_RR tests.

The catalysts were sprayed onto GDL electrodes with a mass loading of 1 mg cm^–2^ using an airbrush. A standard three-electrode flow cell with a cation-exchange membrane (CEM) separated the anolyte and catholyte. The CEM effectively prevented cross-contamination between the two solutions, ensuring the accuracy of the electrochemical measurements.


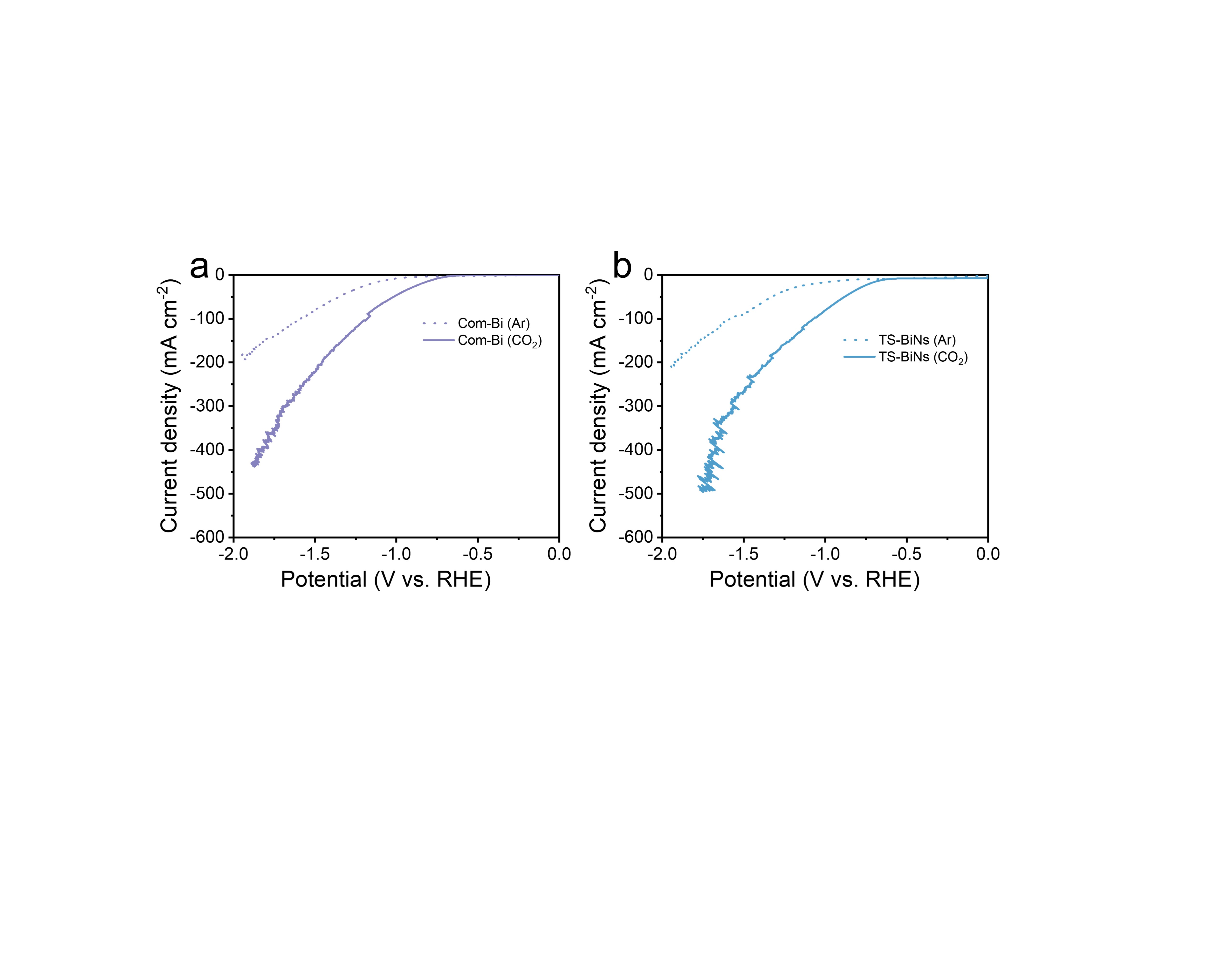


**Figure S24**. The LSV curves of (a) Com-Bi and (b) TS-BiNs in a flow cell when 1.0 M KHCO_3_ electrolyte is used under different feed gases.


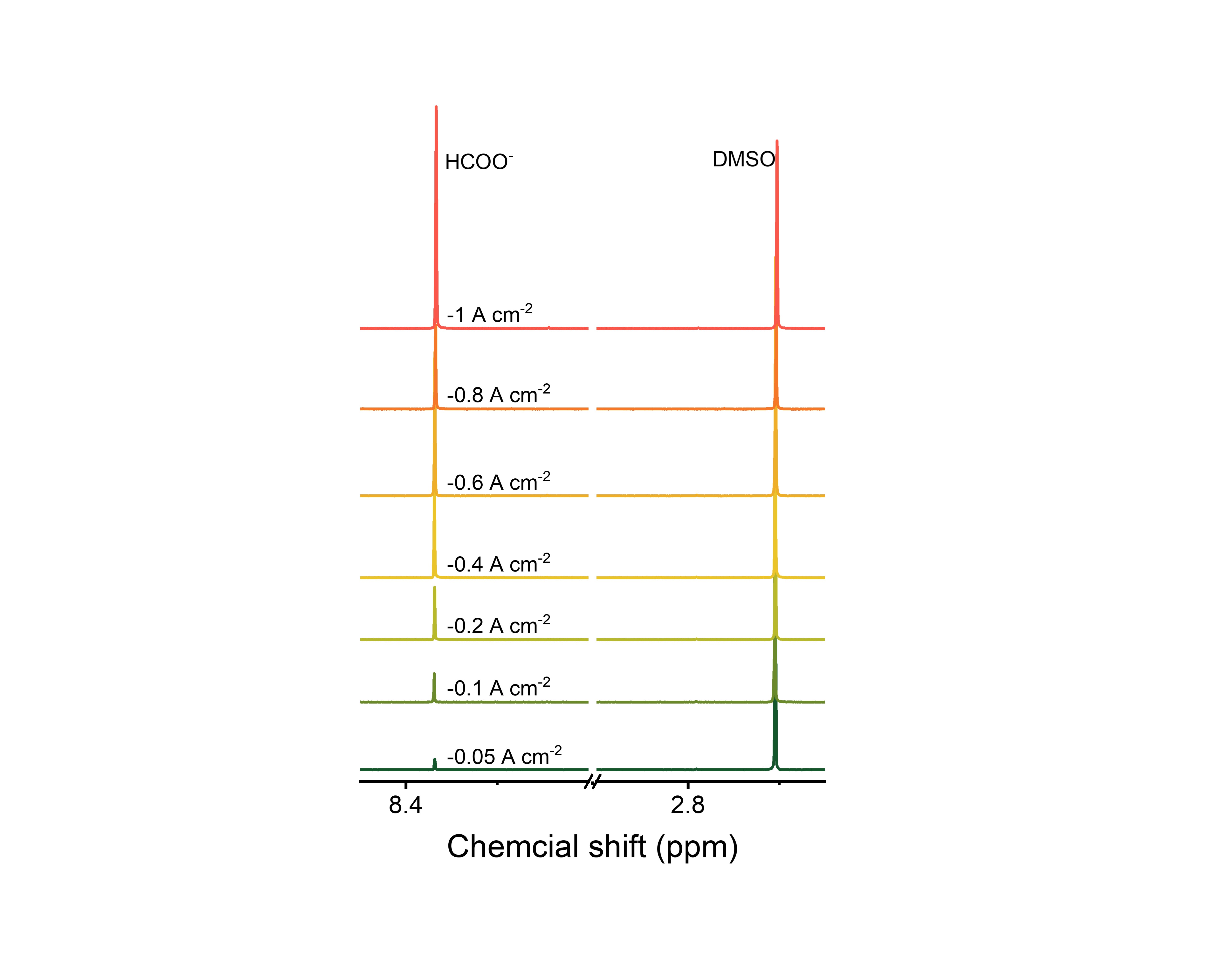


**Figure S25**. Typical ^1^H NMR spectra at different current densities for TS-BiNs catalyst. Note that the break region only shows the water peak.


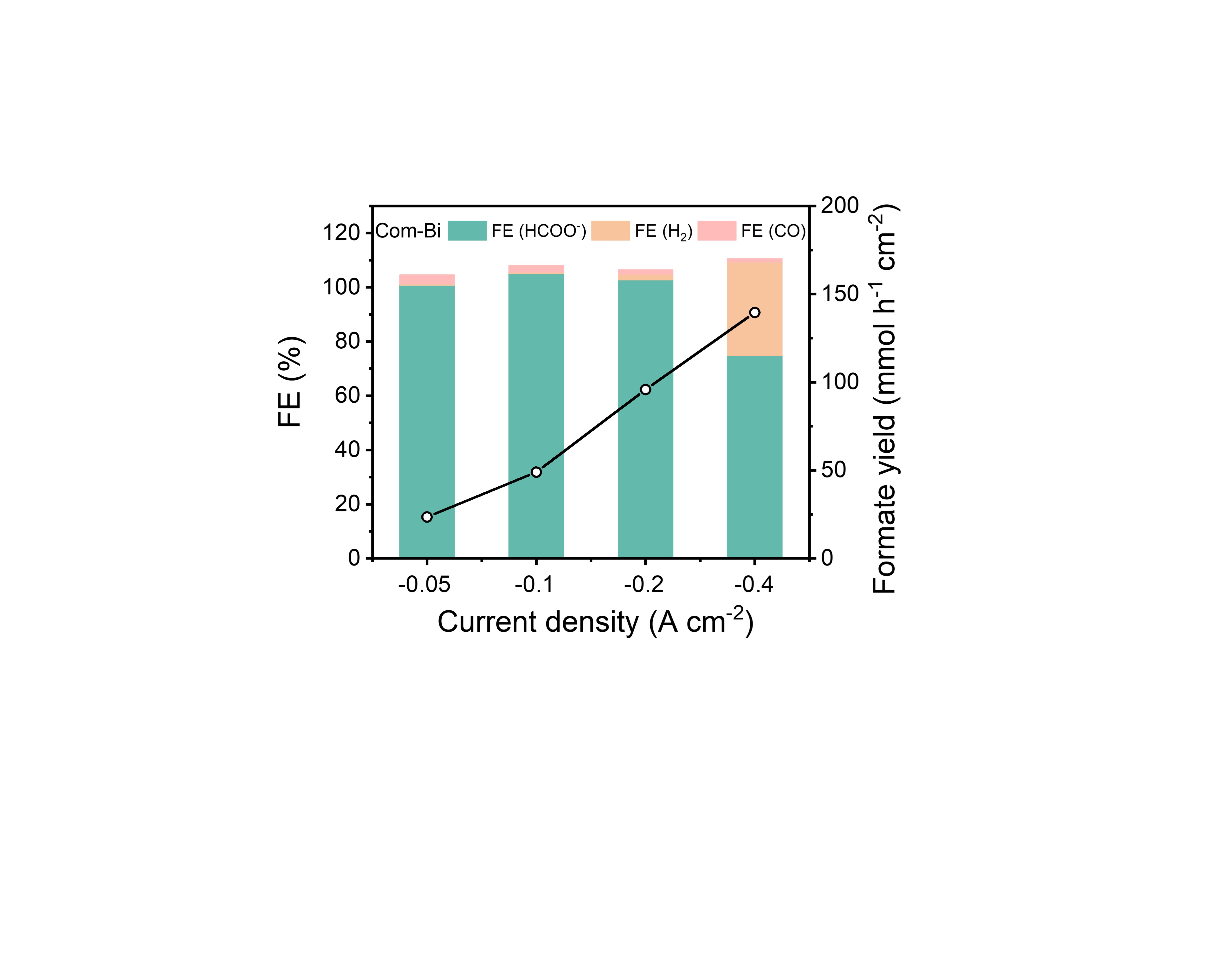


**Figure S26**. Faradaic efficiency of H_2_, CO, and HCOO^–^ on Com-Bi at different current densities.


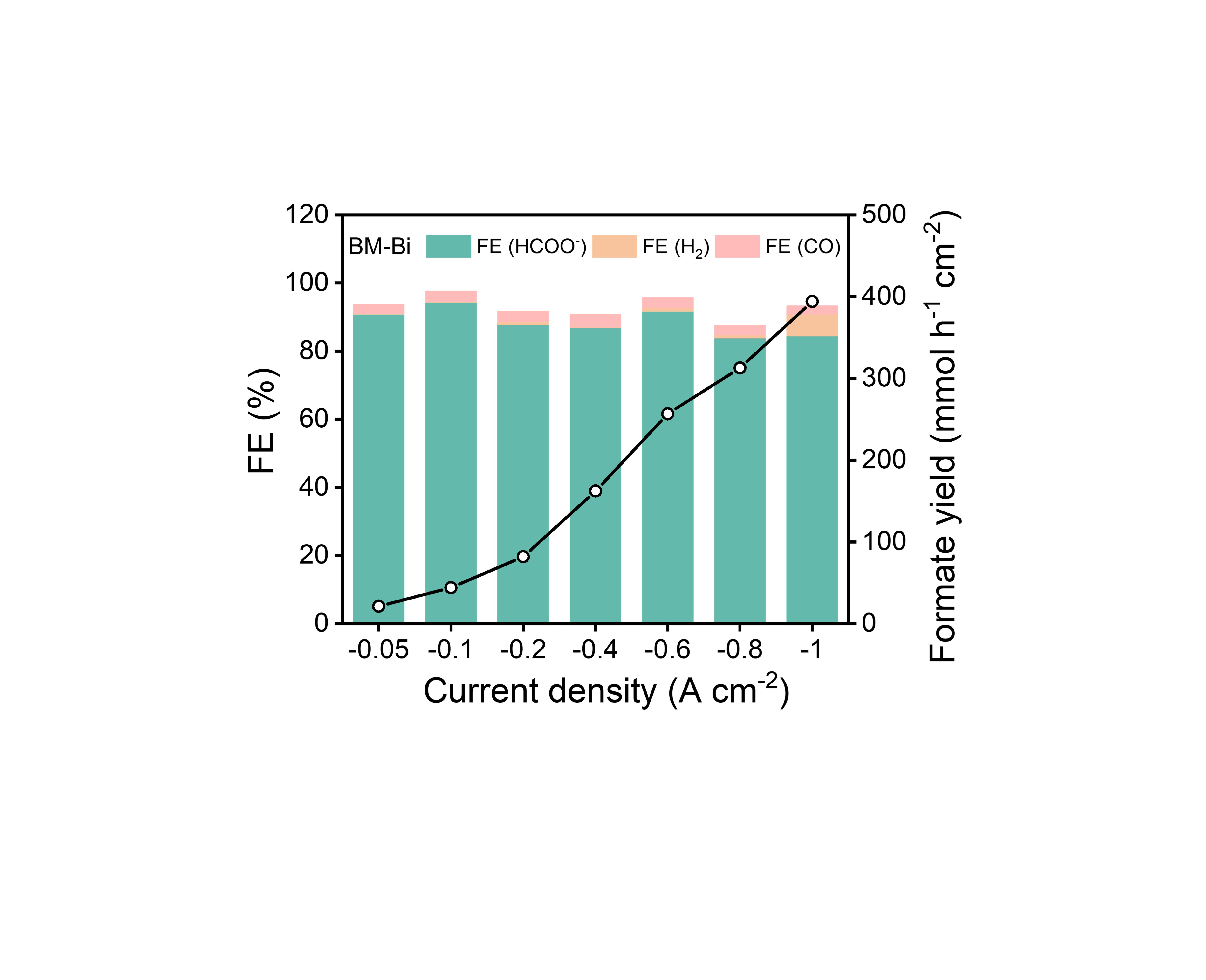


**Figure S27**. Faradaic efficiency of H_2_, CO, and HCOO^–^ on BM-Bi at different current densities.


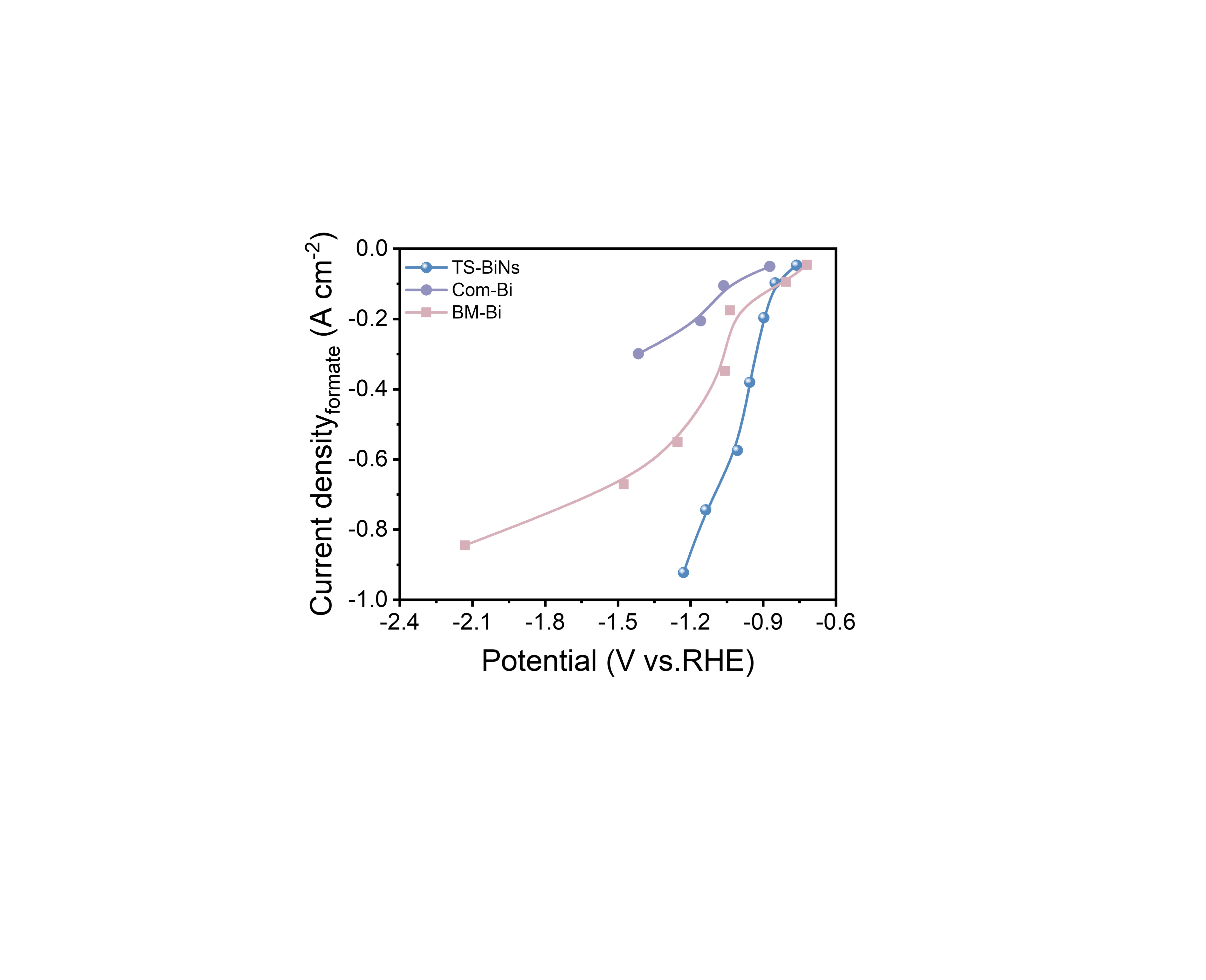


**Figure S28**. The formate partial current density curves for TS-BiNs, Com-Bi, and BM-Bi catalysts.


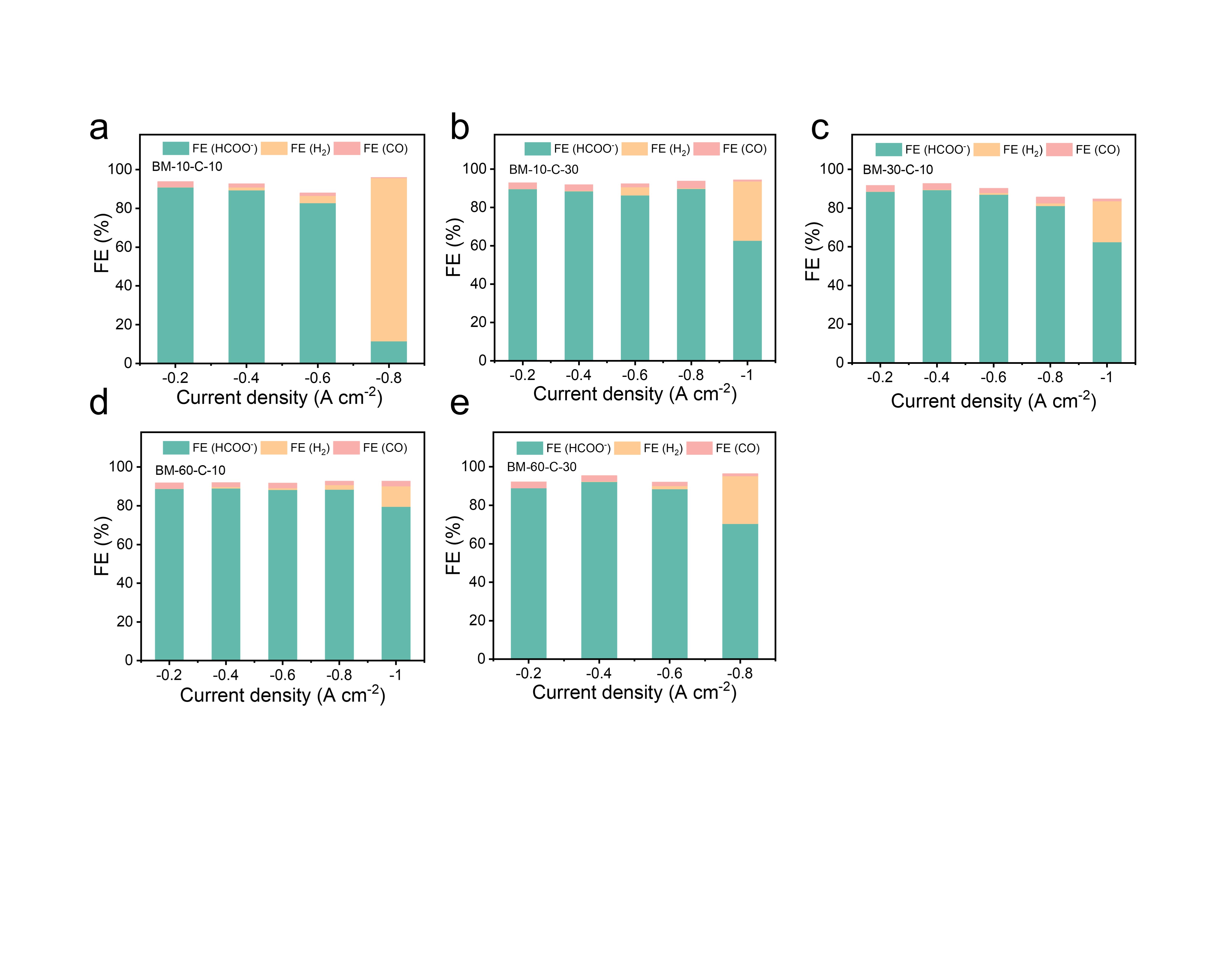


**Figure S29**. Faradaic efficiency of H_2_, CO, and HCOO^–^ for (a) BM-10 min-C-10 min, (b) BM-10 min-C-30 min, (c) BM-30 min-C-10 min, (d) BM-60 min-C-10 min, and (e) BM-60 min-C-30 min.


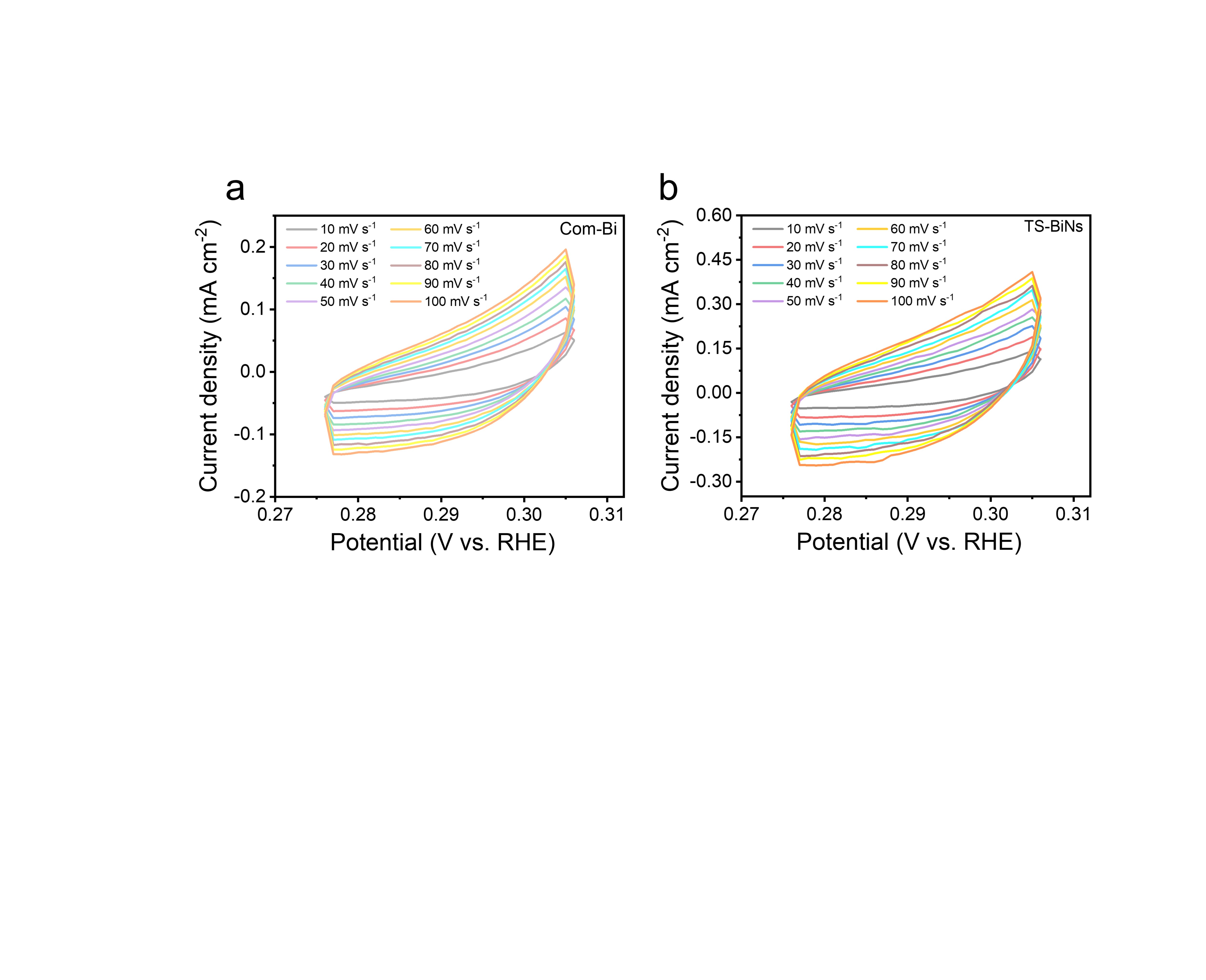


**Figure S30**. Electrochemical double layer measurements for (a) Com-Bi and (b) TS-BiNs catalysts. Cyclic voltammetry was performed in the non-reaction potential window at increasing scan rates from 10 to 100 mV s^–1^ to determine the C_dl_.

$$\mathrm{ECSA}_{Com-Bi}= \frac{0.74 mF \mathrm{cm}^{-2}}{20 \mu F \mathrm{cm}^{-2}\mathrm{per}\mathrm{cm}_{\mathrm{ECSA}}^{2}}=37.0 \mathrm{cm}_{\mathrm{ECSA}}^{2}$$

$$\mathrm{ECSA}_{TS-BiNs}= \frac{1.63 mF \mathrm{cm}^{-2}}{20 \mu F \mathrm{cm}^{-2}\mathrm{per}\mathrm{cm}_{\mathrm{ECSA}}^{2}}=81.5 \mathrm{cm}_{\mathrm{ECSA}}^{2}$$

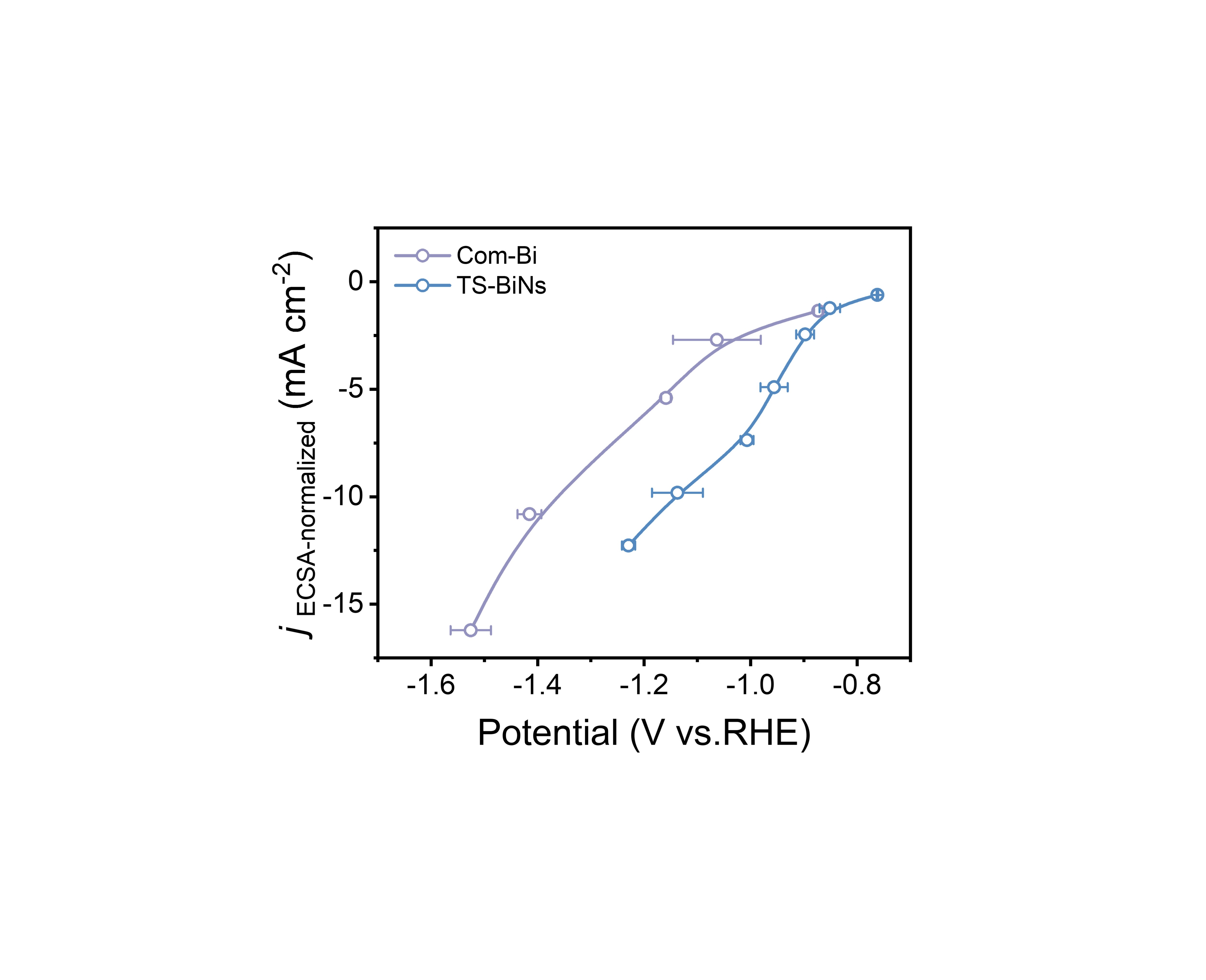


**Figure S31**. ECSA-normalized I-V curves for TS-BiNs and Com-Bi.


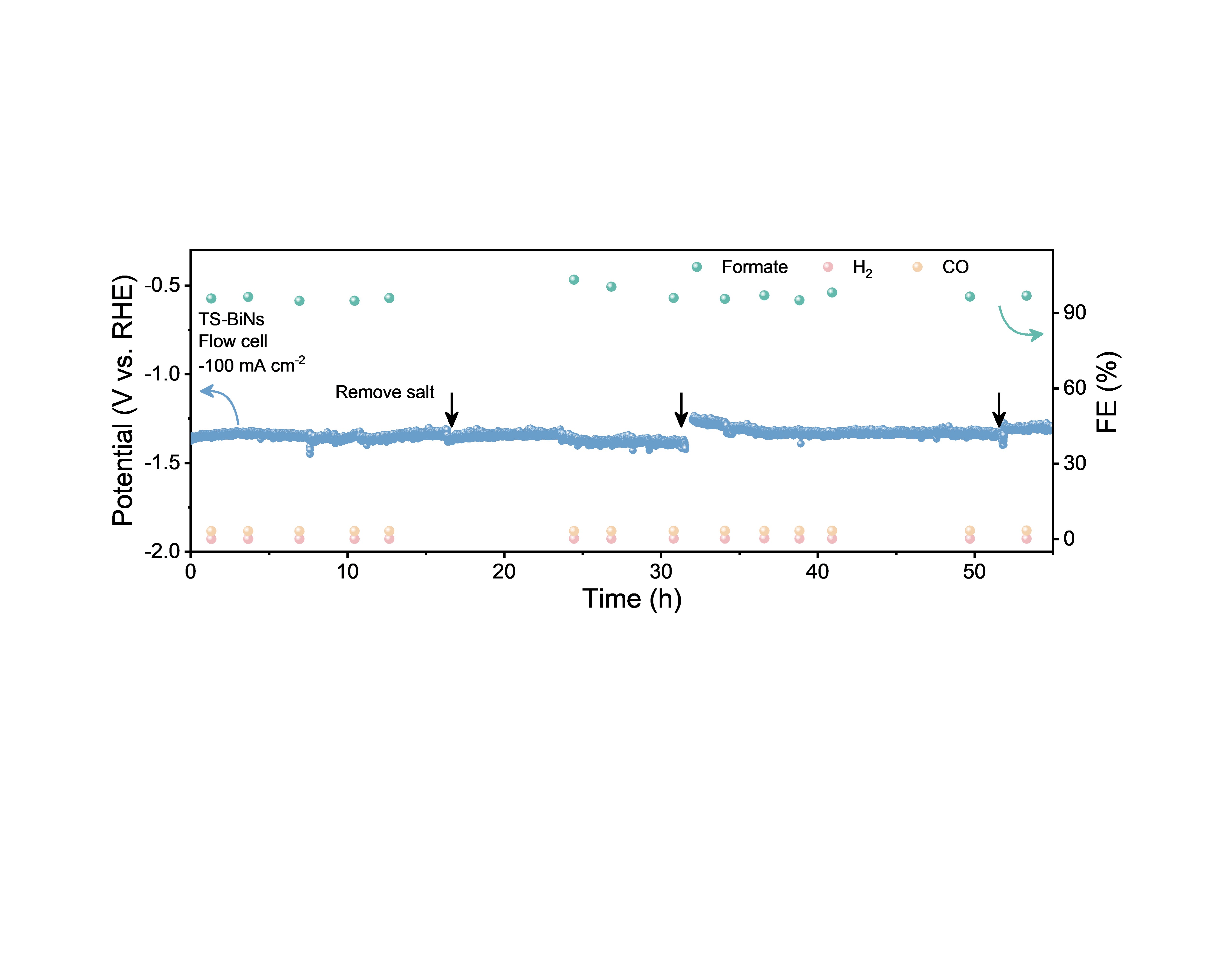


**Figure S32**. Preliminary long-time stability at a current density of 100 mA cm^–2^ in a flow cell (without iR correction).

Our preliminary stability test results show that the TS-BiNs can operate stably for more than 50 h at a current density of –100 mA cm^–2^ in a 1.0 M KHCO_3_ electrolyte, with the FE_HCOO_^–^ maintained above 96% and the by-products of H_2_ and CO remaining at extremely low levels.


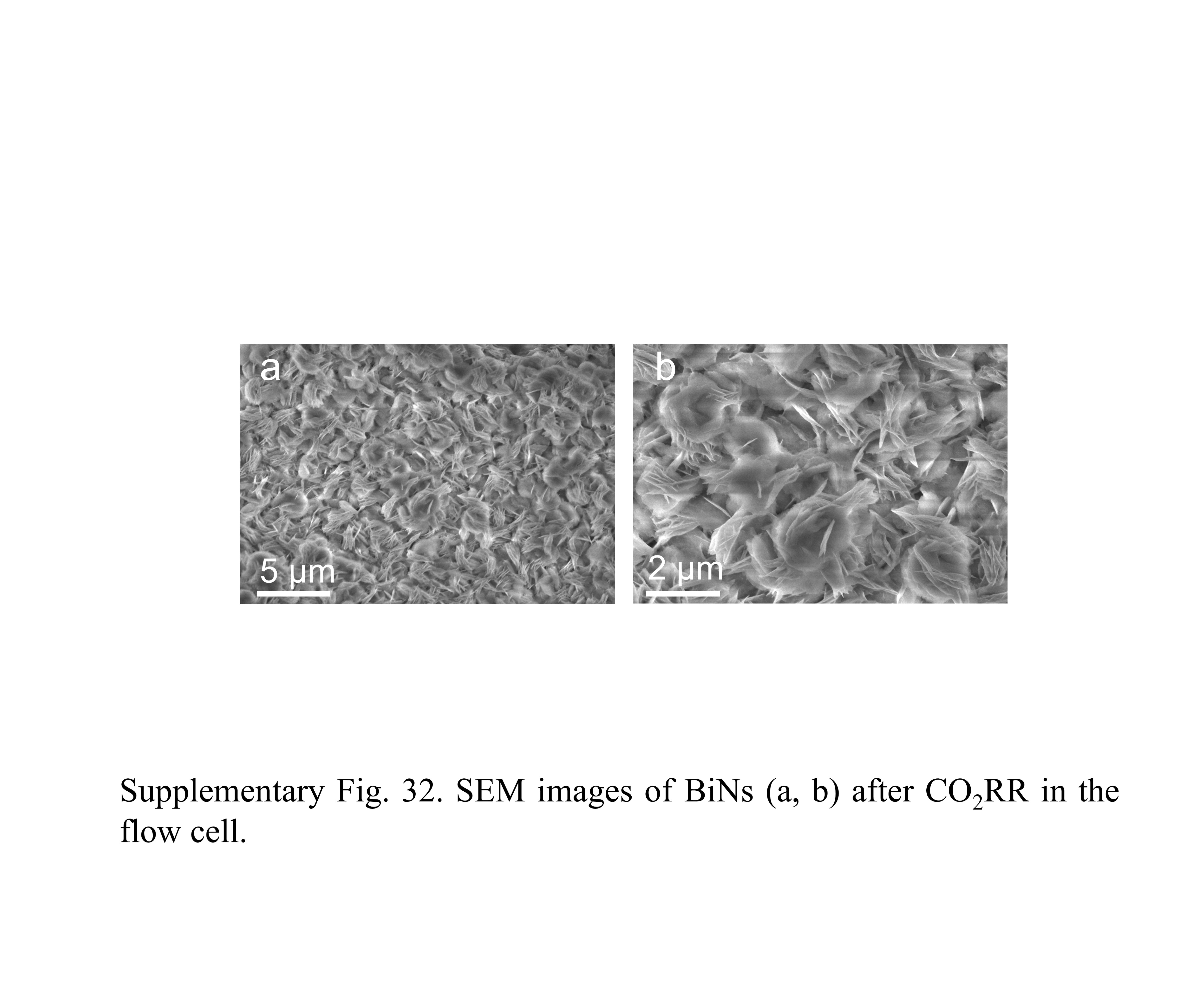


**Figure S33**. (a, b) SEM images of TS-BiNs after CO_2_RR in a flow cell.


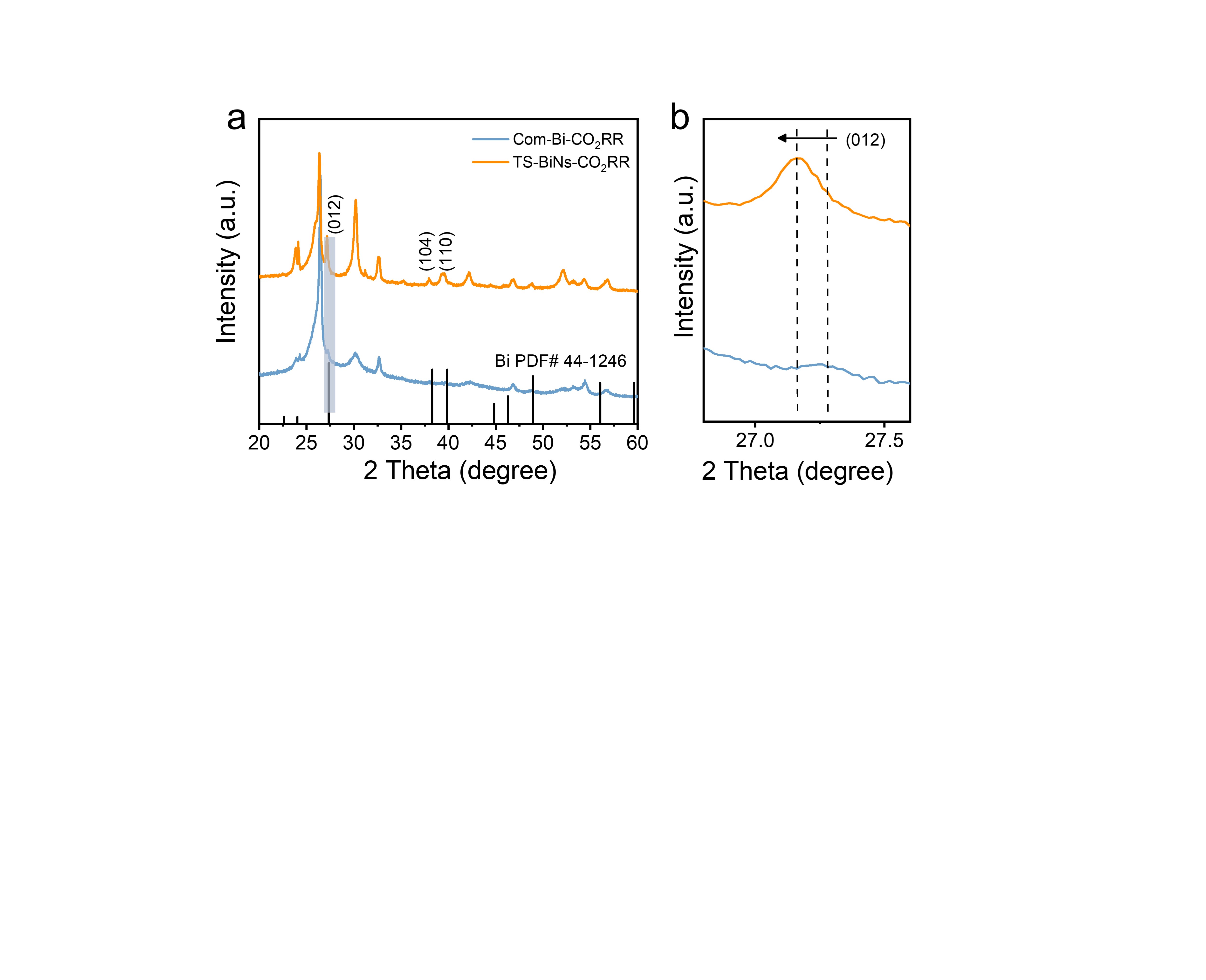


**Figure S34**. (a, b) XRD patterns of two kinds of pre-catalysts after CO_2_RR and the enlarged image of the diffraction peak at the (012) crystal plane.

The weak signals of Bi oxides observed in the XRD pattern were attributed to the oxidation of active Bi in the air atmosphere during the XRD test.


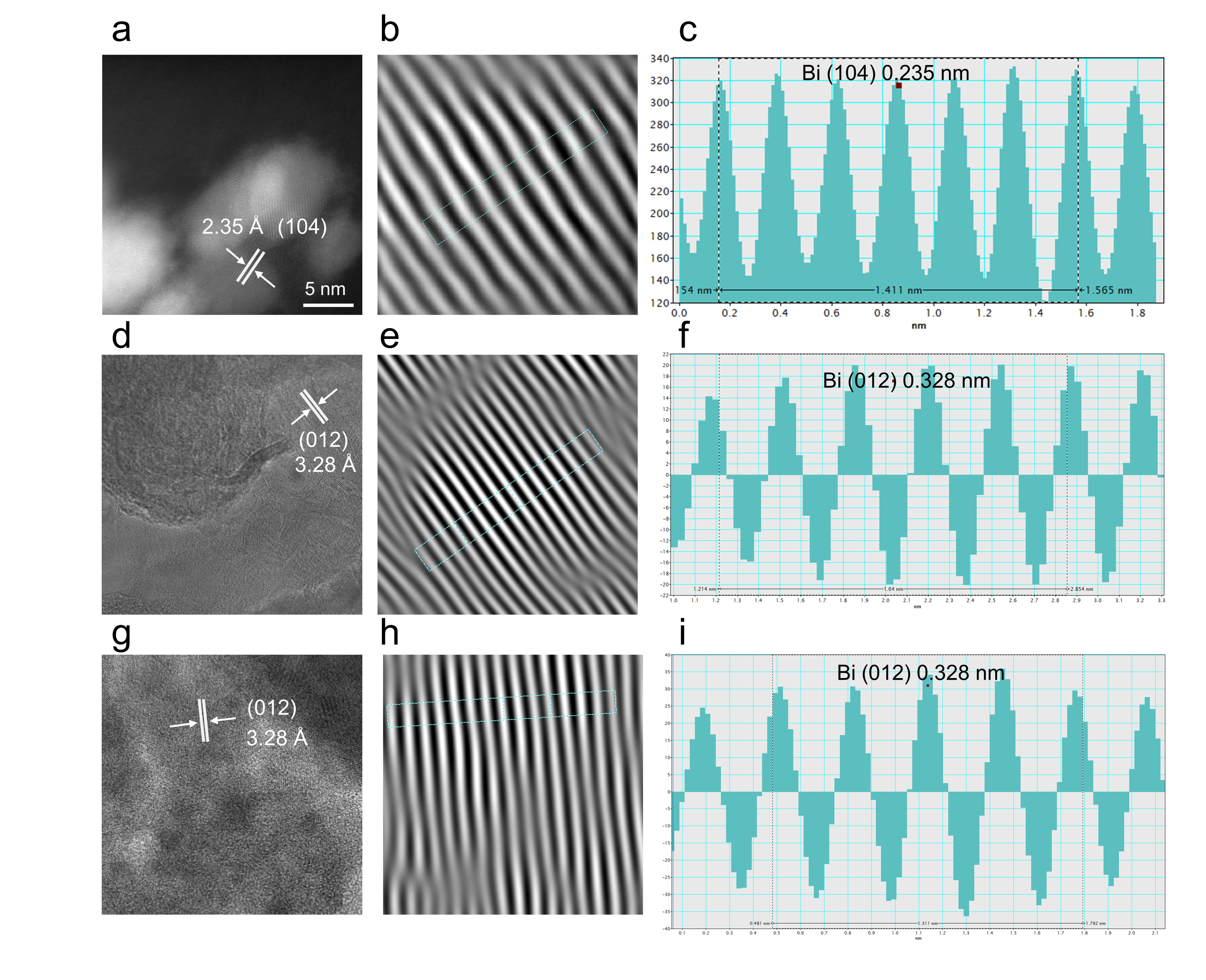


**Figure S35**. Structural characterization of Com-Bi after the CO_2_RR. (a, d, g) The HADDF-STEM/TEM images of Com-Bi after CO_2_RR, (b, e, h) the zoom-in images. The atomic distance of the (c) Bi (104) facet and (f, i) Bi (012) facet in different regions.


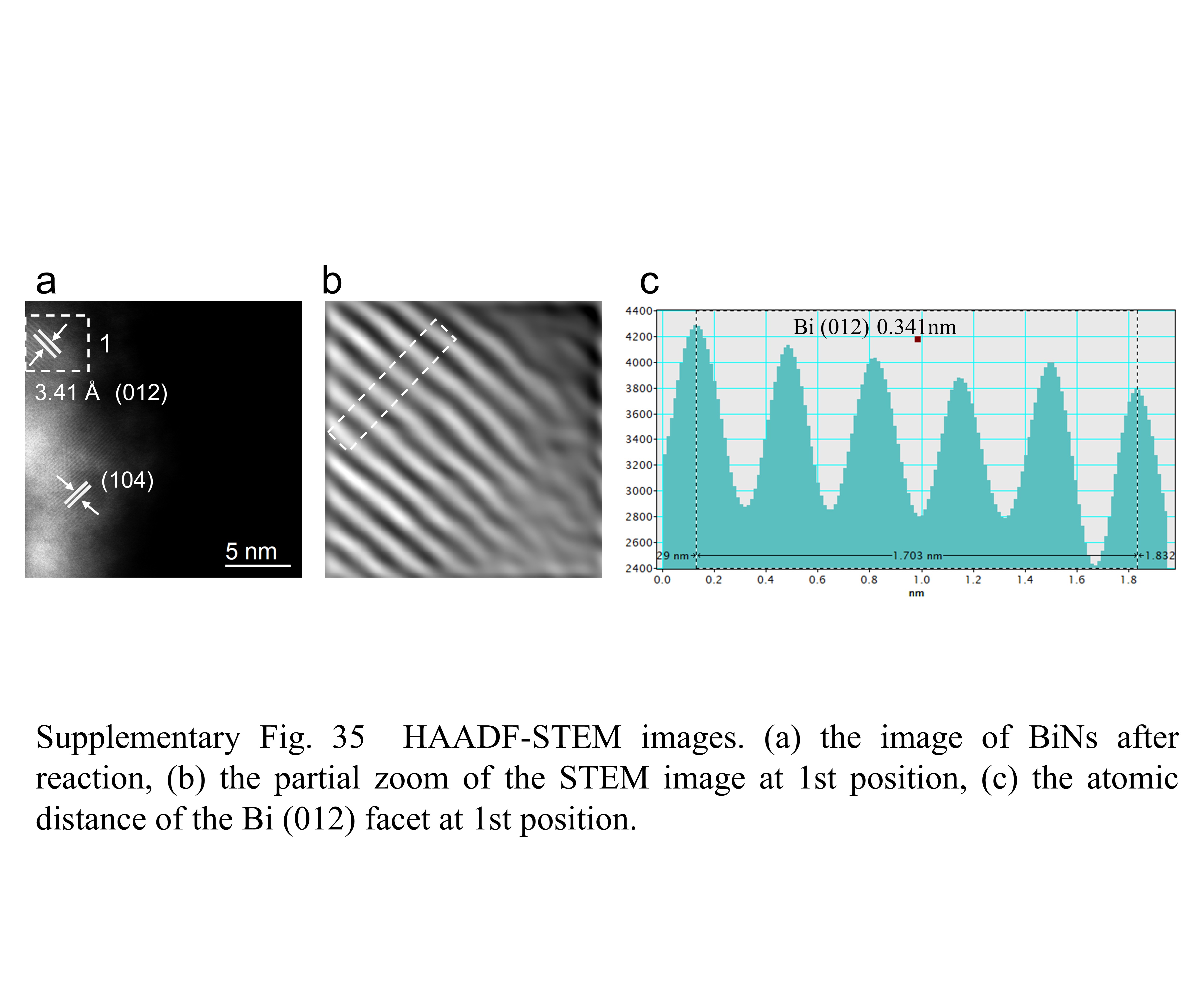


**Figure S36**. Structural characterization of TS-BiNs after the CO_2_RR. (a) The HADDF-STEM image of TS-BiNs after CO_2_RR, (b) the zoom-in image at region #1, (c) the atomic distance of the Bi (012) facet at region #1. We calculate strain(s) through the formula: s = (d_2_ − d_1_) / d_1_ ∗ 100%, where d_2_ denotes the actual interplanar spacing calculated by HAADF-STEM, and d_1_ represents the theoretical interplanar spacing.

$$S_{TS-BiNs}= \frac{3.41 Å-3.28 Å}{3.28 Å}*100\%\approx4\%$$

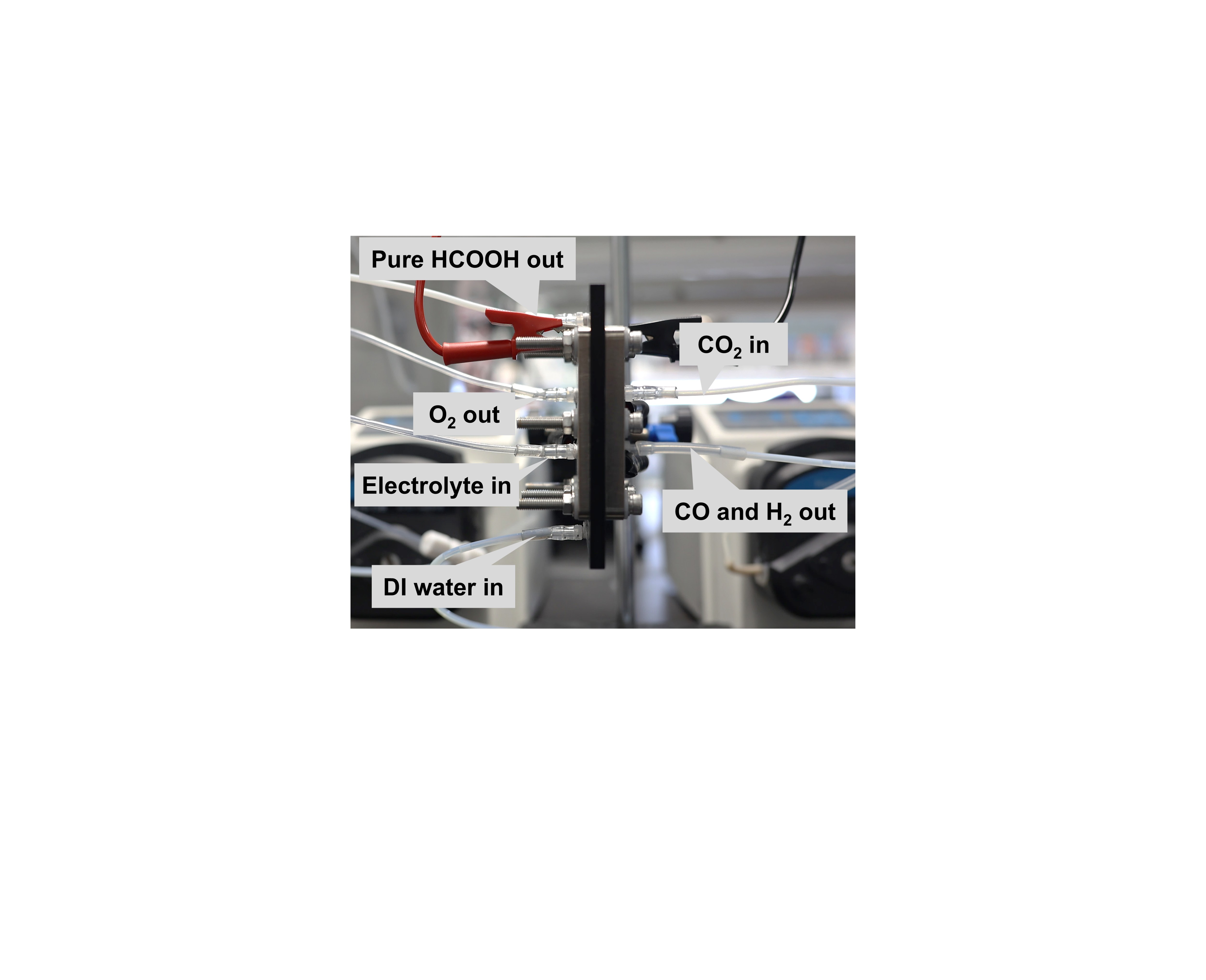


**Figure S37**. Photograph of the CO_2_RR in the SSE reactor for continuous production of pure HCOOH solution.


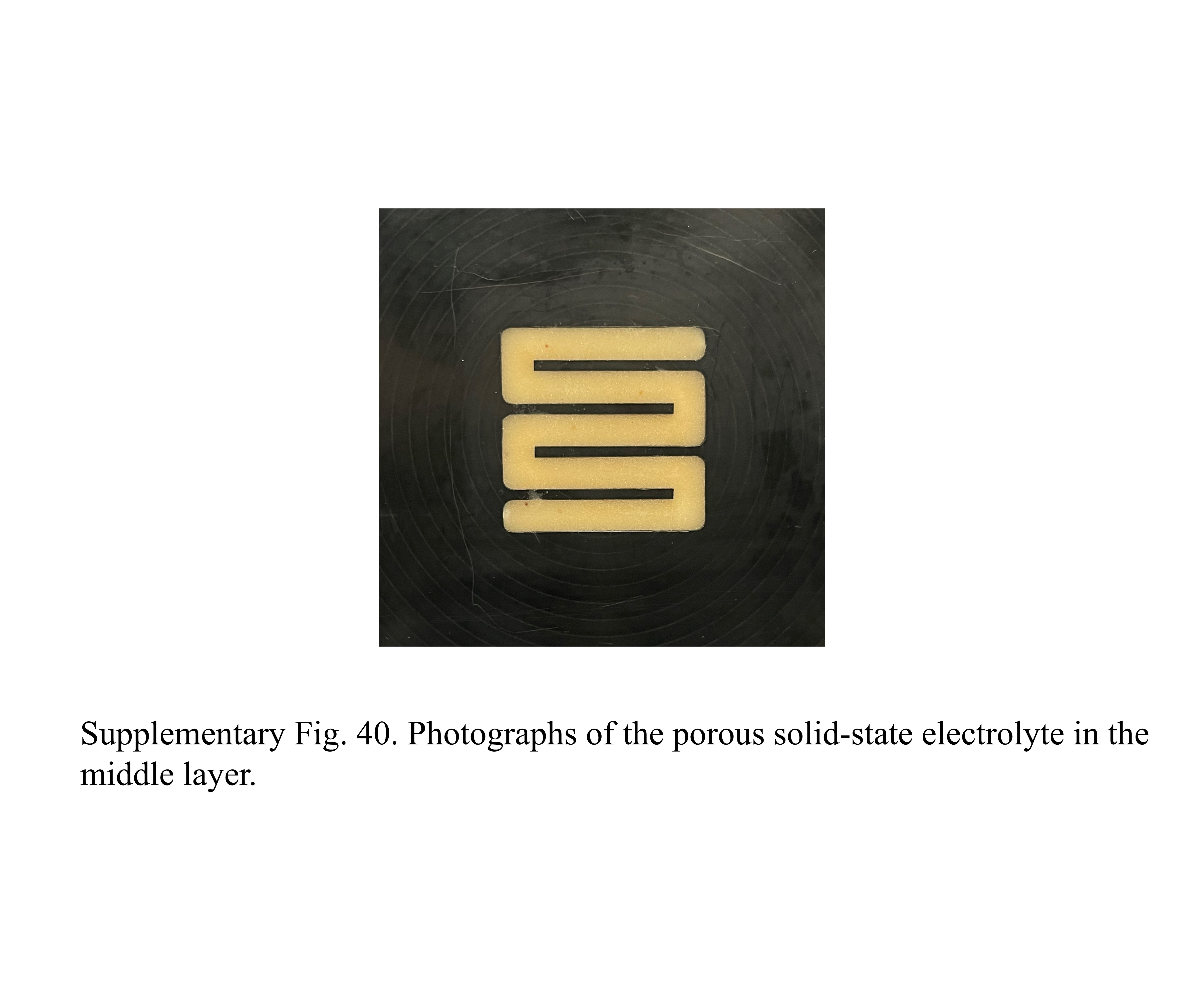


**Figure S38**. Photographs of the porous solid-state electrolyte in the middle layer.

During the CO_2_RR, at the catalyst modified cathode, the generated negatively charged HCOO^–^ species are electrostatically driven into the central chamber of the SSE reactor under the influence of an applied electric field. Concurrently, at the anode, the highly stable and active IrO_2_ catalyst facilitates the oxygen evolution reaction (OER) of water, releasing protons (H^+^). These H^+^ ions migrate through the electrolyte layer to neutralize the charge of HCOO^–^. The subsequent electrochemical coupling of H^+^ and HCOO^–^ leads to the formation of HCOOH within the SSE layer. A pure HCOOH solution can then be easily collected by gently flushing the SSE region with deionized water, yielding a product stream free from any by-products.


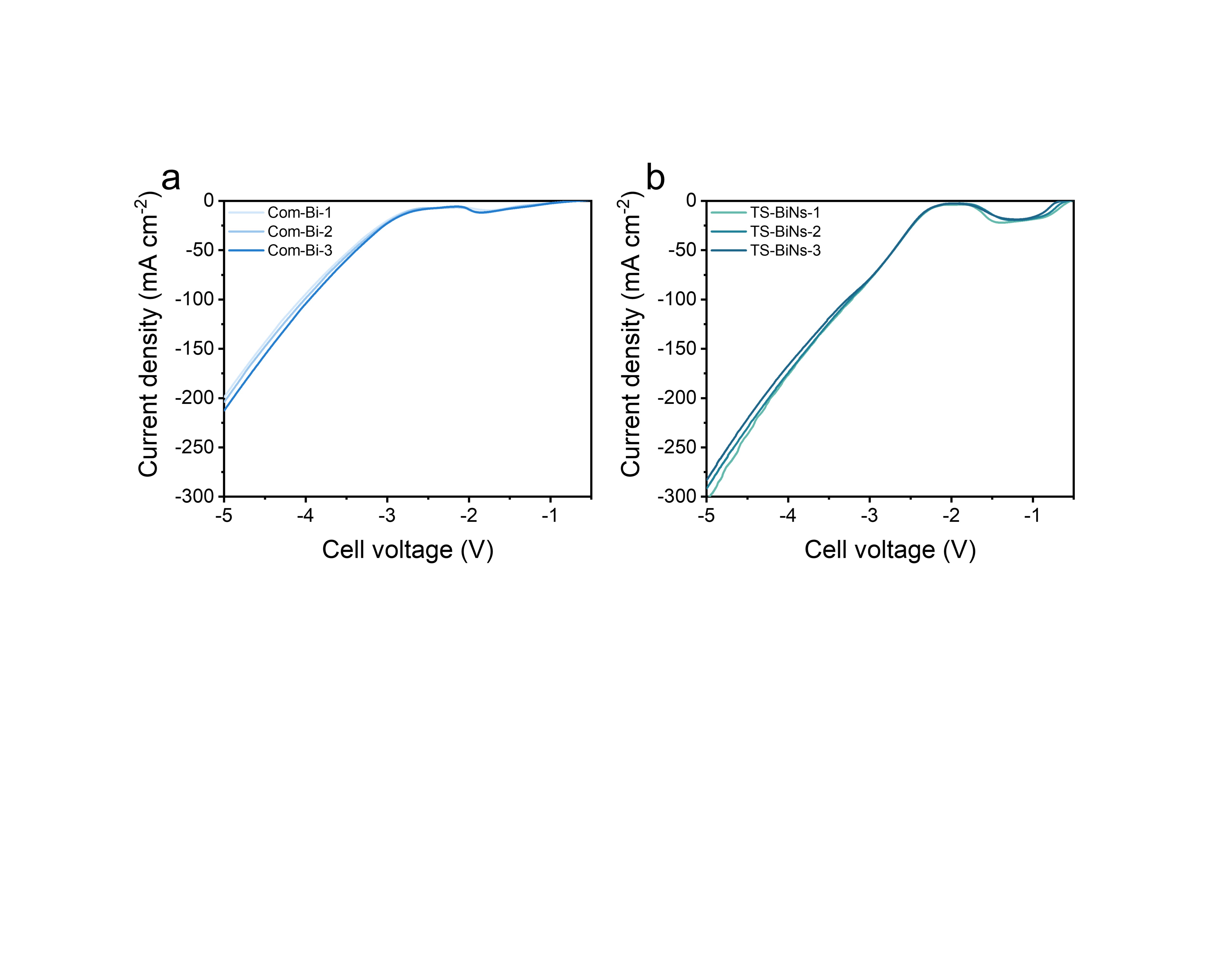


**Figure S39**. LSV curve for (a) Com-Bi and (b) TS-BiNs catalysts in the SSE reactor (reaction area of 4 cm^2^; without iR correction).


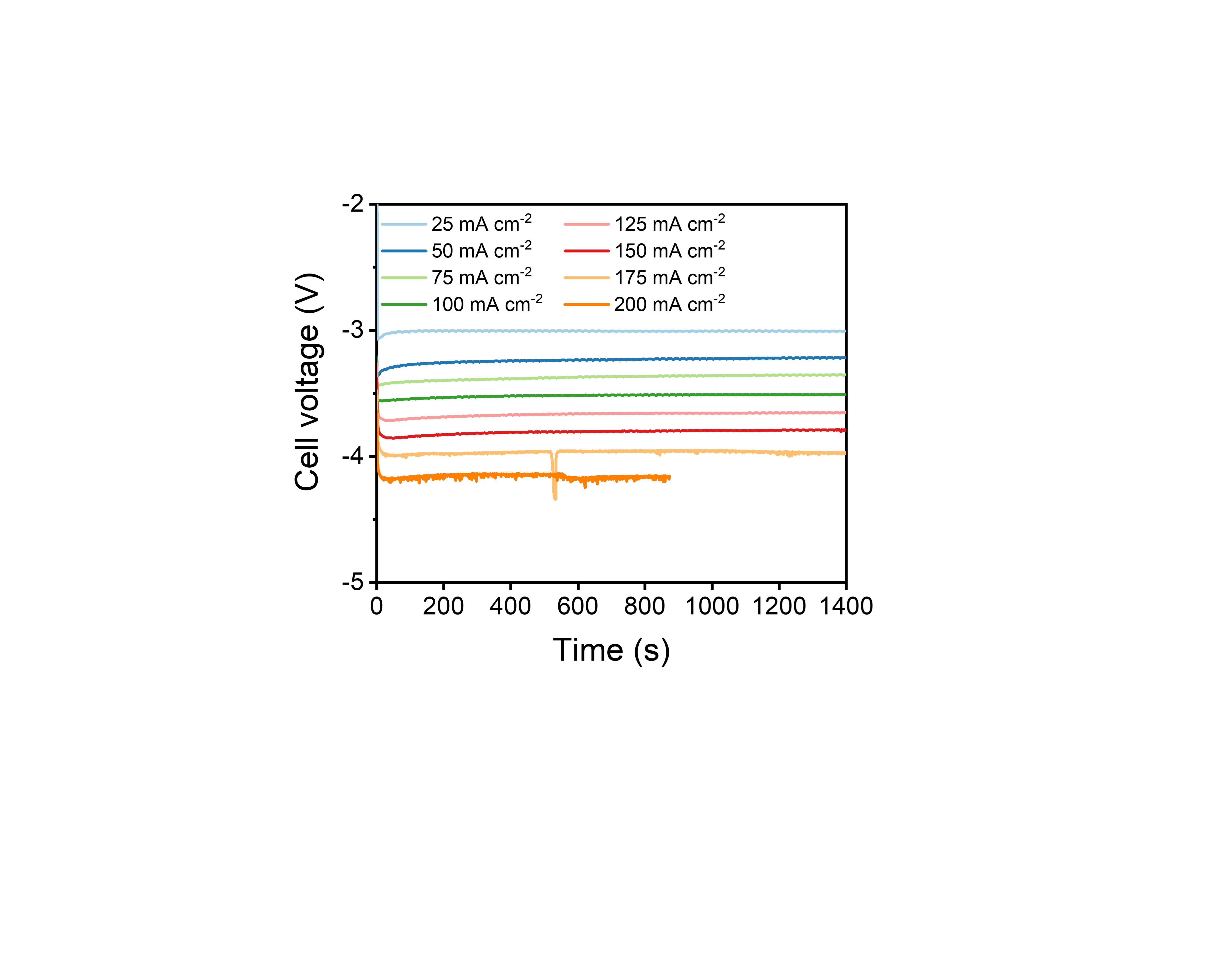


**Figure S40**. Cell voltage at different cell current densities (reaction area of 4 cm^2^) for the TS-BiNs catalyst in the SSE reactor.


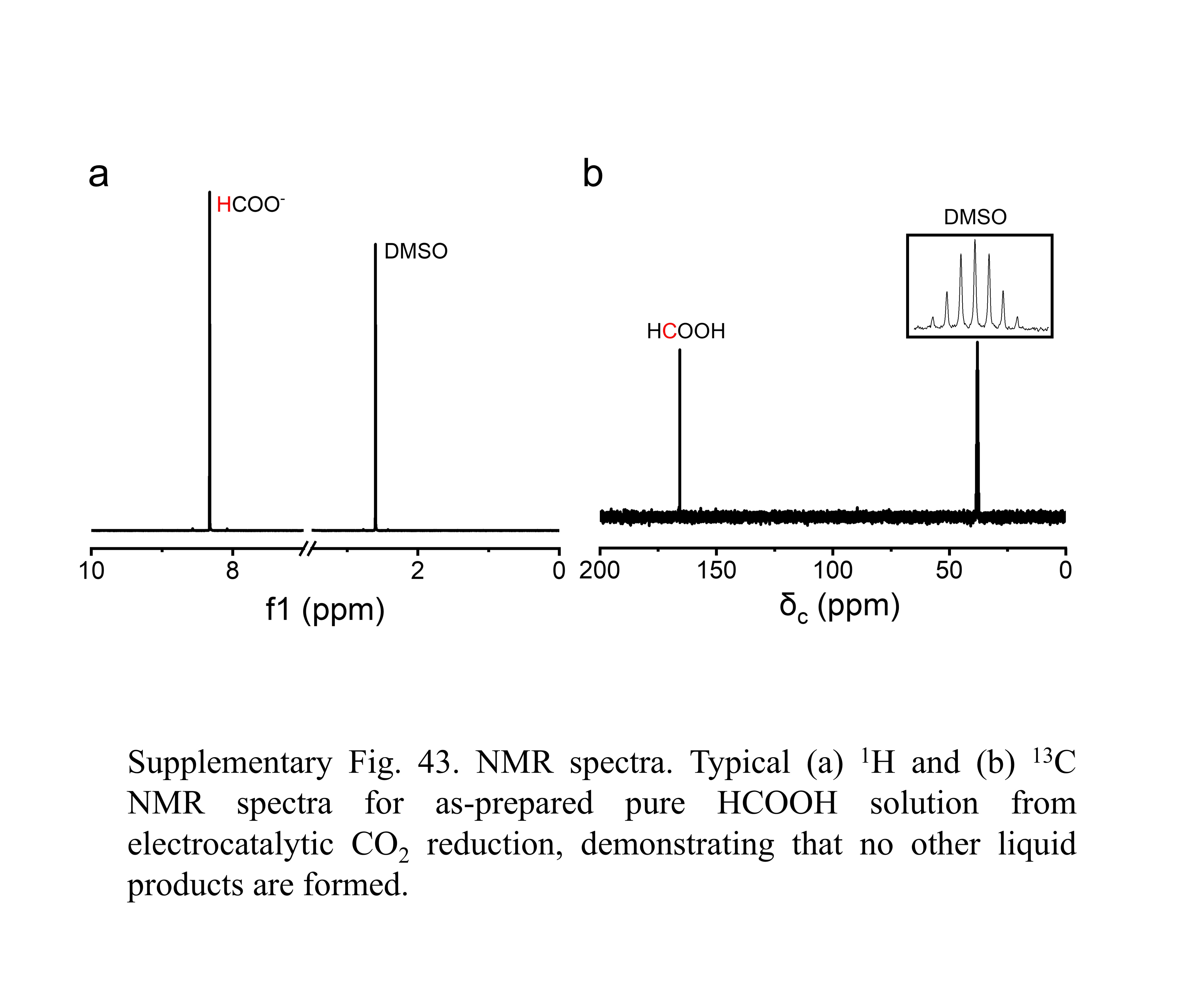


**Figure S41**. NMR spectra. Typical (a) ^1^H and (b) ^13^C NMR spectra for as-prepared pure HCOOH solution from electrocatalytic CO_2_ reduction, demonstrating that no other liquid products were formed.


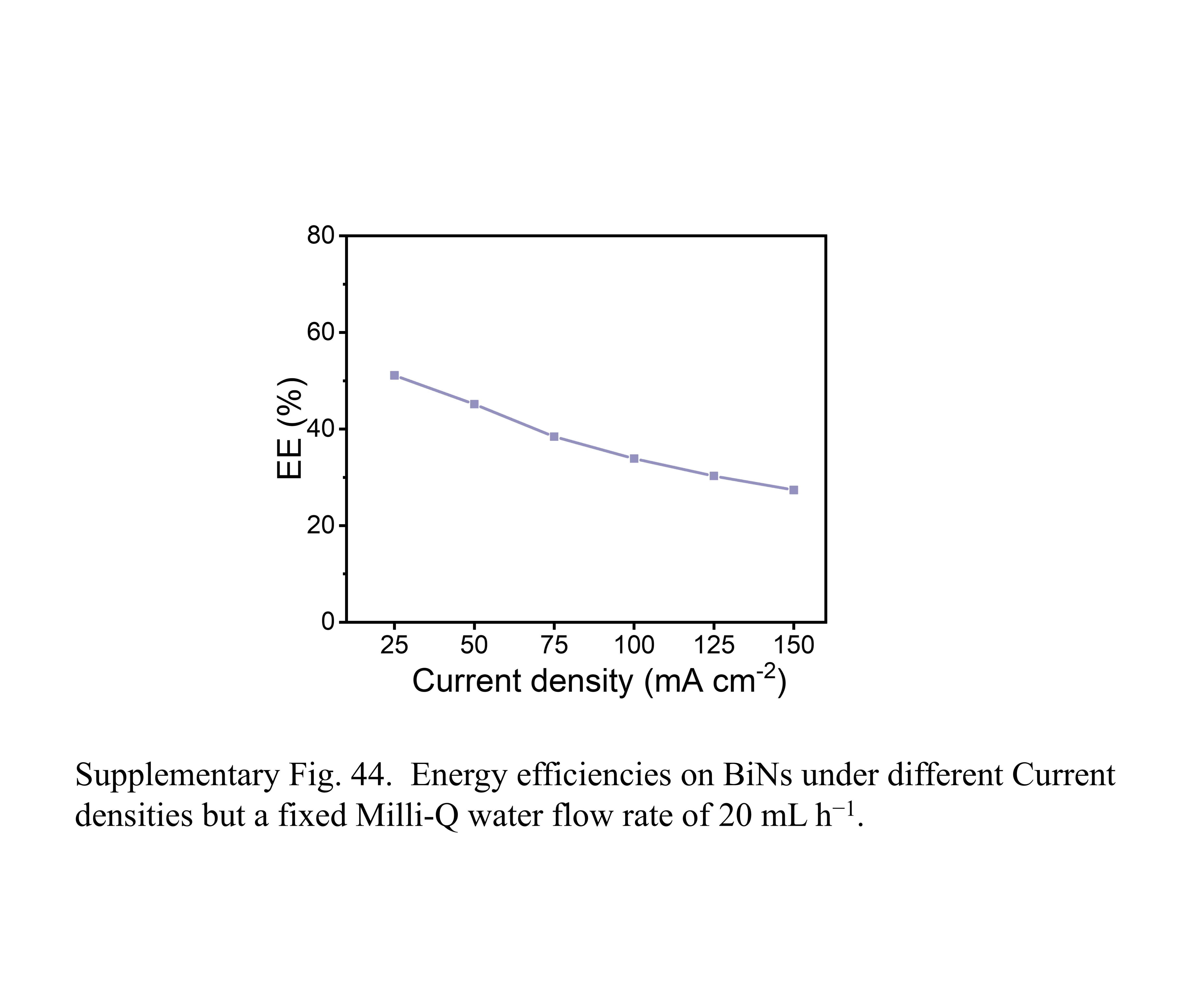


**Figure S42**. Energy efficiencies of the electrochemical CO_2_RR tests in the SSE reactor using TS-BiNs catalyst under different current densities but a fixed deionized water flow rate of 20 mL h^−1^.


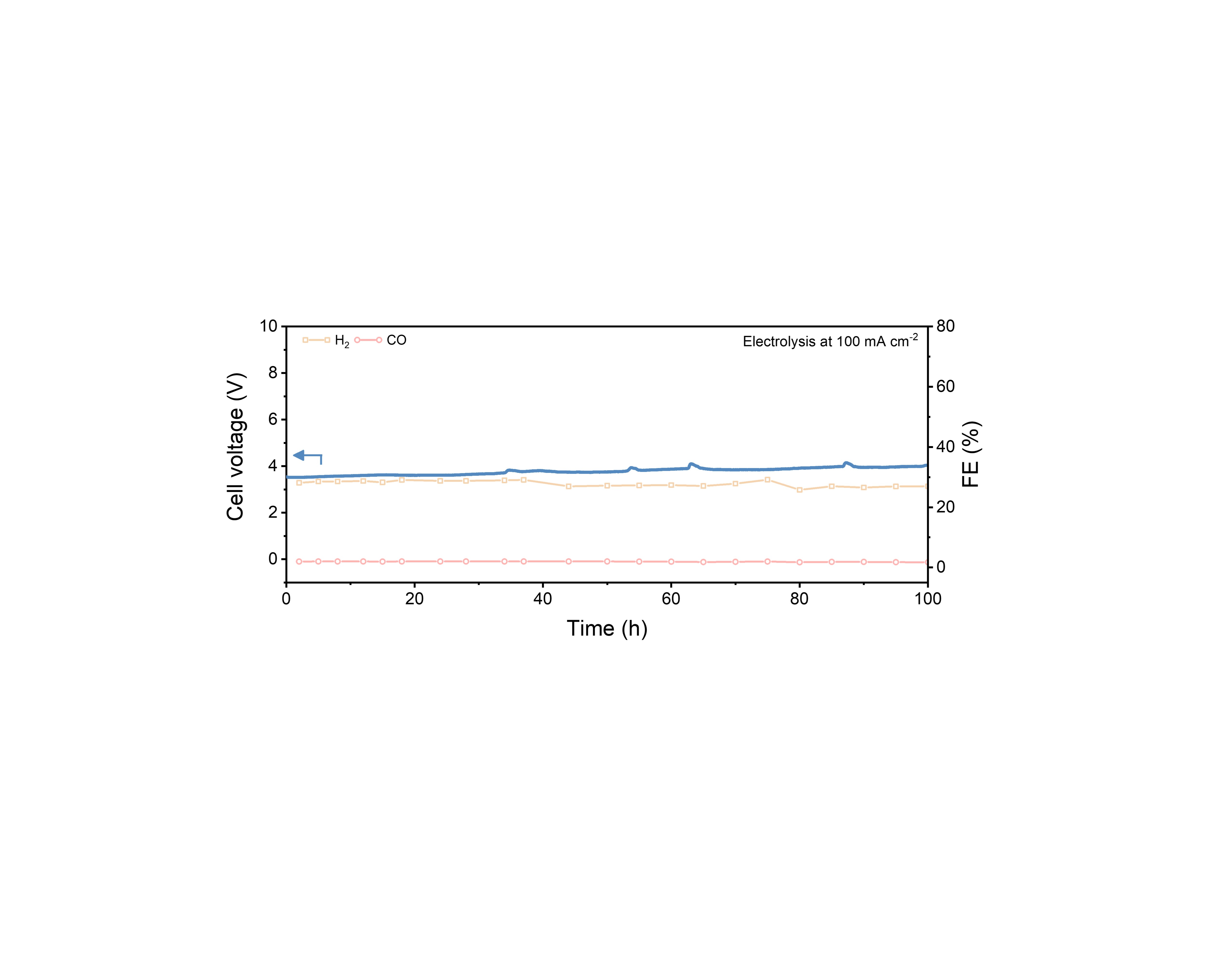


**Figure S43**. Long-time stability test and the CO and H_2_ FE of TS-BiNs for continuously reducing CO_2_ to HCOOH solution at 100 mA cm^−2^ (reaction area of 4 cm^2^) in the SSE of flow rate at 30 mL h^–1^ (without iR correction).


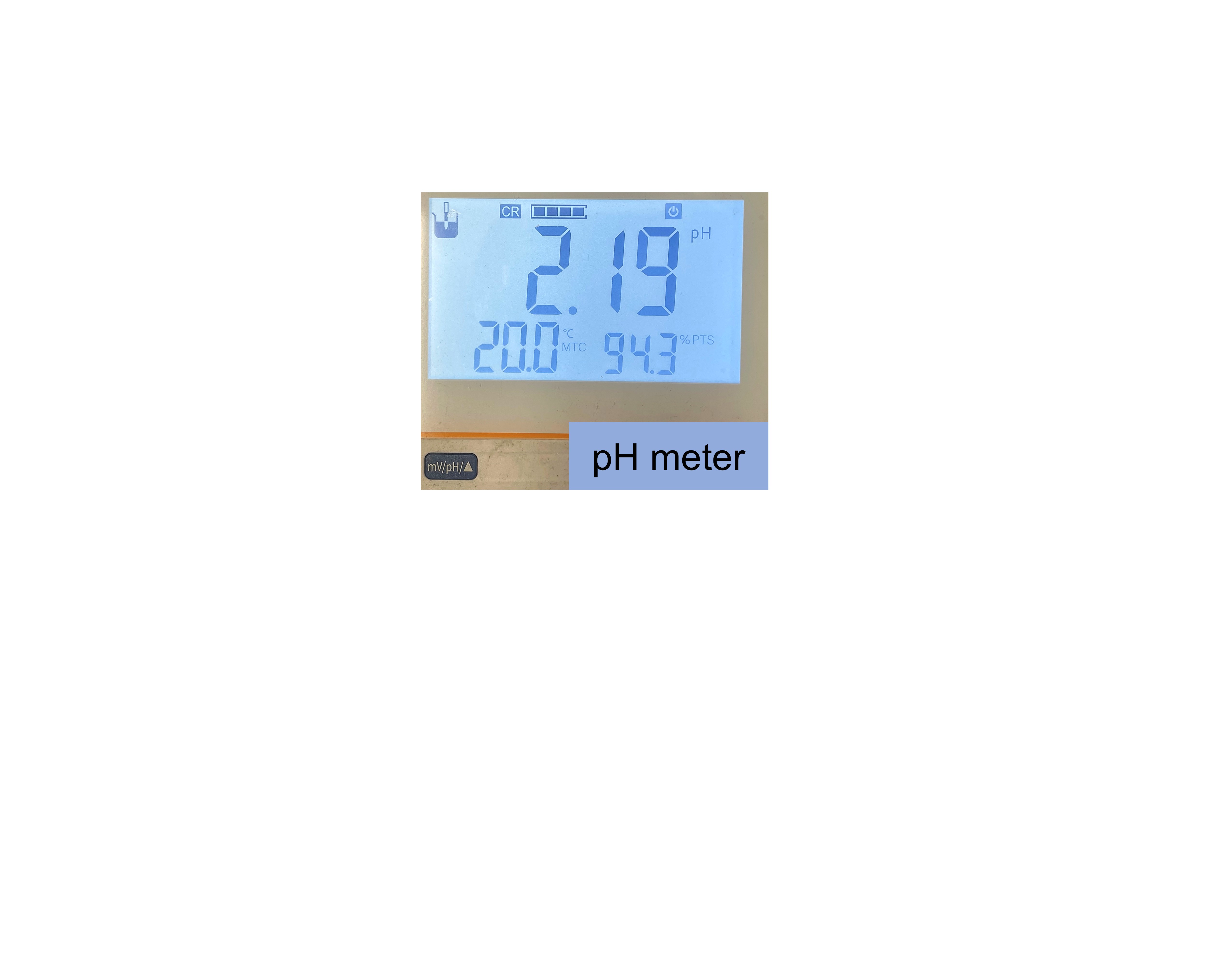


**Figure S44**. Photograph of pH testing of the 0.18 M pure formic acid solution (pH ≈ 2.2) obtained from long-time electrolysis.


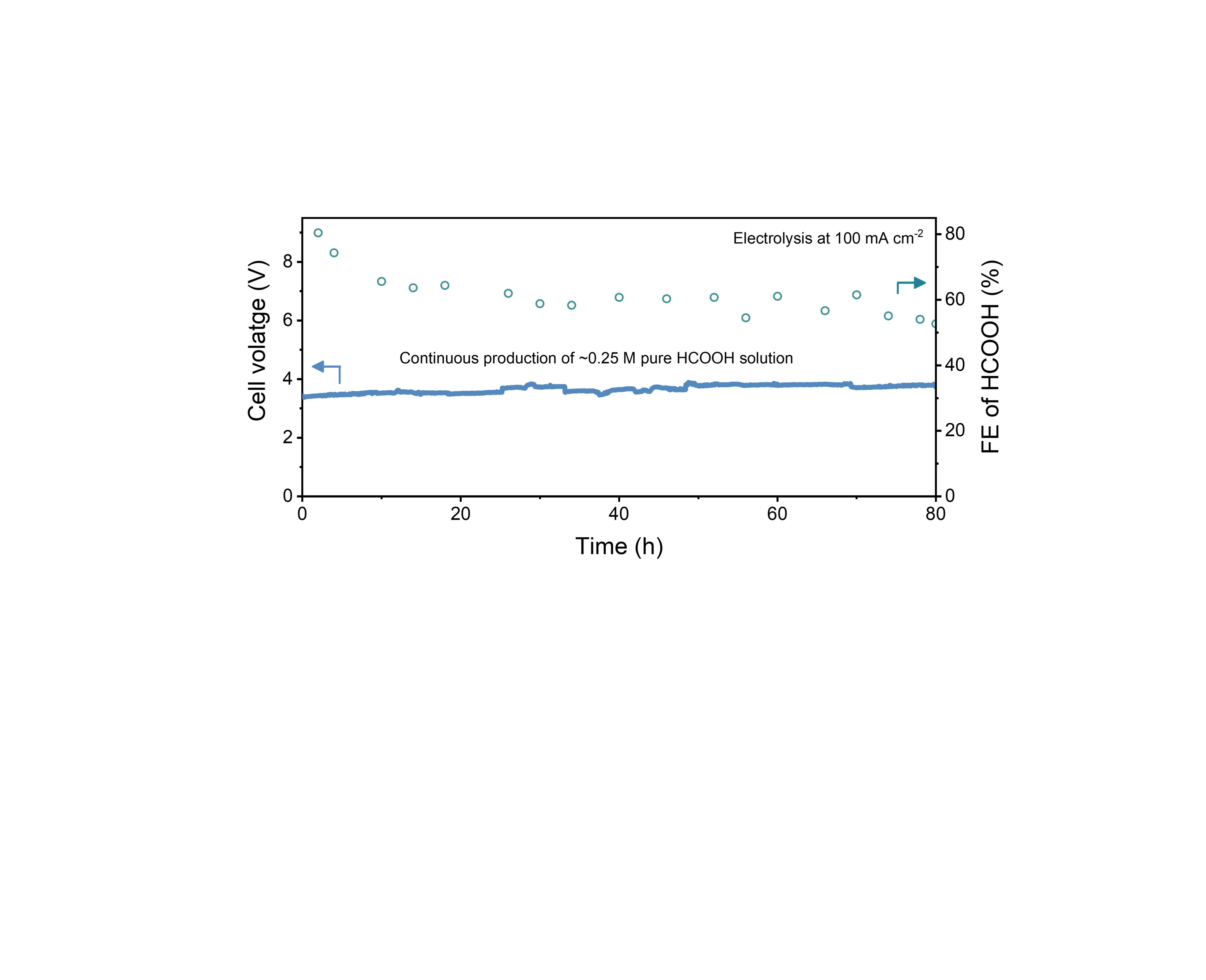


**Figure S45**. Long-time production of pure HCOOH over TS-BiNs at 100 mA cm^–2^ (reaction area of 4 cm^2^) in the SSE reactor of flow rate at 25 mL h^–1^ (without iR correction).


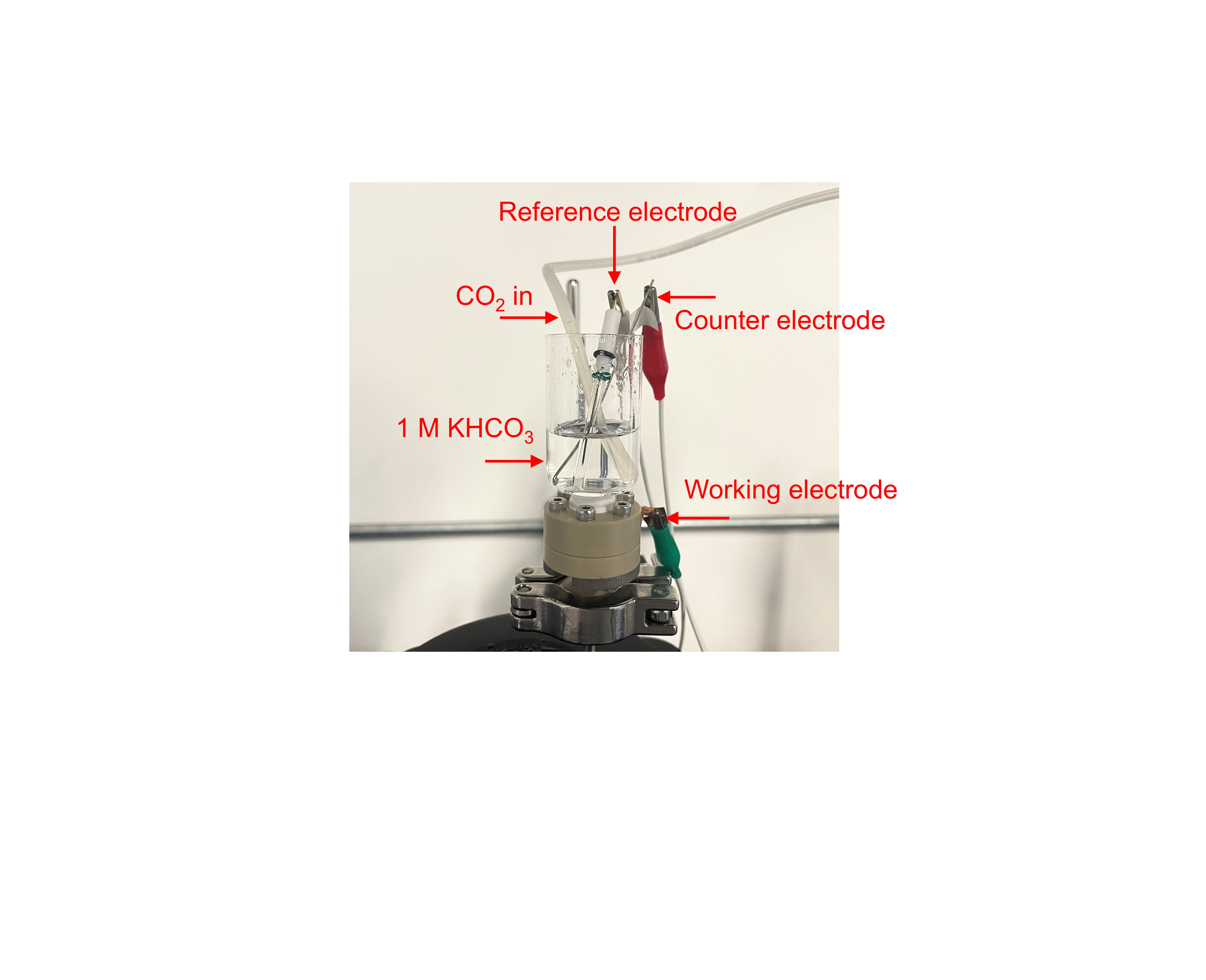


**Figure S46**. In situ electrochemical spectral cell for DEMS tests.


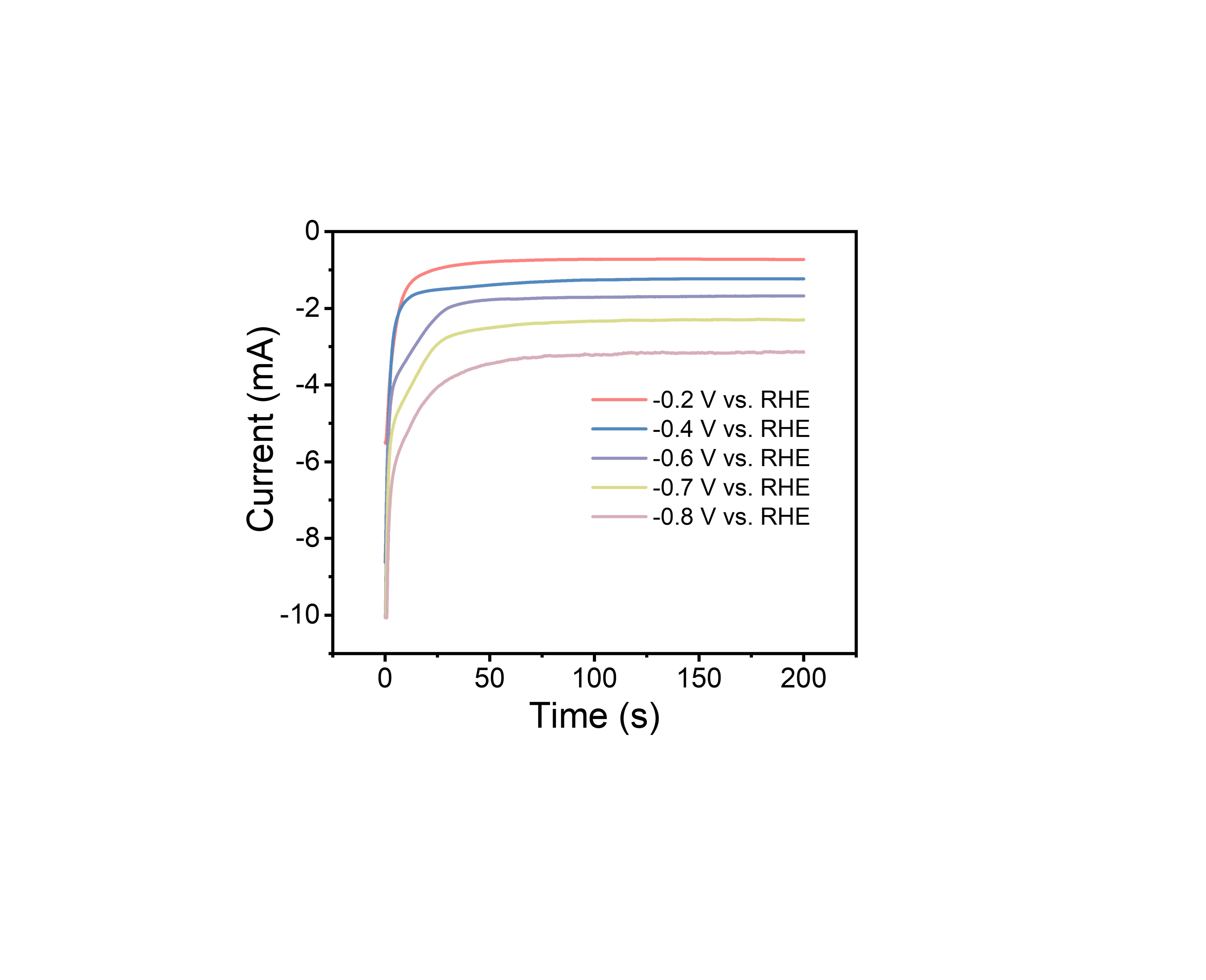


**Figure S47**. The Chronopotentiometry measurements were conducted in a CO_2_-saturated 1.0 M KHCO_3_ electrolyte using DEMS to simultaneously monitor potential-dependent reaction products.


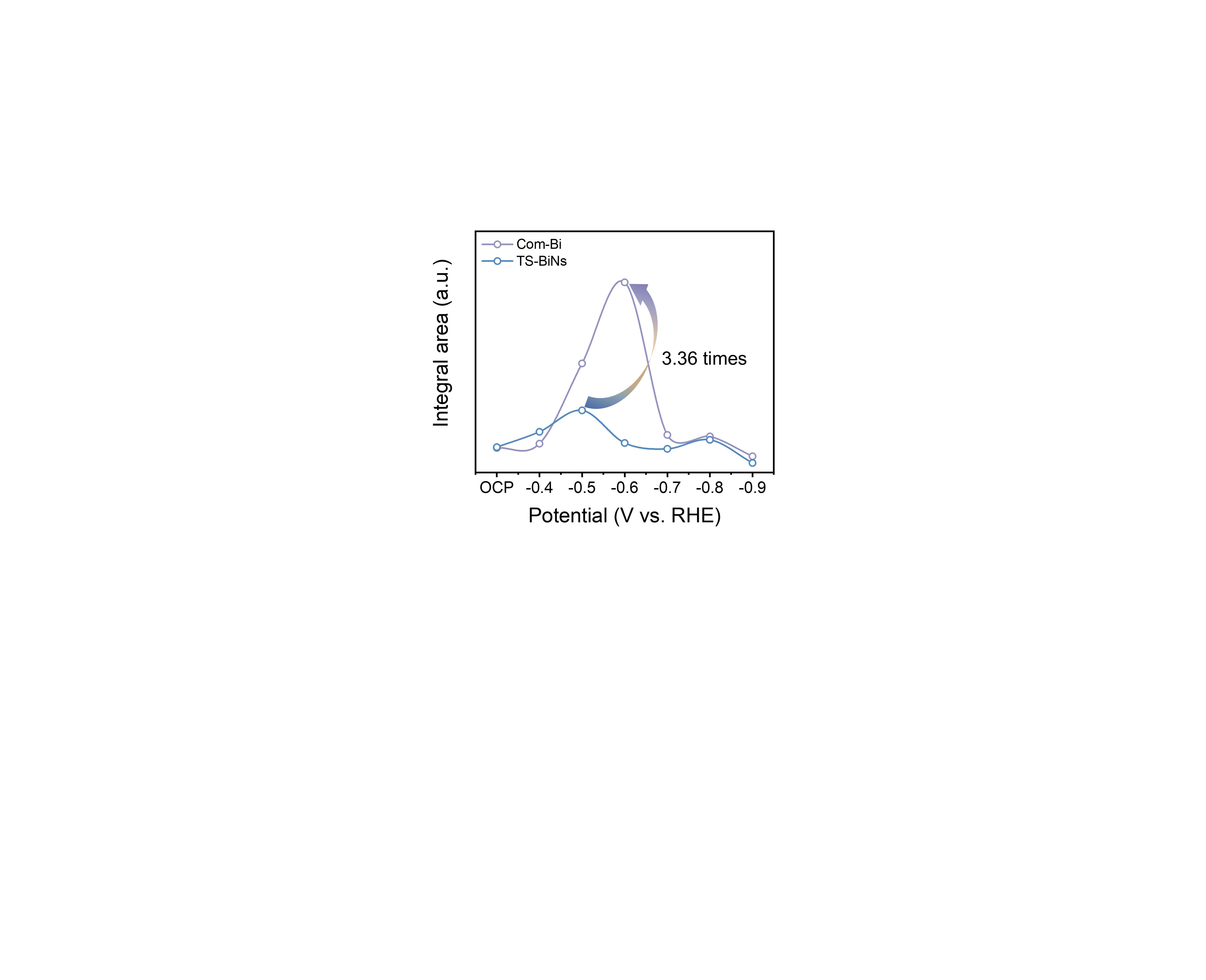


**Figure S48**. Integral areas of *OCHO during CO_2_RR for TS-BiNs and Com-Bi catalysts.


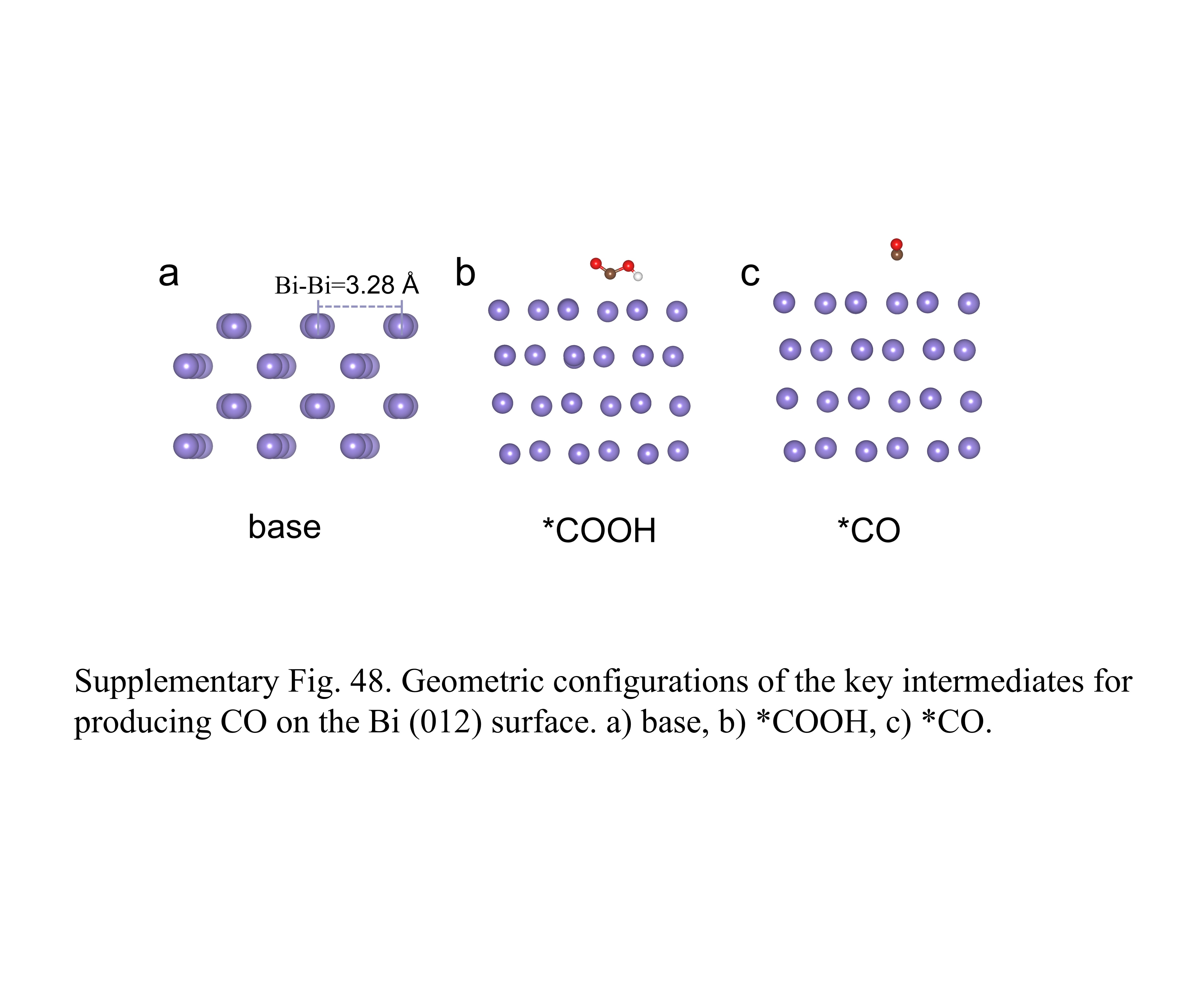


**Figure S49**. Geometric configurations of the key intermediates for producing CO on the Bi (012) surface. (a) base, (b) *COOH, (c) *CO.

**
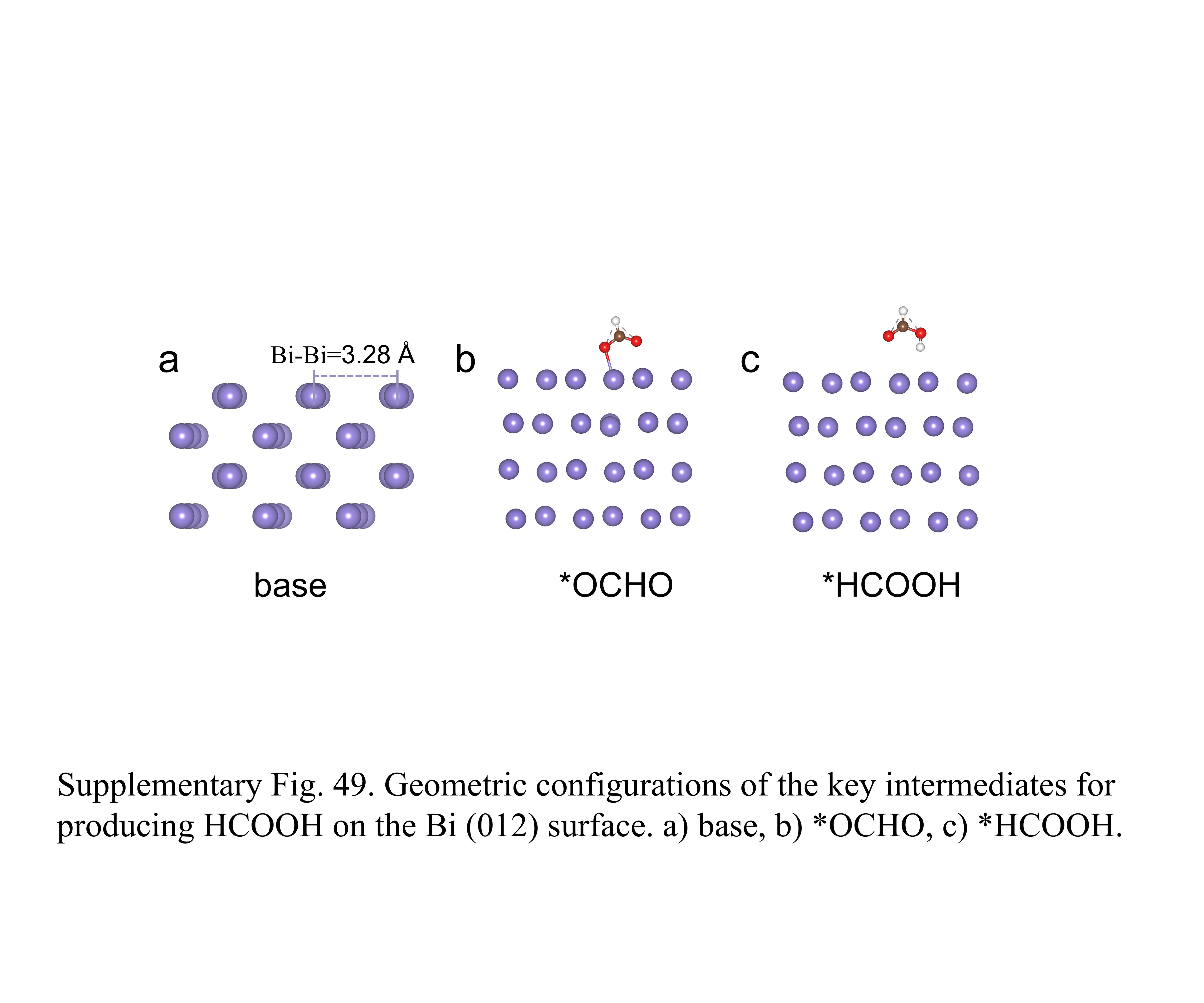
**

**Figure S50**. Geometric configurations of the key intermediates for producing HCOOH on the Bi (012) surface. (a) base, (b) *OCHO, (c) *HCOOH.


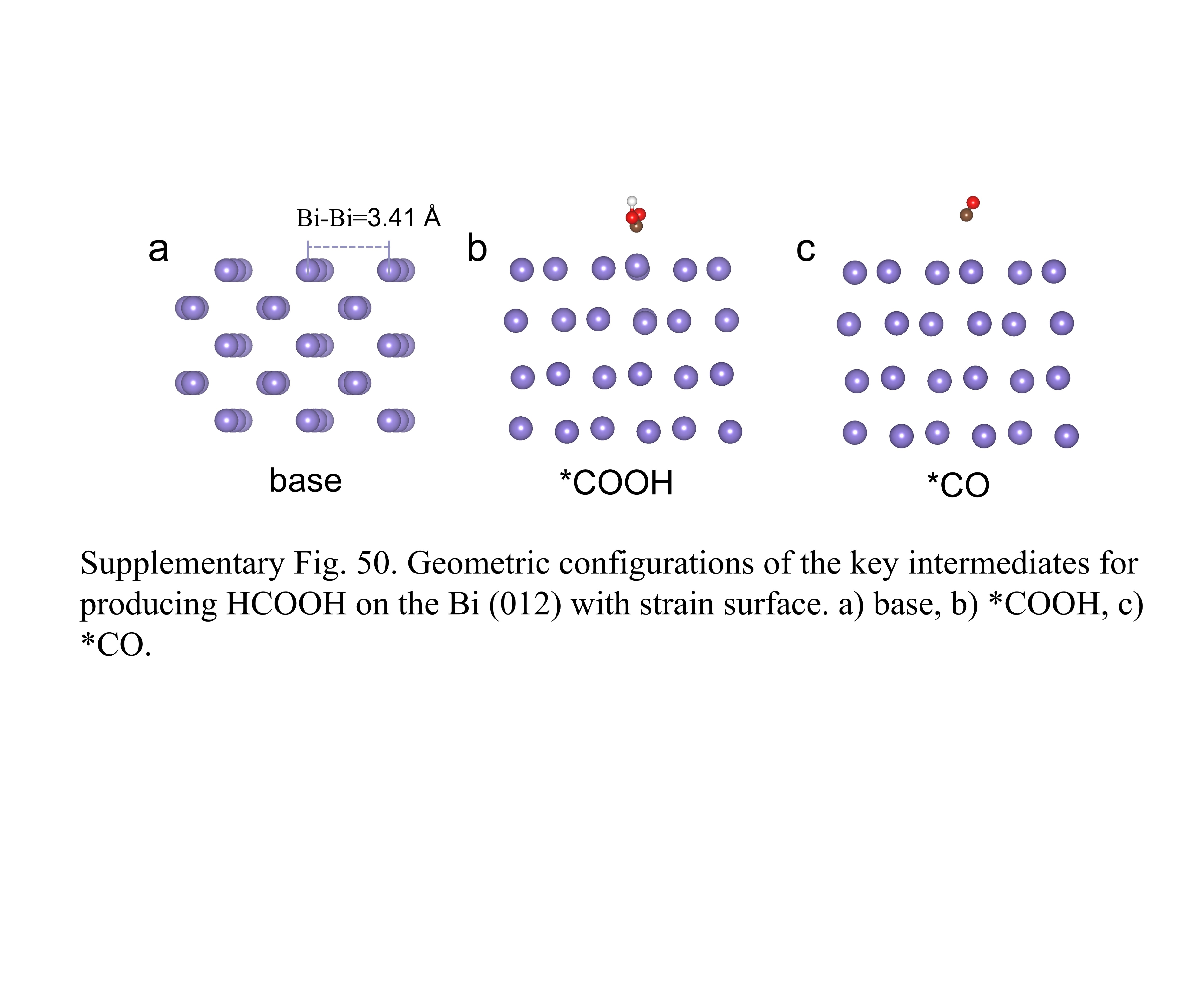


**Figure S51**. Geometric configurations of the key intermediates for producing HCOOH on the Bi (012) with tensile strain surface. (a) base, (b) *COOH, (c) *CO.


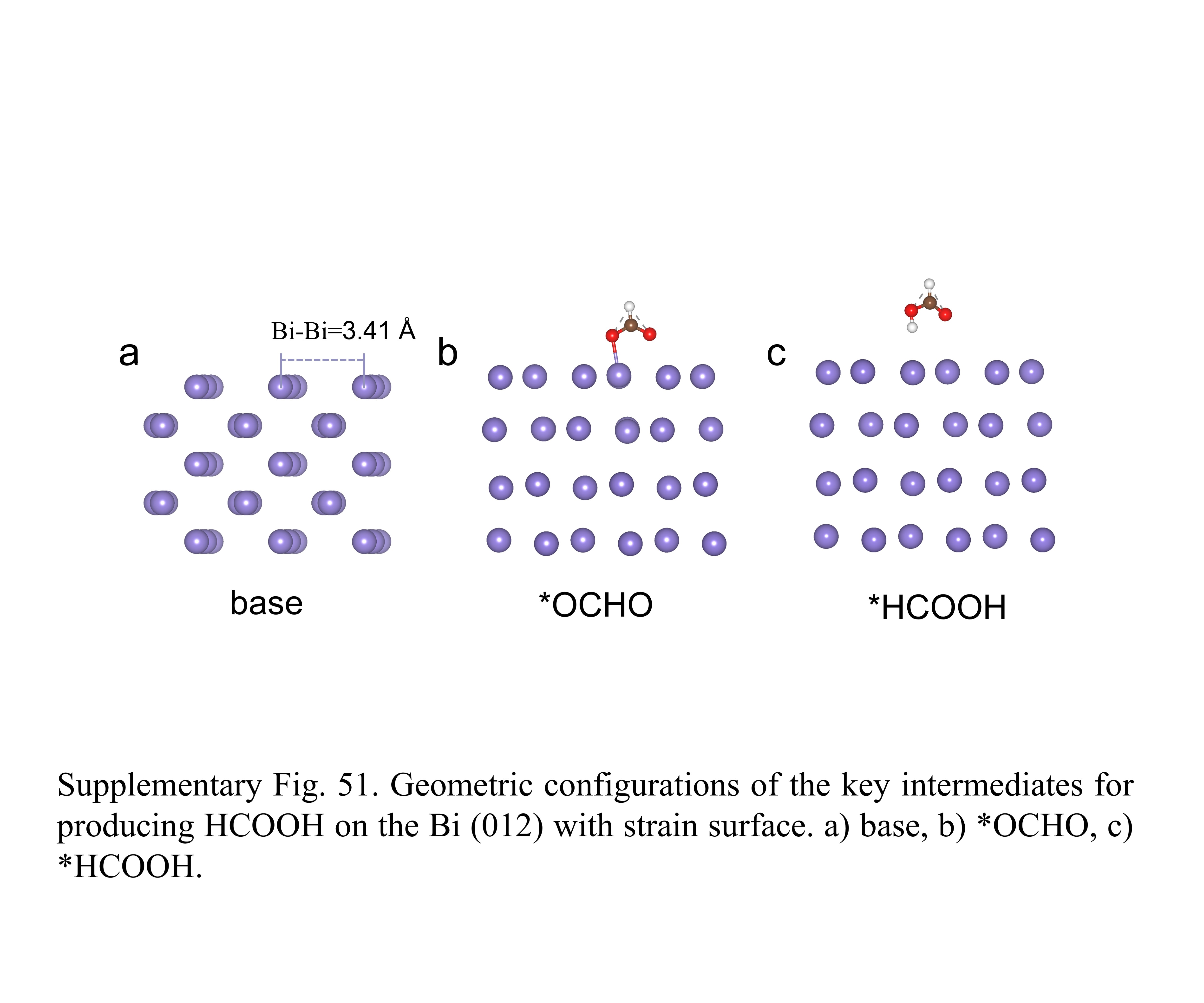


**Figure S52**. Geometric configurations of the key intermediates for producing HCOOH on the Bi (012) with tensile strain surface. (a) base, (b) *OCHO, (c) *HCOOH.


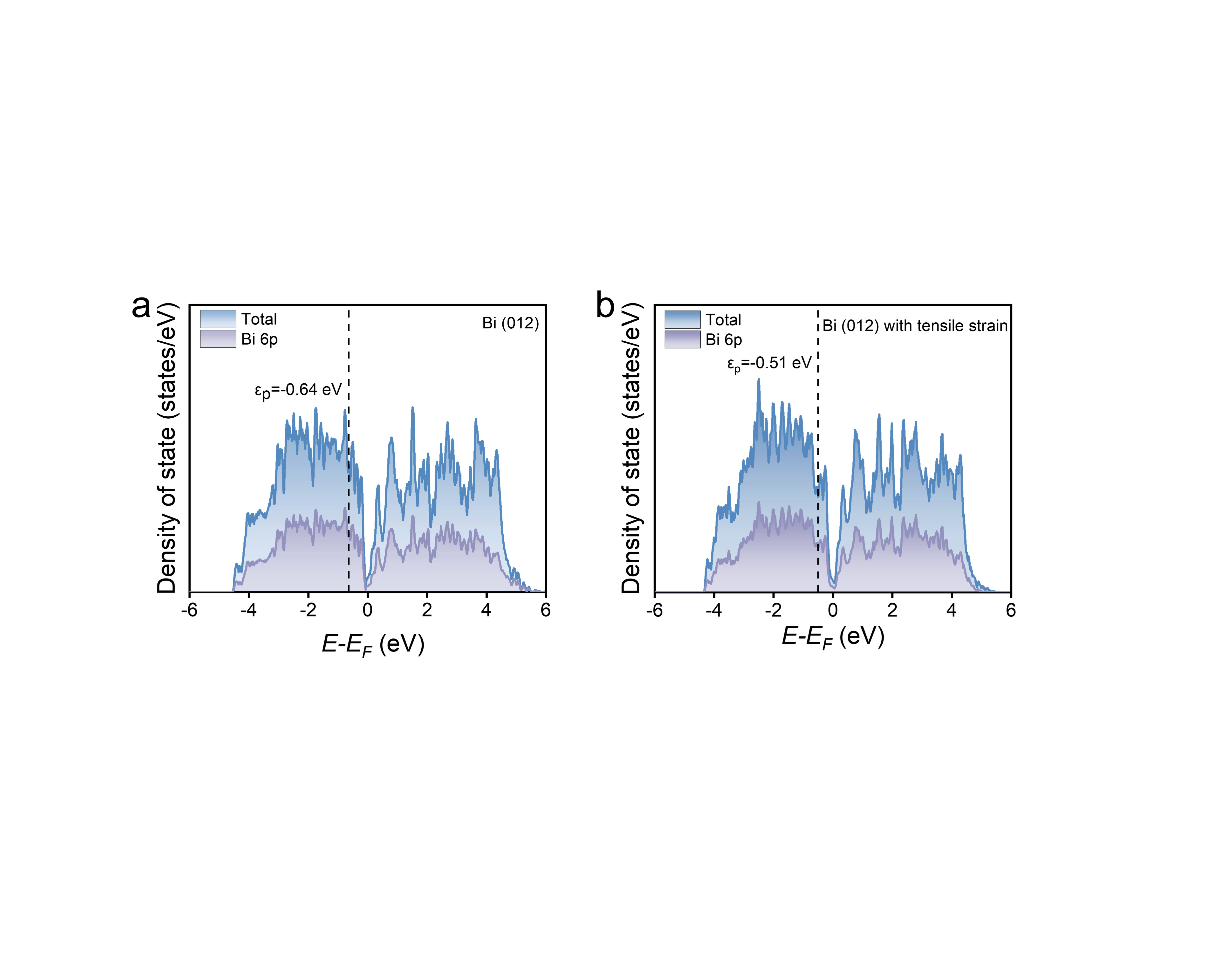


**Figure S53**. Density of states of the Total and Bi 6p in *OCHO intermediate on the (a) Bi (012) and (b) Bi (012) with tensile strain models.


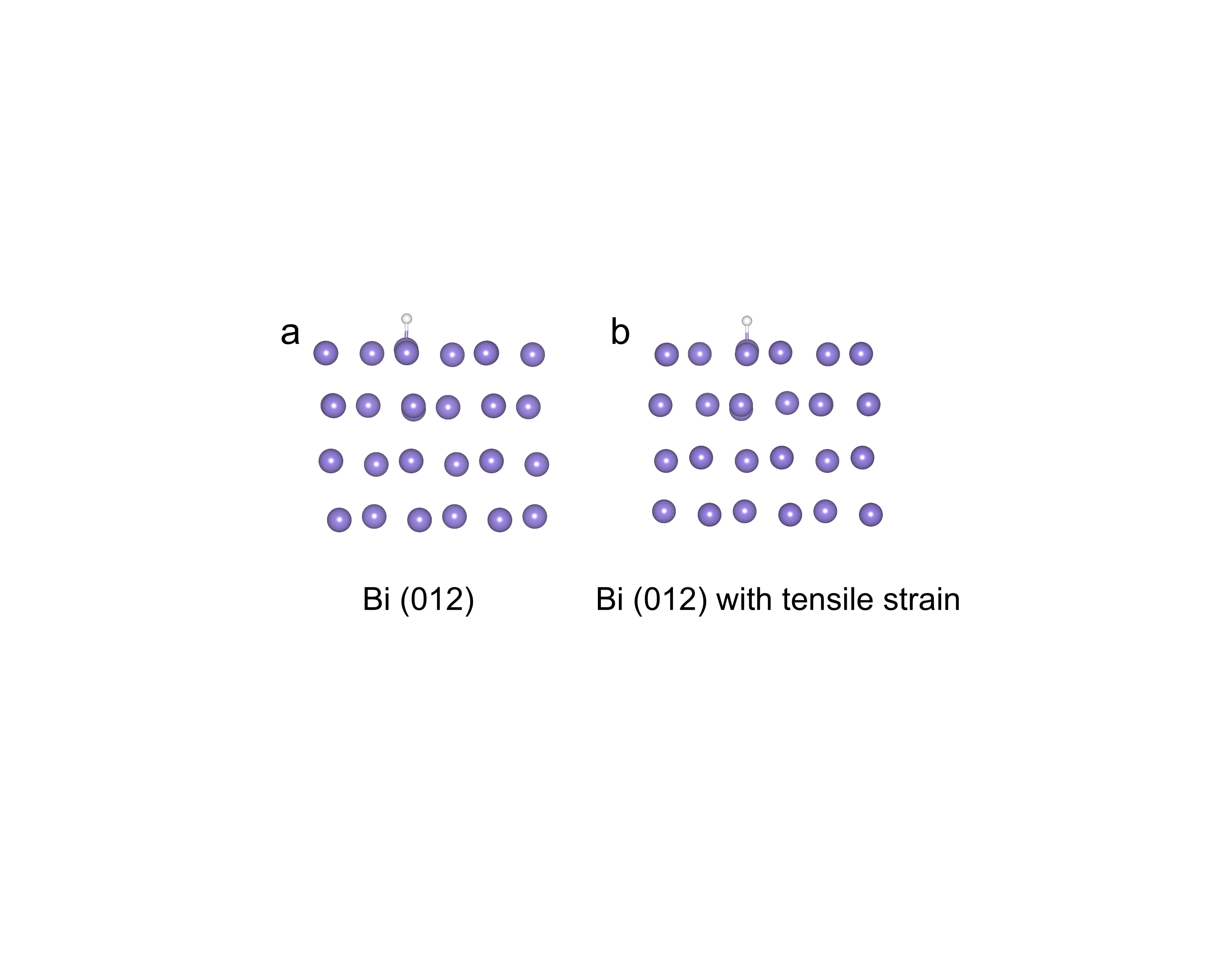


**Figure S54**. Geometric configurations of the intermediates for HER on the (a) Bi (012) and (b) Bi (012) with tensile strain models.

**Table S1.** EXAFS fitting parameters for Bi foil, Bi_2_O_3_ and TS-BiNs.

Note: S_0_^2^ was fixed as 0.90. Data ranges: 2≤ *k* ≤ 10 Å^-1^, 1 ≤ R ≤ 4 Å

| Sample | Path | N | R (Å) | σ^2^*10^-3^ (Å^2^) | ΔE_0_ (eV) | R factor |
| --- | --- | --- | --- | --- | --- | --- |
| Bi foil | Bi-Bi1 | 3 (set) | 3.05 | 2.84 | -0.36 | 0.005 |
|  | Bi-Bi2 | 2 (set) | 3.54 | 7.03 | -0.36 | 0.005 |
| Bi_2_O_3_ | Bi-O | 2.01 | 2.14 | 4.24 | -6.03 | 0.013 |
| TS-BiNs | Bi-O | 2.54 | 2.20 | 2.14 | -4.6 | 0.009 |

**Table S2**. Comparison of CO_2_RR performance of TS-BiNs with those previously reported catalysts for CO_2_RR to HCOO^–^ in the flow cell.

| **Catalysts** | **Electrolyte** | **Current density (mA cm^-2^)** | **FE (%)** | **Stability** | **References** |
| --- | --- | --- | --- | --- | --- |
| **TS-BiNs** | **1 M KHCO_3_** | **1000** | **92.2** | **105 h@100 mA cm^-2^** | **This work** |
| Bi_2_O_3_@C-800 | 1 M KOH | 208 | 93 | 1 h@150 mA cm^-2^ | Angew. Chem. Int. Ed. **2020**, 59, 10807-10813. |
| Bi_1,n-_*p*Bi_2_O_3-x_ | 1 M KOH | 200 | 96.5 | 100 h@30 mA cm^-2^ | Angew. Chem. Int. Ed. **2025**, 64, e202510206. |
| Bi-DC | 1 M KHCO_3_ | 403 | 93.7 | 20 h@250 mA cm^-2^ | ACS Catal. **2024**, 14, 8050. |
| BiOBr | 1 M KHCO_3_ | 162.9 | 91 | N/A | Nat. Catal. **2023**, 6, 796. |
| Bi-ene-NW | 1 M KOH | 570 | 91 | 200 h@110 mA cm^-2^ | Energy Environ. Sci. **2021**, 14, 4998. |
| Bi_2_O_3_ NTs | 1 M KHCO_3_ | 140 | 97 | 13 h@140 mA cm^-2^ | Nat. Commun. **2019**, 10, 2807. |
| nBuLi-Bi | 1 M KHCO_3_ | 500 | 90 | N/A | Nat. Commun. **2020**, 11, 3633. |
| Leafy Bi nanosheets | 1 M KHCO_3_ | 429 | 86.9 | 10 h@128 mA cm^-2^ | Adv. Energy Mater. **2020**, 10, 2001709. |
| BOC/Bi-3 | 1 M KOH | 600 | 90.5 | 10 h@200 mA cm^-2^ | Adv. Funct. Mater. **2024**, 34, 2400928. |
| Bismuthene nanosheets | 1 M KHCO_3_ | 240 | 97 | 25 h@50 mA cm^-2^ | Adv. Funct. Mater. **2021**, 31, 2006704. |
| BBS | 1 M KOH | 200 | 95 | 100 h@250 mA cm^-2^ | Angew. Chem. Int. Ed. **2023**, 62, e202214959. |
| Bi(OH)_3_-after | 0.5 M KHCO_3_ | 1000 | 95 | N/A | Adv. Mater. **2025**, 37, 2415639. |
| BiNN-CF | 1 M KOH | 419 | 93 | 12 h@420 mA cm^-2^ | Adv. Energy Mater. **2022**, 12, 2103960. |
| Bi_2_S_3_-derived | 1 M KOH | 2000 | 93 | N/A | Angew. Chem. Int. Ed. **2023**, 62, e202303117. |
| RD-Bi | 1 M KOH | 1200 | 94.2 | 150 h@200 mA cm^-2^ | Angew. Chem. Int. Ed. **2024**, 63, e202317628. |
| RC-Bi_2_O_3_-60 | 1 M KOH | 1000 | 96 | 40 h@60 mA cm^-2^ | Angew. Chem. Int. Ed. **2024**, 63, e202316640. |
| BS/VC | 1 M KOH | 968 | 94 | N/A | Nat. Commun. **2023**, 14, 4670. |
| Bi RDs | 1 M KOH | 290.1 | 92.2 | 20 h@200 mA cm^-2^ | Adv. Mater. **2021**, 33, 2008373. |
| Bi Ns-L | 1 M KOH | 275 | 89.1 | 15 h@100 mA cm^-2^ | Nano Lett. **2025**, 25,10152−10160. |
| SnNCN | 1 M KHCO_3_ | 450 | 97.4 | 110 h@200 mA cm^-2^ | Angew. Chem. Int. Ed. **2025**, 64, e202507422 |
| Zn_1_Sn_1_/SNC DASs | 0.5 M KHCO_3_ | 300 | 90.6 | 120 h@200 mA cm^-2^ | Nat. Commun. **2025**, 16, 2217 |
| Bi-MOF-TS | 0.1 M K_2_SO_4_ +0.02 M H_2_SO_4_ | 1000 | 87 | 60 h@500 mA cm^-2^ | Nat. Commun. **2025**, 16, 1927 |
| CuBi-R | 1 M KHCO_3_ | 300 | 93 | 80 h@100 mA cm^-2^ | Proc. Natl. Acad. Sci. U.S.A. **2024**, 121, e2400898121 |

**Table S3**. Comparison of CO_2_RR performance of TS-BiNs with reported catalysts for CO_2_RR to HCOOH in the solid-state electrolyte.

| Catalysts | Current density  (mA cm^–2^) | Concentration of HCOOH (M) | Duration  (h) | References |
| --- | --- | --- | --- | --- |
| **TS-BiNs** | **100** | **0.18** | **100** | **This work** |
|  | **100** | **0.25** | **80** |  |
|  | **100** | **0.30** | **85** |  |
| Pb_1_Cu | 100 | 0.10 | 180 | Nat. Nanotechnol. **2021**, 16, 1386–1393. |
| 2D-Bi | 30 | 0.11 | 100 | Nat. Energy **2019**, 4, 776–785. |
| nBuLi-Bi | 30 | 0.10 | 100 | Nat. Commun. **2020**, 11, 3633. |
| Ns-Bi_2_O_3_ | 56 | 0.1 | 43 | CCS Chem. **2024**, 6, 100–109. |
| Bi-MSs | 160 | 0.13 | 110 | Appl. Catal. B-Environ. **2023**, 322, 122127. |
| Co_0.05_-BOON | 100 | 0.1 | 100 | Angew. Chem. Int. Ed. **2024**, 63, e202403671. |
| Bi-MOF-TS | 50 | N/A | 24 | Nat Commun. **2025**, 16, 1927. |
| Bi-Cu/HMCS | 100 | 0.6 | 7.5 | Nano Lett. **2023**, 23, 10946–10954. |
| BS/VC | 100 | N/A | 80 | Nat. Commun. **2023**, 14, 4670. |
| In_2_O_3_/C | 30 | 0.12 | 3 | Angew. Chem. Int. Ed. **2022**, 61, e202200552. |

**Table S4.** Feedstocks and product cost.

| Feedstocks | Product cost |
| --- | --- |
| CO_2_ ($/ton) | 50^16^ |
| High purity H_2_O ($/ton) | 0.7^16^ |
| O_2_ ($/ton) | 50^16^ |
| HCOOH ($/ton) (Q2 2025) | 956^17^ |

**Note S1:**

**Techno-economic assessment of the CO_2_RR system to produce HCOOH. Supplementary Table 4** details the pricing of chemicals used in this study. Technical and economic analyses were performed using established models from references^11-13^. We assumed an annual operation of 350 days with a daily production rate of 1 ton of HCOOH. The Faraday constant is 96,485 C per mole of electrons. Our TEA parameters are based on experimental data. To estimate the operational voltage for a potential industrial electrolyzer, we used an experimentally derived SSE reactor voltage of 3.2 V and a Faradaic efficiency of 97% for HCOOH at a current density of 100 mA cm^–2^.

The number of coulombs Q required to recover 1 ton of HCOOH per day would be approximately:

$$Q=n*z*\frac{F}{\mathrm{FE}\left( \mathrm{HCOOH} \right)}=\frac{1 \mathrm{ton}*1,000,000 \frac{g}{\mathrm{ton}}}{46 \frac{g}{\mathrm{mol}}}*2*96,485 \frac{C}{\mathrm{mol}}*\frac{1}{97\%}=4,324,742,268 C$$

Where n is the mole of HCOOH. z is the electron number. F is the Faradaic constant of 96,485 C/mol. FE is the Faraday efficiency of HCOOH.

The electrode area *S* is:

$$S=\frac{\frac{Q}{t}}{j}=\frac{\frac{4,324,742,268 C}{86,400 s}}{0.1 \frac{A}{cm^{2}}}=50.05m^{2}$$

1. Electrolyzer and catalyst cost

From the DOE H_2_A analysis for central grid electrolysis, the electrolyzer cost for the stack component is $250.25/kW with a reference current density of 175 mA cm^-2^ @1.75 V^4^. Thus, the total electrolyzer cost is:

$$Electrolyzer cost= electrolyzer area*Reference cost*Reference current density*\frac{A}{1,000mA}*\frac{\mathrm{kW}}{1,000W}Reference voltage*{10}^{4}\frac{cm^{2}}{m^{2}}$$

$$Electrolyzer cost=50.05 m^{2}*250.25 \$/kW*\frac{175 mA cm^{-2}*1.75 V}{1,000*1,000}*10,000=\$ 38,358$$

Herein, the capital recovery factor (CRF) is based on a discount rate (denoted *r*; we use 5% for all the CRF calculations) and the material lifetime. All calculations are based on the assumption that the factory operates with a capacity factor of 0.9, which represents the ratio of actual production to the maximum potential production capacity.

$$CRF=\frac{r*{(r+1)}^{\mathrm{year}}}{{(r+1)}^{\mathrm{year}}-1}=\frac{0.05*{1.05}^{20}}{{1.05}^{20}-1}=0.0802$$

CRF _Electrolyzer_=$\$ 38,358*\frac{0.0802}{350*0.9}=\$ 9.77$

The catalysts and membrane cost, we assume that their total one-time cost is 5% of the electrolyzer cost with a lifetime of 5 years.

$$CRF=\frac{r*{(r+1)}^{\mathrm{year}}}{{(r+1)}^{\mathrm{year}}-1}=\frac{0.05*{1.05}^{5}}{{1.05}^{5}-1}=0.231$$

CRF _Catalysts and membrane_ =$\$ 38,358*0.231*\frac{0.05}{350*0.9}=\$ 1.41$

2. Electricity cost

The electricity cost is calculated from the power requirement and the price of electricity, assuming the electricity price is 2 cents/kWh:

Electricity cost _per ton HCOOH_$=UQ\left( \mathrm{HCOOH} \right)*\$ 0.02=3.2 V*4,324,742,268 C*\frac{1}{V*C}*1 J*\frac{\mathrm{kWh}}{3,600,000 J}*\frac{\$ 0.02}{\mathrm{kWh}}=\$ 76.88$

3. Input chemicals cost

For the input chemicals cost, we account for the cost of the consumed H_2_O. The water price is estimated as $ 0.7 per ton^14^.

H_2_O cost _per ton HCOOH_ =$\frac{Q(HCOOH)}{\mathrm{zF}}*M*\$$ 0.7=$\frac{4,324,742,268 C}{2*96,485 \frac{C}{\mathrm{mol}}}*18 \frac{\frac{g}{\mathrm{mol}}}{1,000,000 \frac{g}{\mathrm{ton}}}*\$ 0.7=\$ 0.28$

For the input chemicals cost, we account for the cost of the consumed CO_2_. The CO_2_ price is estimated as $ 50 per ton^14^.

CO_2_ cost _per ton HCOOH_ =$\frac{Q(HCOOH)}{\mathrm{zF}}*M*\$$ 50=$\frac{4,324,742,268 C}{2*96,485 \frac{C}{\mathrm{mol}}}*44 \frac{\frac{g}{\mathrm{mol}}}{1,000,000 \frac{g}{\mathrm{ton}}}*\$ 50=\$ 49.31$

4. Balance of plant (BOP) cost and Installation

To estimate the costs of peripheral equipment associated with the electrolyzer and separation units, we scale all capital costs accordingly. A balance of plant (BOP) factor of 50% and a Lang factor of 1 are assumed^15^. To calculate the total capital costs, we aggregate the individual costs of the electrolyzer, membrane, and catalyst capital.

Cost$\left( Total capital \right)$ = Electrolyzer _per ton HCOOH_ + Catalysts and membrane _per ton HCOOH_=$\$ 9.77$+$\$ 1.41$=$\$$11.18

Cost$\left( \mathrm{BOP} \right)$ = 0.5$\times$Cost$\left( Total capital \right)$=$\$ 11.18\times0.5$=$\$$5.59

Cost$\left( \mathrm{Installation} \right)=Lang Factor\times\mathrm{Cost}_{Total Capital}=1\times\$ 11.18\mathbf{=}\$$11.18

5. Other operational costs

Other operational costs, such as labor and maintenance are assumed to be 2.5% of the electrolyzer cost per ton of HCOOH:

Costs (Other) _per ton HCOOH_=$\$ 38,358*\frac{0.025}{350}=\$ 2.74$

6. Cathode gas separation costs

To facilitate the recirculation of unused CO_2_, the gaseous side product (H_2_) is separated from the outlet stream of CO_2_^15^.

$$\text{H}_{\text{2}}\text{ production rate }\left( \frac{\text{mol}}{\text{s}} \right)\text{=}\frac{\text{Q}\text{× }\text{FE}_{\text{H2}}}{\text{2 }\text{e}^{\text{-}}\text{*F}}\text{= }\frac{\text{4,324,742,26}\text{×}\text{3}\text{\%}}{\text{2 }\text{e}^{\text{-}}\text{× 96485 }\frac{\text{C}}{\text{mol}}}\text{=}\text{67.23}\text{ }\frac{\text{mol}}{\text{s}}$$

$$\text{H}_{\text{2}}\text{ Flow Rate }\left( \frac{\text{m}^{\text{3}}}{\text{h}} \right)\text{=}\frac{\text{H}_{\text{2}}\text{ production rate ×R×T }}{\text{P}}\text{=}\frac{\text{67.23 }\text{×8.314 ×298}}{\text{1}}\text{=}\text{46.27}\text{ }\left( \frac{\text{m}^{\text{3}}}{\text{h}} \right)$$

$$\text{CO}_{\text{2}}\text{ unreacted flow rate }\left( \frac{\text{mol}}{\text{s}} \right)\text{=}\frac{\text{CO}_{\text{2}}\text{ input rate ×}\text{90\%}}{\text{M}_{\text{CO2}}\text{×}\text{time}}\text{=}\frac{\text{1000}\text{,}\text{00}\text{0}\text{×}\text{8}\text{0}\text{\%}}{\text{44}\text{×}\text{3600}}\text{=5.05}\frac{\text{mol}}{\text{s}}$$

$$\text{ }\text{output }\text{f}\text{low }\text{r}\text{ate }\left( \frac{\text{m}^{\text{3}}}{\text{h}} \right)\text{=}\frac{\text{CO}_{\text{2}}\text{ unreacted flow rate ×R×T }}{\text{P}}\text{=}\frac{\text{5.05}\text{×8.314×298}}{\text{1}}\text{=}\text{3.48}\text{ }\left( \frac{\text{m}^{\text{3}}}{\text{h}} \right)$$

The sum of the gaseous side product flow rate and the flow rate of unreacted CO_2_ is used to estimate the gaseous separation cost:

The total flow rate $\left( \frac{\text{m}^{\text{3}}}{\text{h}} \right)$=$\text{H}_{\text{2}}\text{ Flow Rate }\left( \frac{\text{m}^{\text{3}}}{\text{h}} \right)\text{+CO}_{\text{2}}\text{ }\text{u}\text{nreated Flow Rate=}\text{49.75}\text{ }\left( \frac{\text{m}^{\text{3}}}{\text{h}} \right)$

$$\text{Gas Separation Operational Cost }\left( \frac{\$}{\text{tonne}} \right)\text{=}\frac{\text{total flow rate× reference PSA cost×electricity price}}{\text{target output production}}\text{=}\text{49.75}\text{ }\frac{\text{m}^{\text{3}}}{\text{h}}\text{×24}\text{ }\frac{\text{h}}{\text{day}}\text{×0.25 }\frac{\text{kWh}}{\text{m}^{\text{3}}}\text{ ×0.0}\text{2}\text{ }\frac{\$}{\text{kWh}}\text{÷1 }\frac{\text{tonne FA}}{\text{day}}\text{=}\frac{\$ \text{5.97}}{\text{ton FA}}$$

$$\text{Gas Separation Capital Cost }\left( \frac{\$}{\text{tonne}} \right)\text{=}\frac{\text{reference PSA capital cost×}{\text{(}\frac{\text{total flow rate}}{\text{reference capacity}}\text{)}}^{\text{scaling factor}}\text{×CRF}}{\text{Capacity factor×365×target output production}}\text{=}\frac{\$ \text{1989043}\text{×}{\text{(}\frac{\text{49.75}\frac{\text{m3}}{\text{h}}}{\text{10}\text{00}\text{0 m3/h }}\text{)}}^{\text{0.7}}\text{×0.0}\text{802}}{\text{0.9×3}\text{50}\frac{\text{day}}{\text{year}}\text{×1 }\frac{\text{tonne FA}}{\text{day}}}\text{=}\frac{\$ \text{12.36}}{\text{ton FA}}$$

$$\text{Total Gas Separation Cost }\left( \frac{\$}{\text{tonne}} \right)\text{= Gas Separation Operational Cost}\text{ }\left( \frac{\$}{\text{tonne}} \right)\text{+Gas Separation Capital Cost }\left( \frac{\$}{\text{tonne}} \right)\text{=}\frac{\$ \text{18.33}}{\text{tonne FA}}$$

7. Formic acid purification costs

To account for that, we have added the costs to bring the product concentration to 100 wt. % to our calculations. We have used a reference cost of $5 ton^−1^ H_2_O removal via portable reverse osmosis units^18^:

H_2_O to be removed =$\frac{1000 kg HCOOH}{7 wt. \%}-1000 {Kg}_{HCOOH}-1000 {Kg}_{H2O}=12.28 ton$

Purification costs =$5\frac{\$}{\mathrm{ton}}*12.28 ton=\frac{\$ \text{61.4}}{\text{tonne FA}}$

8. Potential profit from selling O_2_

Assuming O_2_ Faradaic efficiency of 100%. Then, the profit from capture 1 ton of HCOOH in terms of O_2_ would be:

Profit$\left( O_{2} \right)$ _per ton HCOOH_ =$\frac{4,324,742,268 C}{4*96,485 \frac{C}{\mathrm{mol}}}*\frac{32 \frac{g}{\mathrm{mol}}}{1,000,000 \frac{g}{\mathrm{ton}}}*\frac{\$}{t} 50=\$ 17.93$

9. Total cost for one ton of HCOOH

By summing all the above costs, we obtain the cost of producing one ton of HCOOH in an electrolyzer:

$$\mathrm{Cost}_{\mathrm{FA}}= \mathrm{Cost}_{\mathrm{electricity}}+\mathrm{Cost}_{Chemcial imput}+\mathrm{Cost}_{Electrolyzer and catalyst}+\mathrm{Cost}_{B\mathrm{OP}}+Cost \left( \mathrm{Installation} \right)+\mathrm{Cost}_{\mathrm{other}}+\mathrm{Cost}_{gas separation}+\mathrm{Cost}_{\mathrm{purification}}-{P\mathrm{rofit}}_{o2}=76.88+49.59+11.18+5.59+11.18+2.74+18.33+61.4-17.93\mathbf{=}\frac{\boldsymbol{\$}\mathbf{218.96}}{\mathbf{ton H}\mathbf{COOH}}$$

**References**

1. Hÿtch, M. J., Snoeck, E. & Kilaas, R. Quantitative measurement of displacement and strain fields from HREM micrographs. *Ultramicroscopy* **74**, 131–146 (1998).
2. Kresse, G. & Furthmüller, J. Efficient iterative schemes for ab initio total-energy calculations using a plane-wave basis set. *Phys. Rev. B* **54**, 11169–11186 (1996).
3. Blöchl, P. E. Projector augmented-wave method. *Phys. Rev. B* **50**, 17953–17979 (1994).
4. Perdew, J. P., Burke, K. & Ernzerhof, M. Generalized gradient approximation made simple. *Phys. Rev. Lett.* **77**, 3865–3868 (1996).
5. Grimme, S., Antony, J., Ehrlich, S. & Krieg, H. A consistent and accurate ab initio parametrization of density functional dispersion correction (DFT-D) for the 94 elements H-Pu. *J. Chem. Phys.* **132**, 154104 (2010).
6. Bader, R. F. W. Atoms in molecules. *Acc. Chem. Res.* **18**, 0001–4842 (1985).
7. Maintz, S., Deringer, V. L., Tchougréeff, A. L. & Dronskowski, R. LOBSTER: A tool to extract chemical bonding from plane‐wave based DFT. *J Comput Chem* **37**, 1030–1035 (2016).
8. Wang, V., Xu, N., Liu, J.-C., Tang, G. & Geng, W.-T. VASPKIT: A user-friendly interface facilitating high-throughput computing and analysis using VASP code. *Comput. Phys. Commun.* **267**, 108033 (2021).
9. Nørskov, J. K. *et al.* Origin of the overpotential for oxygen reduction at a fuel-cell cathode. *J. Phys. Chem. B* **108**, 17886–17892 (2004).
10. Peterson, A. A., Abild-Pedersen, F., Studt, F., Rossmeisl, J. & Nørskov, J. K. How copper catalyzes the electroreduction of carbon dioxide into hydrocarbon fuels. *Energy Environ. Sci.* **3**, 1311–1315 (2010).
11. Shin, H., Hansen, K. U. & Jiao, F. Techno-economic assessment of low-temperature carbon dioxide electrolysis. *Nat. Sustain.* **4**, 911-919, (2021).
12. Wang, X. *et al.* Efficient electrosynthesis of n-propanol from carbon monoxide using a Ag–Ru–Cu catalyst. *Nat. Energy.* **7**, 170-176, (2022).
13. Zhu, P. *et al.* Continuous carbon capture in an electrochemical solid-electrolyte reactor. *Nature* **618**, 959-966, (2023).
14. Khan, M. A. *et al.* Seawater Electrolysis for Hydrogen Production: a Solution Looking for a Problem? *Energy. Environ. Sci.* **14**, 4831-4839, (2021).
15. Fang, W., Guo, W., Lu, R. et al. Durable CO_2_ conversion in the proton-exchange membrane system. *Nature* **626**, 86-91 (2024).
16. Wang, P. *et al.* Integrated system for electrolyte recovery, product separation, and CO_2_ capture in CO_2_ reduction. *Nat. Commun.* **16**, 731 (2025).
17. https://www.chemanalyst.com/Pricing-data/formic-acid-1242.
18. Okatenko, V., Elgazzar, A., Loiudice, A. et al. Energy-efficient indirect (bi)carbonate electroreduction in a porous solid electrolyte reactor. *Nat. Sustain.* (2026). https://doi.org/10.1038/s41893-025-01755-x.
